# Trends in the Prevalence of Autism Spectrum Disorder in California: Disparities by Sociodemographic Factors and Region Between 1990–2018

**DOI:** 10.1007/s10803-024-06371-w

**Published:** 2024-05-03

**Authors:** Karl O’Sharkey, Sanjali Mitra, Seung-a Paik, Ting Chow, Myles Cockburn, Beate Ritz

**Affiliations:** 1 Department of Epidemiology, Fielding School of Public Health, University of California, Los Angeles, CA 90095 USA; 2 Department of Population and Public Health Sciences, University of Southern California, Los Angeles, CA 90032 USA; 3 Department of Environmental Health Sciences, Fielding School of Public Health, University of California, Los Angeles, CA 90095 USA; 4 Department of Neurology, David Geffen School of Medicine, University of California, Los Angeles, CA 90095 USA

**Keywords:** Autism, ASD, Disparities, Incidence

## Abstract

Autism Spectrum Disorders (ASD) prevalence has risen globally, with regional variation and sociodemographic disparities affecting diagnosis and intervention. This study examines ASD trends from 1990 to 2018 in California (CA), focusing on sociodemographic factors that may inform policy/interventions. Using CA Department of Public Health birth records (1990–2018) and Developmental Services ASD cases (1994–2022), we analyzed diagnosis incidence by age 4 or 8, stratified by sociodemographic and regional factors. From 1990 to 2018, for each birth year the cumulative incidence of ASD by 4 and 8 years of age in CA increased while the diagnosis age decreased. Distinct patterns emerged over these three decades. Children born to White and Asian and Pacific Islander (API) mothers, or to mothers with higher education or living in high socioeconomic status (SES) neighborhoods exhibited higher ASD cumulative incidences throughout the 1990s and early 2000s. However, in the mid-2000s, ASD incidence in children born to Black or Hispanic mothers, with low education, or living in low SES neighborhoods surpassed that of White/API children or those living in high SES neighborhoods. Black or Hispanic children now have the highest ASD cumulative incidence, even though age at first diagnosis remains lowest in high SES regions, for the highly educated, and for White/API children. ASD cumulative incidence in CA from 1990 to 2018 exhibited demographic reversals with higher rates in children born to Black or Hispanic mothers or lower SES neighborhoods. Black and Hispanic children still have delayed age at diagnosis compared to White/API children.

**Supplementary Information:**

The online version contains supplementary material available at 10.1007/s10803-024-06371-w.

## Background

Autism Spectrum Disorder (ASD) is a complex neurodevelopmental condition that affects communication, behavior, and social interaction, starting and often recognized in the earliest years of life (American Psychiatric Association, 2020). In 2020, the United States (US) Center for Disease Control (CDC) estimated that 2.8% of children had been diagnosed with ASD by age 8 years, with California (CA) observing one of the highest prevalences at 4.5% (Maenner, 2023). Over the past few decades, the prevalence of ASD has shown a notable increase worldwide, and the average time to and age at diagnosis has decreased considerably (Zeidan et al., 2022). Increasing ASD has led to an increase in health-related burdens, notably, escalating direct and indirect economic costs affecting families and society at large (Buescher et al., 2014; C.D.C., 2009; Maenner, 2021). Disease burden may not be uniform across regions, require tailored interventions, and policy-makers to allocate resources according to need.

In addition to regional variation, differences in ASD prevalence exist by key sociodemographic factors, such as the sex of the infant, with a roughly 4:1 ratio of boys to girls (Maenner, 2023). There are also disparities by race and ethnicity with non-Hispanic Black children currently having the highest prevalence of ASD [2.85% (95% CI, 2.36-3.33%)] in the US in 2020 compared to non-Hispanic White children [2.65% (95% CI, 2.40-2.90%)], and Hispanic children [1.94% (95% CI, 1.64-2.24%)] (Yuan et al., 2021). This was not always the case, with White children having the highest prevalence until the mid-2000s (Nevison & Parker, 2020). Variations in the autism phenotype with regard to intellectual and language abilities by race/ethnicity may be related to underlying causes of ASD or disparities in diagnostic and treatment-related factors, further highlighting the need for temporal, regional, and sociodemographic trends to identify hotspots or underserved groups and/or regions (Esler et al., 2017; Jarquin et al., 2011; Pedersen et al., 2012).

Temporal changes in incidence and/or prevalence of ASD have been subject to debate and scrutiny, partly due to changes in diagnostic criteria, increased awareness, and improved diagnostic practices over time, although the increasing incidence over time does not appear to be fully explained by greater screening or expanding the case definition alone (Hertz-Picciotto & Delwiche, 2009). Instead, differences may occur because of a complex mixture of genetic, social, and regional factors, including access to medical treatment and diagnosis centers, and environmental exposures, which are often disparate by sociodemographics and region (Aylward et al., 2021; Liu et al., n.d.). For example, researchers found that there is a declining trend in ASD diagnosis among wealthy White children in CA (Nevison & Parker, 2020).

Here, we present an in-depth analysis of temporal, social, and regional trends in ASD cumulative incidence within each annual birth cohort and diagnosis by 4 and 8 years of age from 1990 to 2018, utilizing comprehensive CA birth records. This represents the longest study duration to date on this topic in a large and diverse population. We are not only examining ASD trends over almost three decades but will also explore sociodemographic factors that may contribute to changes. Furthermore, we examine the influence of neighborhood socioeconomic status (nSES), and region within CA.

## Methods

### Study Population and ASD Ascertainment

Birth records from the CA Department of Public Health (CDPH) for children born between 1990 and 2018 whose mothers resided in CA provided us with sociodemographic factors, including child sex, race/ethnicity, and maternal education. Maternal addresses from the birth registry were used to determine region and neighborhood socioeconomic status (nSES) quintiles derived as the Yost index which is based on principal components analysis of block group level variables from the decennial Census for years 1990–2005 (Yost et al., 2001) and extended using Yang’s index from 2006 to 2018 (Yang et al., 2014). ASD cases were identified from the CA Department of Developmental Services (DDS) from January 1st, 1994 to December 31st, 2022. Children are referred to the DDS for screening or evaluation at 21 regional centers by a parent or professionals, including pediatricians, psychologists, educators, or specialists, who evaluate a child’s developmental progress and identify potential concerns. DDS services are free of charge and available to all Californians independent of income, insurance or immigration status. Upon referral, an assessment team is assigned to gather information on the referred child, including input from parents and educators, medical records, and experts, using observational and standardized assessments for behavior, communication, cognitive abilities, and motor skills. Personalized health and education plans are created and coordinated services are provided encompassing educational support, therapies, vocational training, and access to community resources. ASD diagnosis is recorded on the DDS Client Development Evaluation Report (CDER) based on the Diagnostic and Statistical Manual of Mental Disorders (DSM) IV (up to 2014), and DSM-5 (2015 onwards). ASD was defined as having a full, residual, or suspected syndrome from the DDS records. This database is estimated to contain 75–80% of all children in California with ASD (Croen et al., 2002).

### DDS and Birth Record Linkage

DDS records spanning from 1994 to 2022 (*N* = 196,846) were available to be linked to birth records (*N* = 15,293,518) covering the years 1990 to 2018. This linkage process was facilitated by the National Program of Cancer Registries Registry PlusTM Link Plus software (*Link Plus | Registry Plus | CDC*, 2023). Variables utilized for the probabilistic matching of these two sets of records included the child’s first and last names, child’s date of birth, child’s sex, mother’s first name and maiden name, mother’s date of birth, mother’s race and ethnicity, father’s first and last names, father’s date of birth, and the zip code of the residence. A notable 83% percentage of DDS records were accurately linked to their corresponding birth records. The most frequent cause of incomplete linkage was the absence of parental/child identifiers, coupled with the fact that the individual was not born in California, hence lacking a California birth record. The final sample for this study was comprised of 157,924 ASD cases among15,293,518 total live births.

### Temporal Analysis of ASD Incidence and Age at First Diagnosis

#### Cumulative Incidence of ASD at Age 4 and 8 Years per Birth Year Cohort

Using methods calculating cumulative incidence similar to those outlined in Hertz-Picciotto and Delwiche (2009) as a guide, we calculated the ASD cumulative incidence up to age 4 and 8 years for each birth year cohort. Specifically, for each annual birth cohort spanning from 1990 to 2018, we determined the number of children who developed ASD within 4 years and 8 years of follow-up according to DDS records, i.e. at the time of the child’s first appearance with an ASD diagnosis in CA DDS records before turning 5 or 9 years of age. The number of cases thus identified (numerators) was divided by the total number of children born in CA in the respective birth year (denominator). Henceforth, this measure will be referred to as “ASD cumulative incidence”. Children born outside of the state would not have had a CA birth record and, therefore, were ineligible for this study.

Completed ages 4 and 8 were chosen for two primary reasons, (1) to provide a consistent number of years of follow-up for each birth cohort across the study period, and (2) to investigate whether there were possible variations in case identification before children entered preschool and school-aged children were referred to DDS by educators.

#### Temporal Trend in Mean Age at First Diagnosis

A descriptive statistical analysis was conducted to examine temporal trends in the age at first ASD diagnosis, defined as the year of first appearance in DDS records minus the birth year to provide insights into potential shifts in the age of ASD identification and diagnosis over time. A 4- and 8-years of age follow-up restriction was also implemented to equalize follow-up time across the study period. In the results and discussion sections of this manuscript, the 1991 birth cohort is often used as the first year when describing the diagnosis age trend, as the ASD diagnosis from the available DDS records started in 1994, which artificially assigned a minimum of 4 years of follow-up to all 1990 births who developed ASD.

#### Sociodemographic and Regional Differences in ASD Over Time

We stratified the ASD cumulative incidence by age at first diagnosis (ages 4 and 8) to identify patterns (stable or shifting) as well as disparities or geographical variation. The factors we considered include child sex (male, female), race/ethnicity (White, Hispanic, Black, Asian and Pacific Islander (API)), maternal education (≤ 8th grade, 9th – 12th grade (no diploma), high school graduate or GED, at least some college, graduate degree), nativity (US-born vs. foreign-born), neighborhood socioeconomic status (nSES; 1–5, low to high SES), and region (as defined by the 2020 census regions created by the CA government). These 10 regions were derived from 58 counties, considering factors like “hard-to-count populations, like-mindedness of the counties, capacity of community-based organizations within the counties, and state Census staff workload capabilities” (California, n.d.).

### Ethical Considerations

Approval for this research was obtained from the University of California, Los Angeles, Office of the Human Research Protection Program, and the California Committee for the Protection of Human Subjects. The study was exempted from the requirement for informed consent.

## Results

### Overall ASD Trend for 1990–2018 Birth Cohorts

**Fig. 1:**
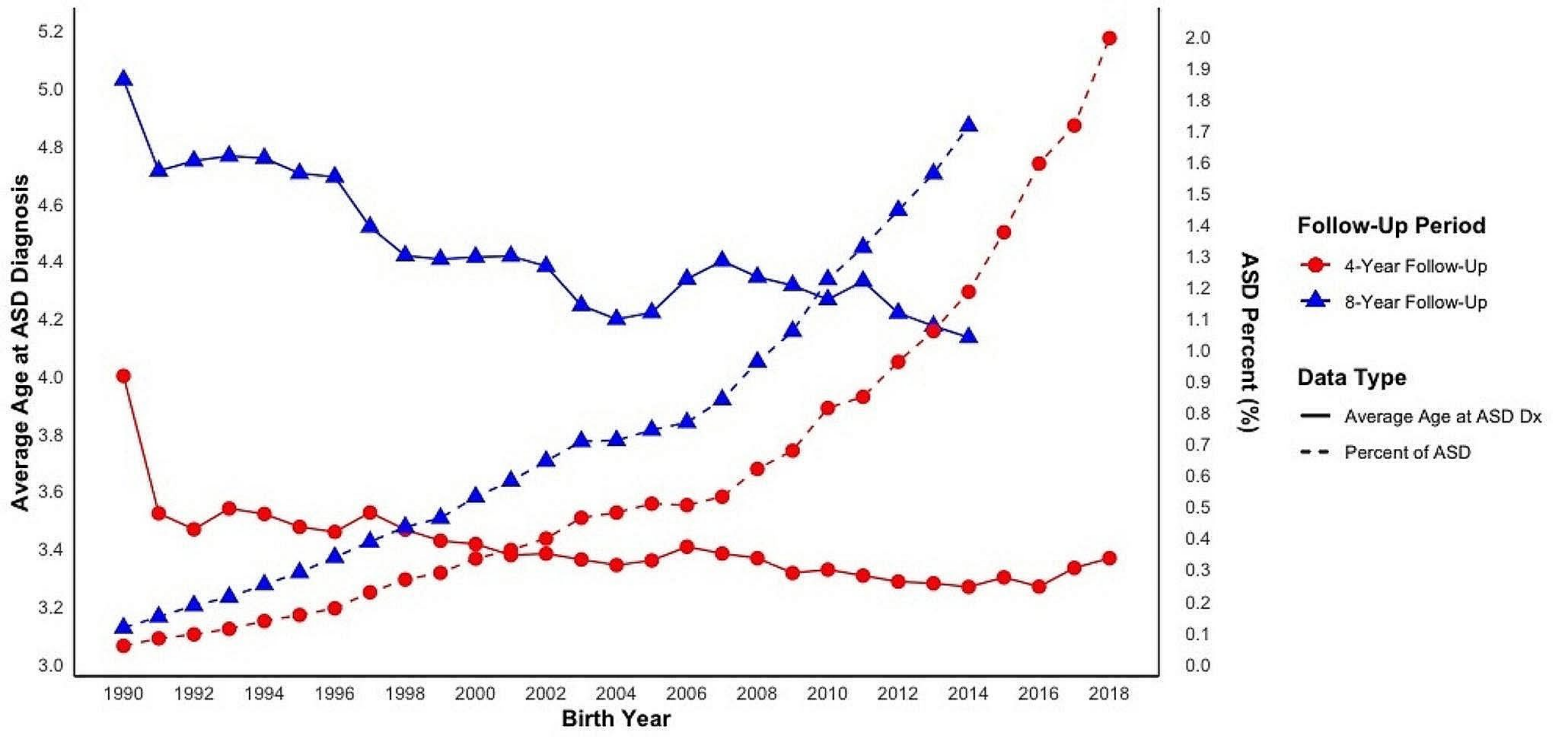
ASD cumulative incidence and average age at diagnosis by birth year cohort. *Notes*: ASD = Autism Spectrum Disorder; Dx = diagnosis; Data presented in table format in Table S1

Over the study period, the ASD cumulative incidence exhibited a distinct upward trend for both maximum diagnosis ages (4 and 8 years) (Fig. 1; Table S1). In 1990, the estimated ASD cumulative incidence by ages 4- and 8-years were 0.06% and 0.11%, respectively. By 2014, these figures had risen to 1.18% and 1.71%. For 2018, the 4-years age at diagnosis limit resulted in an ASD incidence of 1.99%.

Concurrently, there has been a decline in the age of ASD diagnosis in CA over the past three decades. In 1991, the average diagnosis ages within the respective maximum age at diagnosis groups were 3.52 (4 years) and 4.71 years (8 years). By 2014, these averages were reduced to 3.27 and 4.13 years and followed a declining trajectory over time.

### ASD Trend by Sex

While the annual birth cohort cumulative incidence of ASD has been steadily increasing for boys and girls at both 4- and 8-years maximum at diagnosis (Fig. S1; Table S3), the ratio of boys diagnosed with ASD compared to girls shifted from around 5:1 in the early 1990s to closer to 3:1 by 2018 (results not shown). The ASD cumulative incidence for boys diagnosed up to 4 years of age has increased from 0.09% in 1990 to 2.94% in 2018, signifying a substantial rise in the number of ASD cases among boys in CA over nearly three decades. The same pattern is observed for children diagnosed by 8 years of age. Similarly, the percentage of girls diagnosed with ASD before age 4 has risen from 0.02% in 1990 births to 1.00% in 2018. In both boys and girls, there is a general trend of decreasing mean age at diagnosis over the years, with a reasonably similar average age at diagnosis in both sexes (Fig. S2; Table S3).

### ASD Trend by Race/Ethnicity

**Fig. 2:**
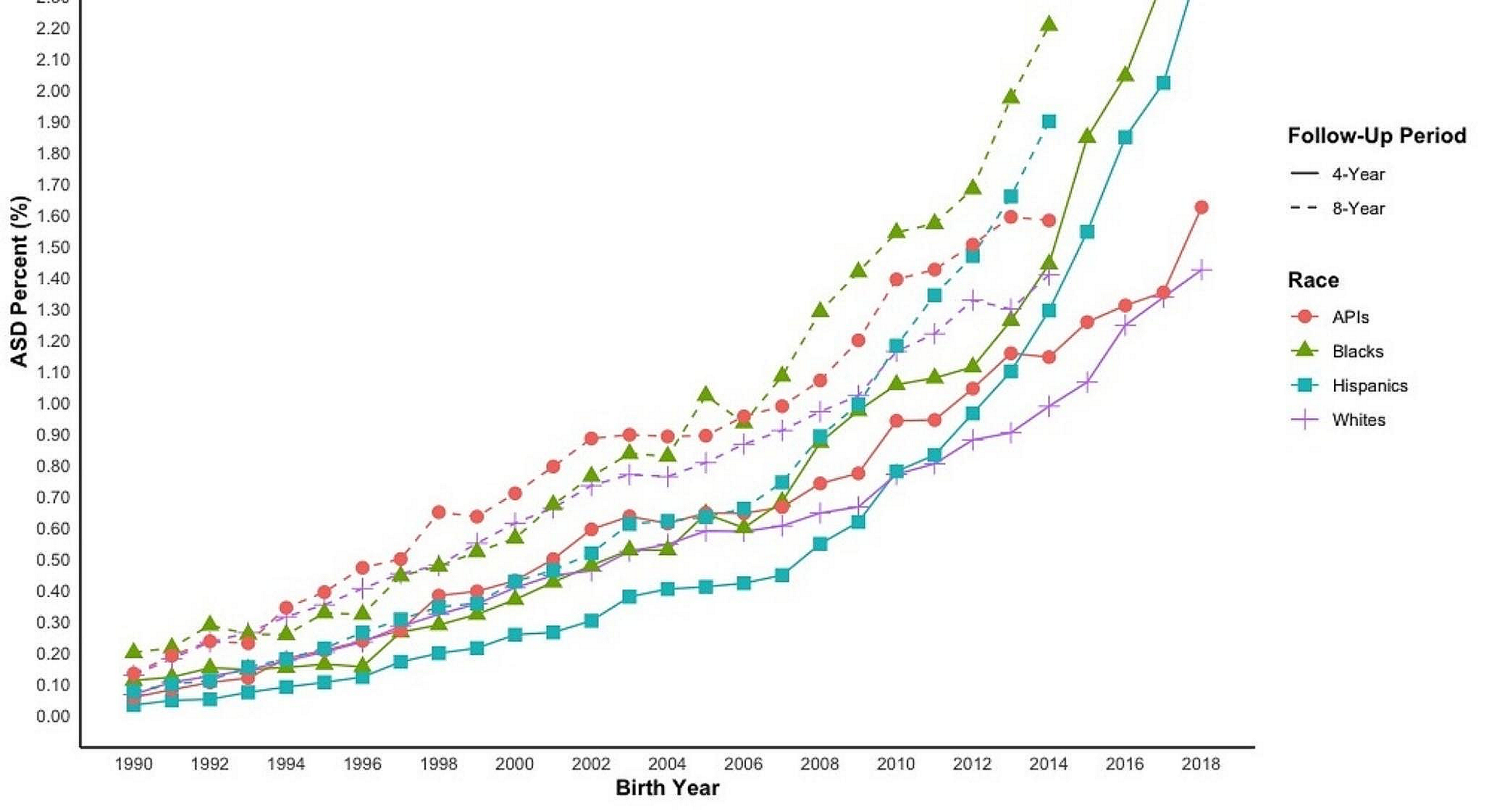
ASD Cumulative Incidence by Birth Year Cohort– Stratified by Race/Ethnicity. *Notes*: ASD = Autism Spectrum Disorder; API = Asians and Pacific Islander; Data presented in table format in Table S2

Similar to the overall trends, race/ethnicity-specific trends in ASD cumulative incidence at 4 and 8 years of age have increased over the past three decades (Fig. 2; Table S2). For example, the annual cumulative incidence of ASD by age 4 among White children steadily increased over the years from 0.07% in 1990 to 1.42% in 2018, for Hispanic children from 0.03% in 1990 to 2.43% in 2018, for Black children from 0.11 to 2.75%, and finally for API children from 0.06 to 1.62%. However, the patterns across different racial/ethnic groups changed. In the 1990s, White children and API children had slightly higher annual cumulative incidences of ASD in both diagnosis age groups, closely followed by Black children, with Hispanic children having the lowest percentage of ASD diagnoses. Black and Hispanic children, however, surpassed White and API children in the 2000s, first Black children in 2004 and then Hispanic children in 2012.

**Fig. 3:**
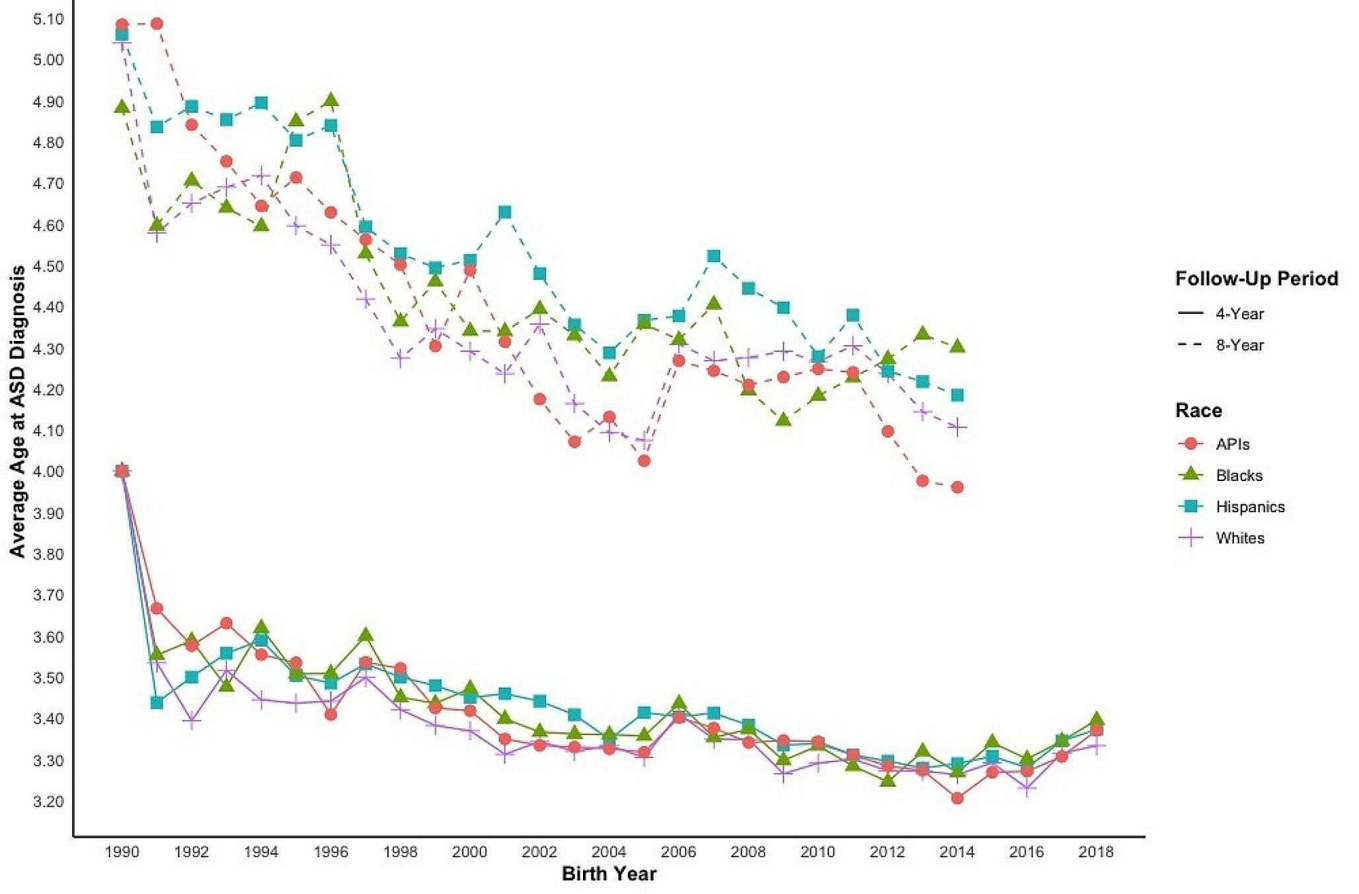
Average Age at Diagnosis by Birth Year Cohort – Stratified by Race/Ethnicity. *Notes*: ASD = Autism Spectrum Disorder; API = Asians and Pacific Islander; Data presented in table format in Table S2

The average age of annual ASD diagnosis consistently decreased across all racial/ethnic groups during the study period, with White and API children consistently having the lowest average annual ASD diagnosis age across the study people (Fig. 3; Table S2).

### ASD Trend by Nativity

Both US-born and foreign-born individuals have had an increasing ASD cumulative incidence by ages 4 and 8, with US-born individuals slightly more likely to be diagnosed with ASD between 1990 and 2010 (Figs.  S3; Table S4). However, since 2010, any apparent difference disappeared. Similarly, US-born individuals were diagnosed at a slightly younger age than foreign-born until around 2010, when this disparity narrowed (Fig. S4; Table S4).

### ASD Trend by Education

In the 1990s until the early 2000s, ASD diagnosis by ages 4 and 8 were highest among children of mothers with a graduate degree education and lowest in mothers with less than or equal to an 8th -grade education (Fig. S5; Table S5). However, by the mid-2000s the upward trajectory among those with a graduate degree had flattened somewhat and children of mothers with lower education levels overtook the highly educated ones. The birth cohort ASD cumulative incidence in the graduate degree group while still increasing remains lower at 1.25%, while, for example, in 2018, children born to 9-12th grade educated mothers had an ASD cumulative incidence at 4 years of 2.45%, closely followed by those with mothers who earned a High School Graduate diploma (2.28%), some college (2.01%), or less than or equal to an 8th grade education (1.81%). These trends were similar for the 8 years at diagnosis group (Fig. S5; Table S5).

### ASD Trend by Neighborhood Socioeconomic Status (nSES)

ASD cumulative incidence also differed by socioeconomic status of a neighborhood, yet the pattern changed from the early 1990s and 2000s to more recent years (Fig. S7; Table S6). In the earlier period, higher ASD cumulative incidence was observed in higher SES neighborhoods while this completely changed around 2010 with low SES neighborhoods having a higher ASD cumulative incidence for both the 4 and 8-year at diagnosis groups. Lower average age at diagnosis was observed in higher SES neighborhoods for virtually every birth cohort in the study period (Fig. S8; Table S6).

### ASD Trend by Region

For the past three decades, there has been a steady increase in ASD cumulative incidence; however, trajectories varied by region (Fig. S9; Table S7). The regional pattern in ASD cumulative incidence persisted across the entire study period with Los Angeles County (0.09%), Orange County (0.06%), and San Francisco Bay Area (0.05%) recording the highest percent of ASD cases by age 4 years of diagnosis from 1990 to the early 2000s but by 2018 the highest incidences were recorded in the Northern San Joaquin Valley (2.95%), a complete reversal from having been one of the two lowest incident counties in the early 1990s (Northern San Joaquin Valley (0.03%) and Southern San Joaquin Valley (0.02%)). Other high ASD cumulative incidence areas for 2018 births include Los Angeles (2.82%) and San Diego-Imperial counties (2.71%). The lowest-ranking incidence regions in 2018 were Orange County (0.89%), Inland Empire (1.17%), and the San Francisco Bay Area (1.25%). The patterns for the 8-years at diagnosis group were very similar.

The average age at diagnosis showed greater variation across time by region and several patterns emerged (Fig. S10; Table S7). While having some of the lowest ASD cumulative incidence at 4 and 8 years across in the later part of the study period, Orange County typically also had the youngest average age at diagnosis. In contrast, Southern San Joaquin Valley had not only one of the highest ASD percentages in 2018, but throughout the study period also had one of the highest average ages at diagnosis. Thus, higher ASD cumulative incidence did not seem to correlate with lower average age at diagnosis in the highest and lowest ASD cumulative incidence regions.

## Discussion

In the present study, we have conducted a comprehensive examination of the trends in annual ASD cumulative incidence between 1990 and 2018, based on 4 and 8 years of maximum age at diagnosis. We document shifts in annual ASD cumulative incidence by birth cohort over time that differ by key sociodemographic factors such as race/ethnicity, maternal educational attainment, nSES, and also exhibit regional variations. The CA data enable us to explore ASD disparities and inequalities and to further contribute to the dialogue on ASD etiology and treatment resources.

Our study findings replicate previous reports from earlier years including those conducted by the CDC, which have consistently reported an increasing prevalence of ASD over recent decades (Baio et al., 2018; C.D.C., 2009; Hertz-Picciotto & Delwiche, 2009; Maenner, 2021, 2023). The rising trend has been attributed to a combination of factors, including increased awareness, improved diagnostic tools, changes in diagnostic criteria, and a genuine increase in ASD (Hansen et al., 2015). The decline in the age of ASD diagnosis in CA over the past thirty years also suggests an evolving diagnostic landscape, where clinicians, educators, and parents are becoming more adept at recognizing early signs of ASD. Such early identification is critical, as it allows for earlier intervention that may be more effective in improving developmental outcomes. The American Academy of Pediatrics recommends screening for ASD during regular well-child doctor visits at 18 and 24 months (American Academy of Pediatrics, 2023). Major strides in both diagnoses and access to treatment over the past three decades in CA may also explain the decreasing boy-to-girl ASD ratio, from around 5:1 in the early 1990s to closer to 3:1 more recently, suggesting improved access interventions may be having success. Girls have long been thought to have been underdiagnosed or diagnosed much later (McCrossin, 2022).

Some have argued that factors like younger diagnosis age and changes in diagnostic criteria cannot explain the increases completely (Hertz-Picciotto & Delwiche, 2009). This conclusion was made based on data up to 2006; however, our results suggest around this time, a major reversal in ASD cumulative incidence took place by race/ethnicity in terms of which groups were at highest risk. Our data show an upward trend in annual ASD cumulative incidence across all racial and ethnic groups. Nevertheless, specific demographic patterns reveal disparities that persist. In the 1990s, White and API children had marginally higher cumulative incidences than Black and Hispanic children; however,by 2004, Black children surpassed White and API children, and by 2012, Hispanic children followed as also reported by others (Aylward et al., 2021; Maenner, 2023; Nevison & Parker, 2020; Pedersen et al., 2012). This might reflect better access to agencies that diagnose ASD and/or heightened awareness of ASD in minority communities, which was not as prevalent before the mid-2000s. This reversal may be a shift towards a ‘true’ risk that had been previously under-reported/diagnosed.

Additionally, evolving diagnostic criteria or screening techniques may have introduced these changes. Notably, between 1990 and 2018, clinicians utilized four versions of the DSM-IV (1994), DSM-IV-TR (2000), and DSM-5 (2013) (*DSM History*, n.d.). The transition to DSM-5, for instance, marked substantial diagnostic alterations (Tsai, 2012). Where once three separate conditions existed, they merged into a unified “Autism Spectrum Disorder” category. DSM-IV operated on three domains, whereas DSM-5 consolidated these into two, embracing a broader view of the disorder’s spectrum nature. Such expansion of criteria may generate ASD diagnoses in traditionally underdiagnosed segments, including girls and minorities. It is difficult to conclude a direct link between these changes and the observed shift in ASD cumulative incidence as the changes do not line up particularly well. Yet, it is plausible that the influence of changing diagnosis criteria takes time to materialize, or that factors that led to diagnostic changes were already influencing awareness and diagnoses prior to being standardized in text.

Furthermore, the Children’s Health Insurance Program (CHIP) in the late 1990s (Adams et al., 2019) and its subsequent Medicaid expansion and the Affordable Care Act in 2010 (McBain et al., 2022), all may have increased access to diagnosis and care for a more diverse clientele. In fact, Rea et al. (2019) and Augustyn et al. (2020) found little differences in ASD screening, referral, and wait time to care between White and non-White children (Augustyn et al., 2020; Rea et al., 2019). Yet, even though Black and Hispanic children are outpacing White and API children now, the latter still have slightly earlier diagnoses ages suggesting enduring disparities, and the higher proportions may suggest disparities in environmental exposures and/or vulnerabilities.

Similarly, maternal education, a proxy for family socio-economic status and income, as well as access to care and information about ASD seems to have contributed to differences in annual ASD cumulative incidence. This social disparity is further highlighted by neighborhood SES trends in ASD, for which we saw a similar transformation over time with higher SES neighborhoods initially exhibiting greater annual ASD cumulative incidence consistent with the literature describing higher diagnosis rates in families with the means to pursue professional evaluations (Durkin et al., 2010). However, by 2005–2006, there was a reversal such that low SES neighborhoods took the top spot, even though there was still a lower average age at diagnosis in higher nSES.

Nevison and Parker (2020) have suggested that there might be a stabilization or even a decrease in ASD rates among the affluent White populations, which could be attributed to these individuals opting out of developmental disability services in preference for private care, or due to other changes that may have reduced ASD risk in this group (Nevison & Parker, 2020). Our observations align with those of Nevison and Parker (2020), showing a leveling off in ASD cumulative incidence among high nSES White children after 2013. Additionally, high nSES API populations are exhibiting a similar trend (Fig. S11). In 2018, the lowest annual ASD cumulative incidence was observed in high socioeconomic status neighborhoods for all racial groups again suggesting that studies of environmental exposures and vulnerabilities among minorities living in low nSES neighborhoods are urgently needed (Fig. S11.).

In the early 1990s to 2000s, metropolitan regions with denser healthcare infrastructures, such as Los Angeles, San Francisco, and Orange County, saw higher cumulative incidences of ASD diagnoses. Contrastingly, economically challenged Central Valley areas, like the Northern and Southern San Joaquin Valleys, reported lower figures. This trend shifted in the late 2000s and 2010s, as these areas started reporting higher ASD cumulative incidences. Although improved rural healthcare access may also account for some of this change, the persistently delayed diagnosis age in these regions compared to other regions suggests lingering disparities. Moreover, the Central Valley areas are known for high pesticide exposures and air pollution—both factors that have been linked to ASD (Becerra et al., 2013; Bradman et al., 1997; Cisneros et al., 2017; Ehrenstein et al., 2019; Yan et al., 2021).

The study’s reliance on DDS data for determining ASD cases is a limitation and a strength, as this statewide-wide and high-quality service provides reliable ASD counts but still underestimates the total number as ~ 75–80% of all ASD in CA enroll in these services (Croen et al., 2002). Furthermore, the DDS may skew towards more severe ASD in some regions and neighborhoods and thus bias regional and other comparisons. Additionally, our findings are necessarily influenced by variations in diagnostic practices over these three decades, which may have affected the consistency of ASD recognition and reporting and may have been driven by particular demographics, such as affluence or race/ethnicity. Using all births per year as the denominator in our study, we potentially include children who have died or moved out of the state, artificially increasing the denominator and possibly underestimating the ASD cumulative incidence. Finally, while our sample size is large, some racial/ethnic categories such as API still cannot be broken down further into subgroups of interest due to small cell counts, particularly in the earliest years of the study.

## Conclusions

This research highlights how the patterns of cumulative incidence of ASD have evolved over time within different areas and populations in CA. In the 1990s, the highest cumulative incidences were found among communities with higher socioeconomic status, more educated mothers, and predominantly White and Asian/Pacific Islander (API) populations. Recently, the trend has shifted, showing the highest cumulative incidences in areas with lower neighborhood SES, Hispanic and Black populations, and among mothers with lower educational attainment. In addition, although wealthier regions were initially the ones to report more cases, there has been a notable increase in cases in places like the Central Valley. Despite advancements, there remains a persistent gap in the timing of ASD diagnoses among various socioeconomic groups, potentially influenced by factors such as race and maternal education levels. This situation calls for targeted interventions and research tailored for different regions and communities to further reduce disparities.

## Electronic Supplementary Material

Below is the link to the electronic supplementary material.

Supplementary Material 1
