# Supplementary material for: Trends in the Prevalence of Autism Spectrum Disorder in California: Disparities by Sociodemographic Factors and Region Between 1990–2018
Source: J Autism Dev Disord. 2024 May 3;55(7):2503–11. doi: 10.1007/s10803-024-06371-w (PMC12167244; doi:10.1007/s10803-024-06371-w)
Supplement: Supplementary file 1 — Supplementary Material 1 [file 10803_2024_6371_MOESM1_ESM.docx]

**Title**: Trends in the Prevalence of Autism Spectrum Disorder in California: Disparities by Socioeconomic Factors and Region Between 1990-2018

**SUPPLEMENTAL MATERIALS**

***ASD = Autism Spectrum Disorder**

*** nSES = Neighborhood Socioeconomic Status (1 – “Low SES” to 5 – “High SES”)**

**FIGURES:**

**Figure S1.**


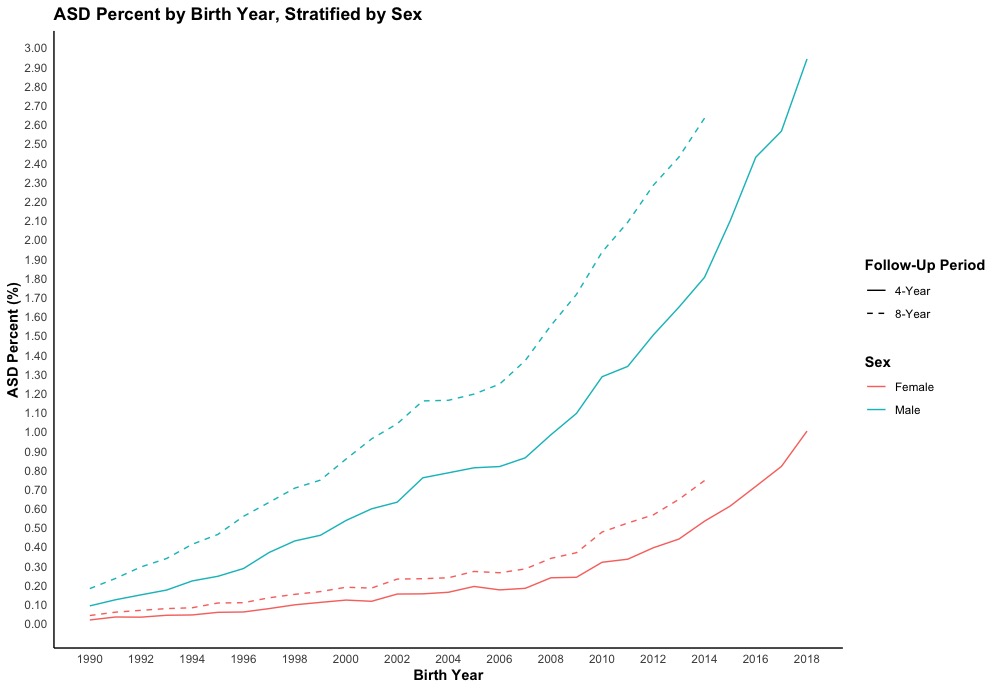


**Notes**: ASD = Autism Spectrum Disorder; Data presented in table format in Table S3.

**Figure S2.**


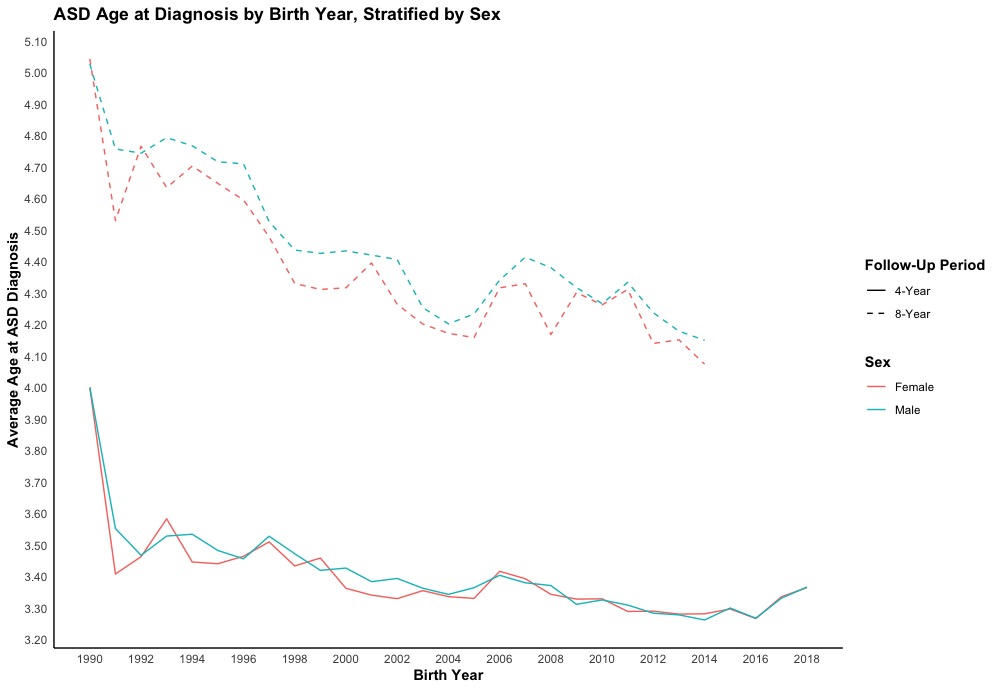


**Notes**: ASD = Autism Spectrum Disorder; Data presented in table format in Table S3.

**Figure S3.**


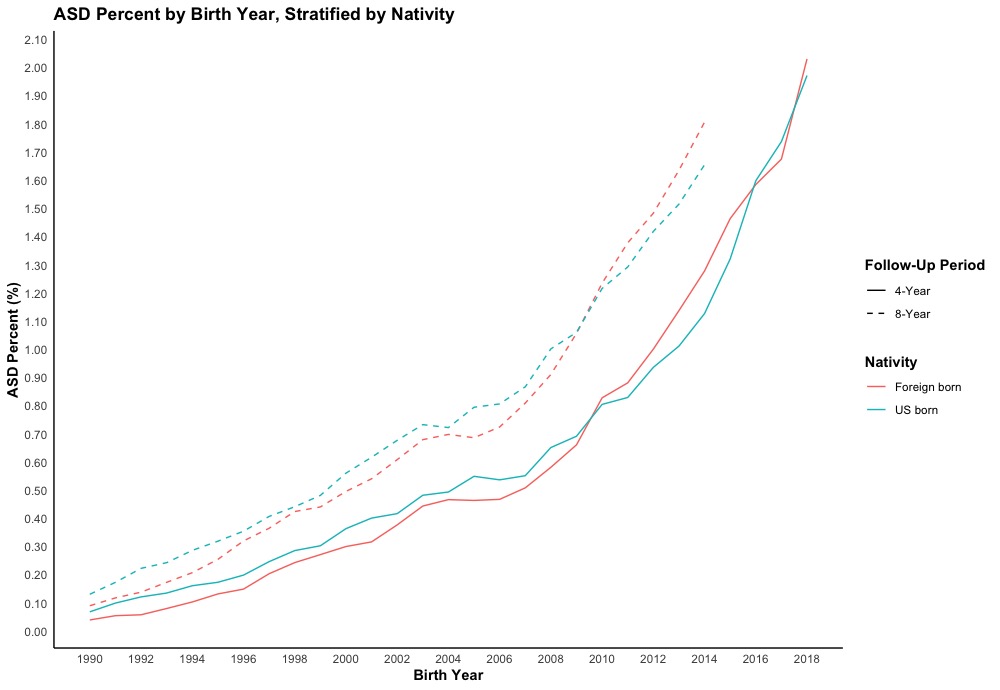


**Notes**: ASD = Autism Spectrum Disorder; Data presented in table format in Table S4.

**Figure S4.**


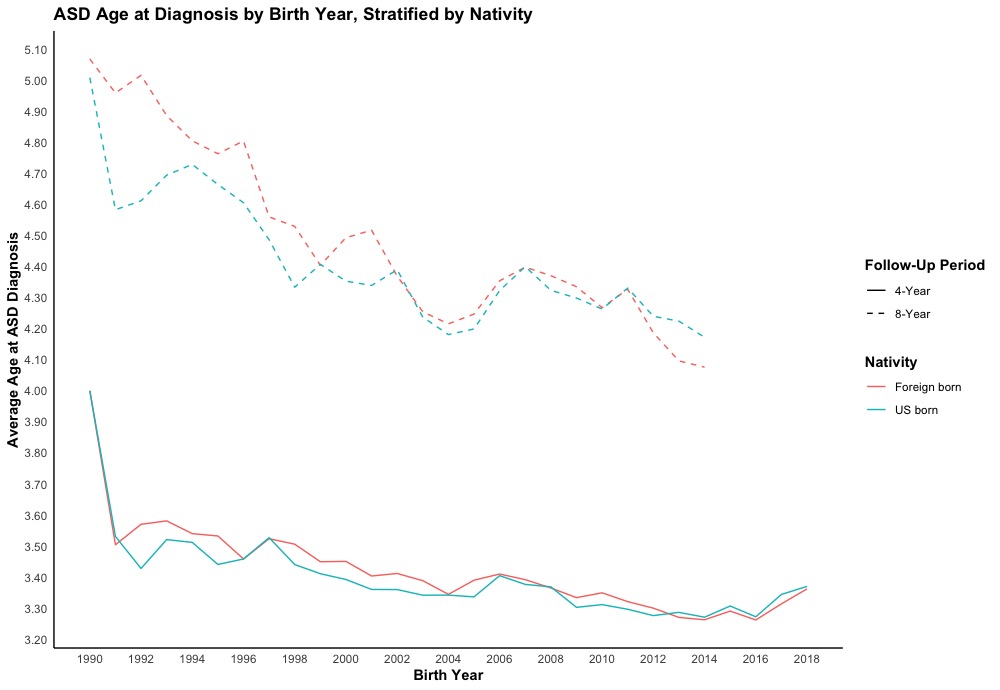


**Notes**: ASD = Autism Spectrum Disorder; Data presented in table format in Table S4.

**Figure S5.**


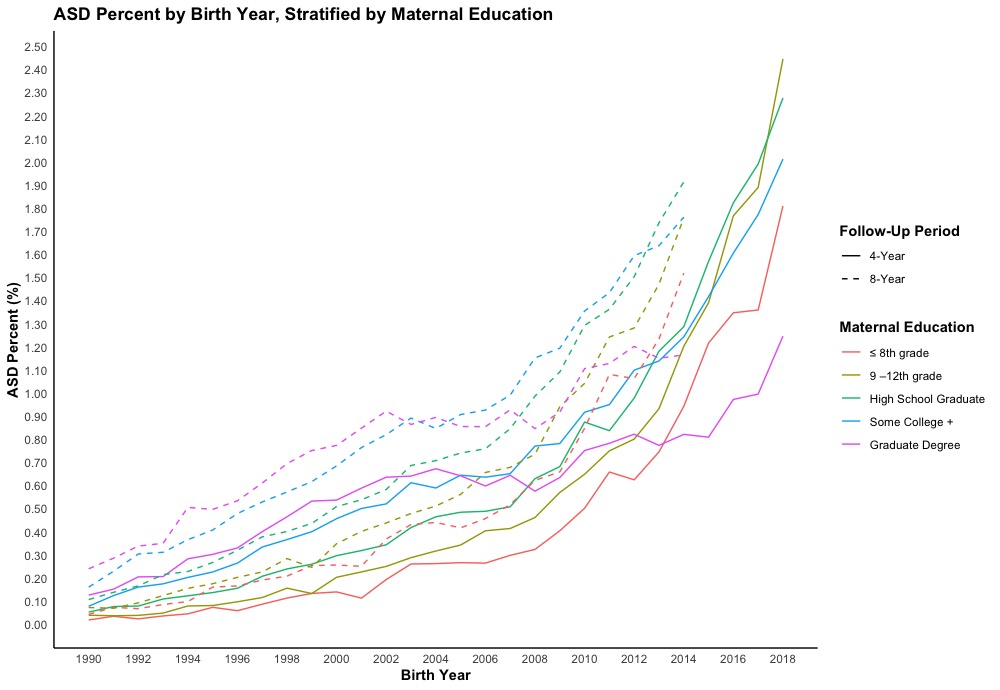


**Notes**: ASD = Autism Spectrum Disorder; Data presented in table format in Table S5.

**Figure S6.**


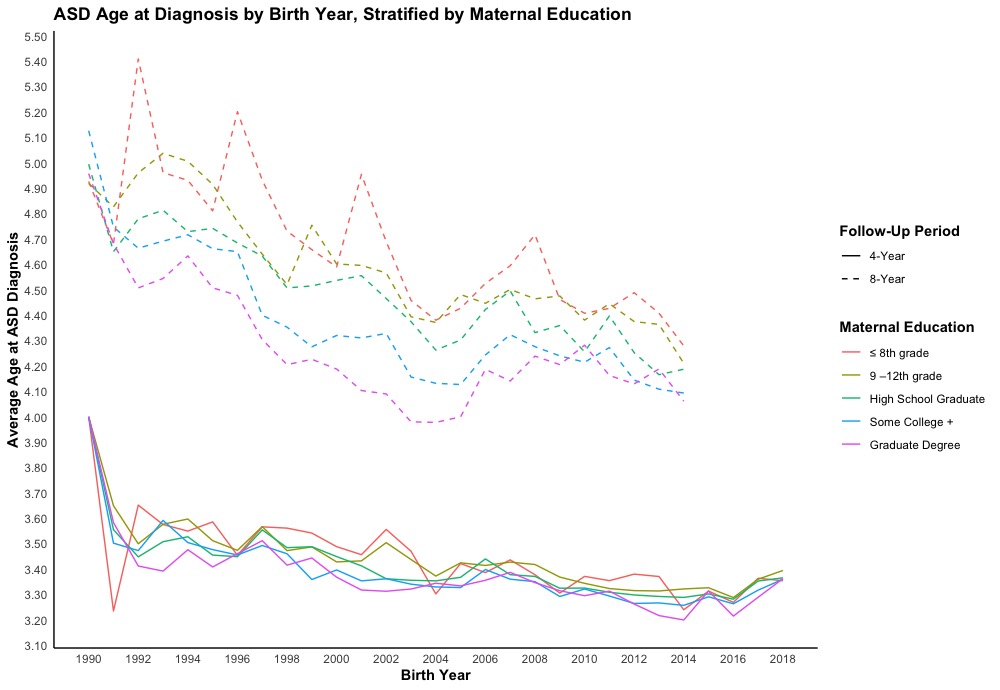


**Notes**: ASD = Autism Spectrum Disorder; Data presented in table format in Table S5.

**Figure S7.**

**
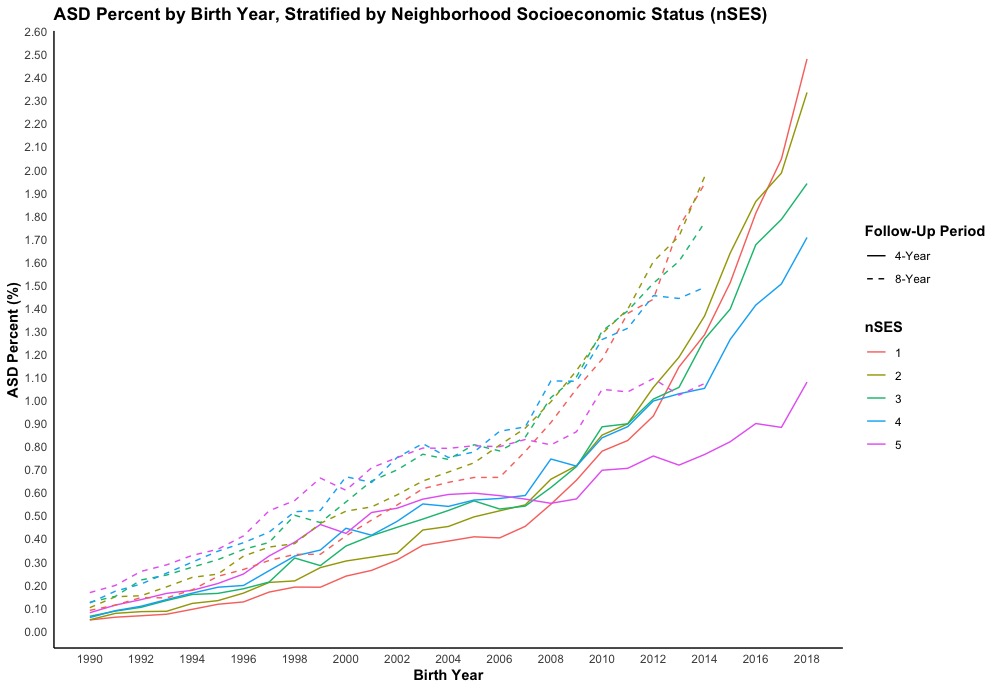
**

**Notes**: ASD = Autism Spectrum Disorder; Data presented in table format in Table S6.

**Figure S8.**

**
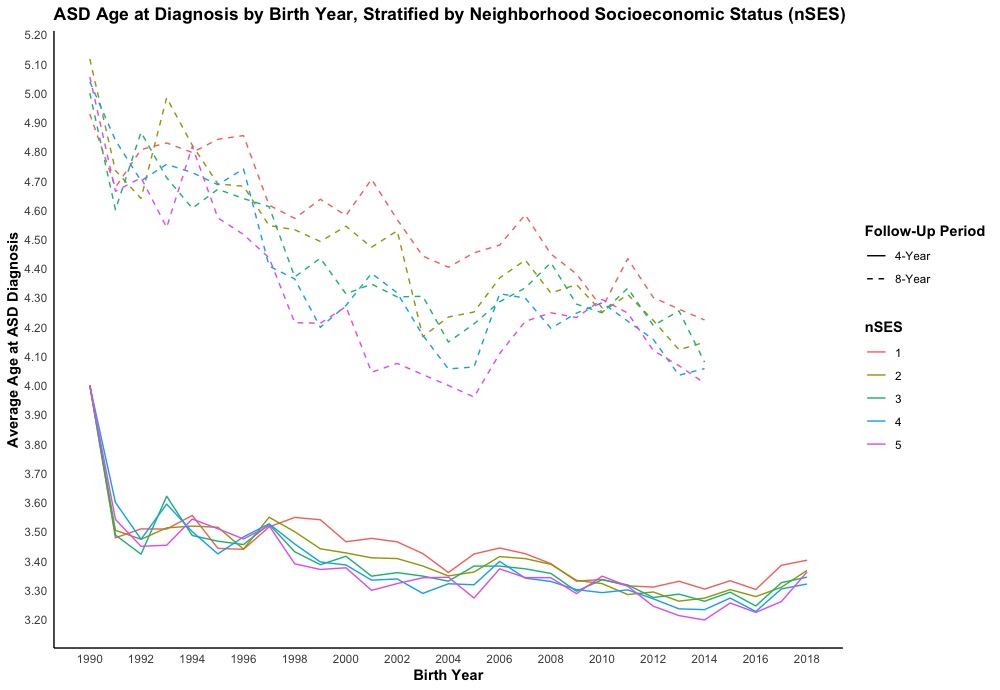
**

**Notes**: ASD = Autism Spectrum Disorder; Data presented in table format in Table S6.

**Figure S9.**


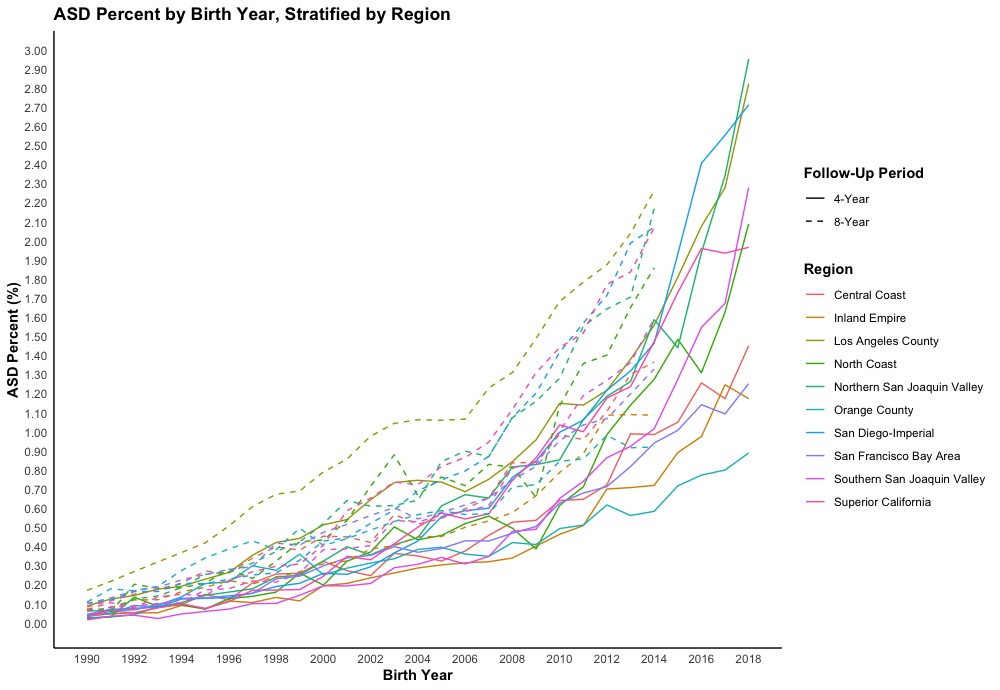


**Notes**: ASD = Autism Spectrum Disorder; Data presented in table format in Table S7.

**Figure S10.**


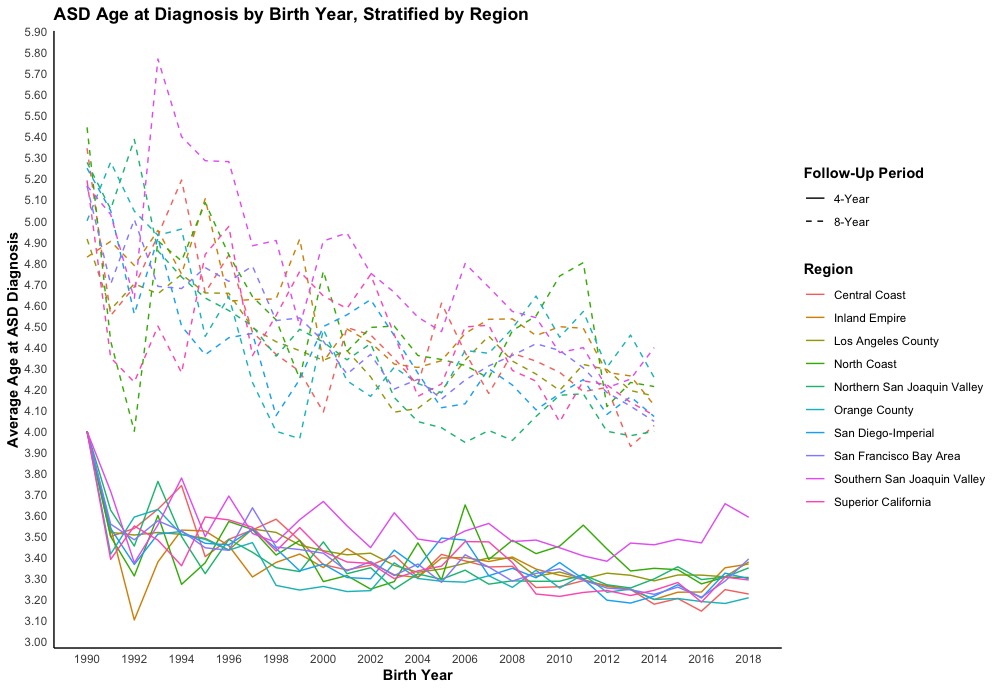


**Notes**: ASD = Autism Spectrum Disorder; Data presented in table format in Table S7.

**Figure S11.**

**
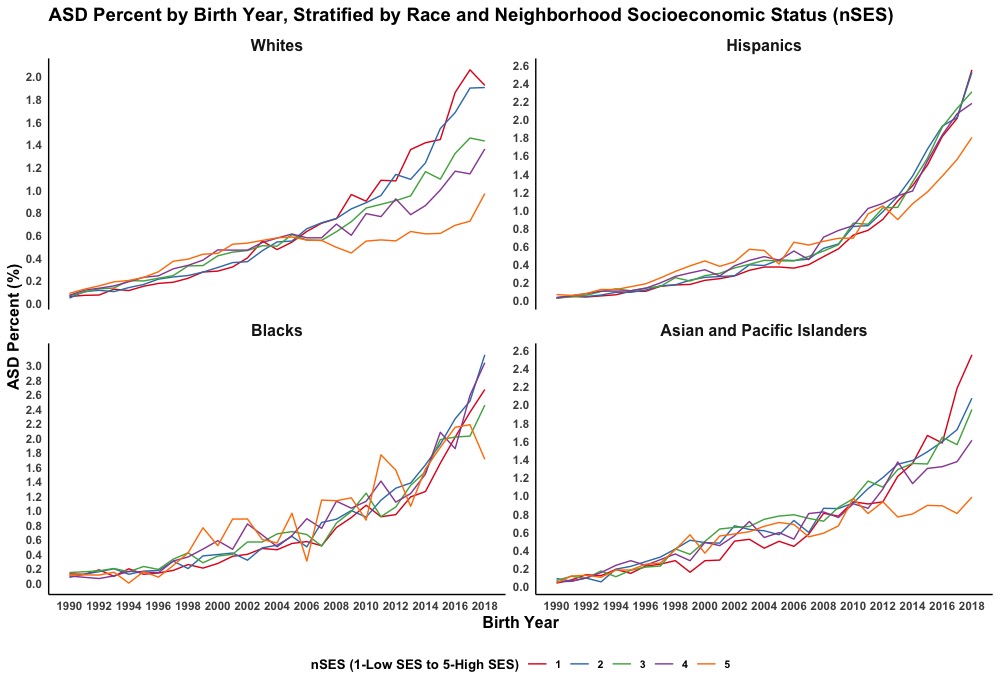
**

**TABLES:**

| **Table S1.** ASD Cumulative Incidence and Average Age at Diagnosis by Birth Year Cohort | | | | | | | | | | |  |
| --- | --- | --- | --- | --- | --- | --- | --- | --- | --- | --- | --- |
|  | **ASD by 4 Years** | | | | | **ASD by 8 Years** | | | | |  |
|  | **Cumulative Incidence** | | | **Average Age at Diagnosis** | | **Cumulative Incidence** | | | **Average Age at Diagnosis** | |  |
| **Birth Year** | **ASD Cases** | **Total Births** | **Percent** | **Mean (Yrs)** | **SD (Yrs)** | **ASD Cases** | **Total Births** | **Percent** | **Mean (Yrs)** | **SD (Yrs)** |  |
| 2018 | 9,051 | 454,144 | 1.99% | 3.37 | 0.49 | --- | --- | --- | --- | --- |  |
| 2017 | 8,052 | 469,674 | 1.71% | 3.33 | 0.48 | --- | --- | --- | --- | --- |  |
| 2016 | 7,754 | 486,661 | 1.59% | 3.27 | 0.45 | --- | --- | --- | --- | --- |  |
| 2015 | 6,728 | 489,533 | 1.37% | 3.30 | 0.47 | --- | --- | --- | --- | --- |  |
| 2014 | 5,929 | 500,465 | 1.18% | 3.27 | 0.46 | 8,572 | 500,465 | 1.71% | 4.13 | 1.49 |  |
| 2013 | 5,215 | 492,235 | 1.06% | 3.28 | 0.46 | 7,684 | 492,235 | 1.56% | 4.17 | 1.48 |  |
| 2012 | 4,820 | 501,249 | 0.96% | 3.29 | 0.47 | 7,238 | 501,249 | 1.44% | 4.22 | 1.50 |  |
| 2011 | 4,255 | 500,650 | 0.85% | 3.31 | 0.48 | 6,641 | 500,650 | 1.33% | 4.33 | 1.56 |  |
| 2010 | 4,152 | 509,898 | 0.81% | 3.33 | 0.50 | 6,240 | 509,898 | 1.22% | 4.27 | 1.52 |  |
| 2009 | 3,563 | 525,061 | 0.68% | 3.31 | 0.48 | 5,559 | 525,061 | 1.06% | 4.31 | 1.53 |  |
| 2008 | 3,412 | 550,100 | 0.62% | 3.37 | 0.48 | 5,287 | 550,100 | 0.96% | 4.34 | 1.52 |  |
| 2007 | 3,001 | 564,354 | 0.53% | 3.38 | 0.49 | 4,743 | 564,354 | 0.84% | 4.40 | 1.54 |  |
| 2006 | 2,831 | 560,788 | 0.50% | 3.41 | 0.50 | 4,309 | 560,788 | 0.77% | 4.34 | 1.50 |  |
| 2005 | 2,787 | 547,002 | 0.51% | 3.36 | 0.51 | 4,070 | 547,002 | 0.74% | 4.22 | 1.47 |  |
| 2004 | 2,617 | 543,986 | 0.48% | 3.34 | 0.50 | 3,868 | 543,986 | 0.71% | 4.20 | 1.43 |  |
| 2003 | 2,507 | 539,782 | 0.46% | 3.36 | 0.50 | 3,822 | 539,782 | 0.71% | 4.24 | 1.42 |  |
| 2002 | 2,102 | 527,621 | 0.40% | 3.38 | 0.51 | 3,404 | 527,621 | 0.65% | 4.38 | 1.47 |  |
| 2001 | 1,904 | 525,920 | 0.36% | 3.38 | 0.51 | 3,059 | 525,920 | 0.58% | 4.42 | 1.54 |  |
| 2000 | 1,776 | 530,655 | 0.33% | 3.42 | 0.51 | 2,818 | 530,655 | 0.53% | 4.41 | 1.52 |  |
| 1999 | 1,494 | 516,698 | 0.29% | 3.43 | 0.51 | 2,395 | 516,698 | 0.46% | 4.41 | 1.49 |  |
| 1998 | 1,396 | 521,228 | 0.27% | 3.47 | 0.51 | 2,268 | 521,228 | 0.44% | 4.42 | 1.43 |  |
| 1997 | 1,177 | 518,051 | 0.23% | 3.53 | 0.51 | 2,010 | 518,051 | 0.39% | 4.52 | 1.41 |  |
| 1996 | 924 | 524,498 | 0.18% | 3.46 | 0.52 | 1,777 | 524,498 | 0.34% | 4.69 | 1.51 |  |
| 1995 | 823 | 531,579 | 0.15% | 3.48 | 0.52 | 1,543 | 531,579 | 0.29% | 4.70 | 1.54 |  |
| 1994 | 746 | 550,391 | 0.14% | 3.52 | 0.52 | 1,384 | 550,391 | 0.25% | 4.76 | 1.59 |  |
| 1993 | 627 | 566,974 | 0.11% | 3.54 | 0.54 | 1,200 | 566,974 | 0.21% | 4.76 | 1.53 |  |
| 1992 | 542 | 582,355 | 0.09% | 3.47 | 0.58 | 1,077 | 582,355 | 0.18% | 4.75 | 1.55 |  |
| 1991 | 469 | 585,031 | 0.08% | 3.52 | 0.50 | 873 | 585,031 | 0.15% | 4.71 | 1.51 |  |
| 1990 | 326 | 576,935 | 0.06% | 4.00 | 0.00 | 657 | 576,935 | 0.11% | 5.03 | 1.28 |  |
| Notes: SD = Standard Deviation; ASD = Autism Spectrum Disorder; Yrs = Years | | | | | | | | | | |  |
|  |  |  |  |  |  |  |  |  |  |  |  |

| **Table S2.** ASD Cumulative Incidence and Average Age at Diagnosis by Birth Year Cohort – Stratified by Race/Ethnicity | | | | | | | | | | |
| --- | --- | --- | --- | --- | --- | --- | --- | --- | --- | --- |
| **White** | | | | | | | | | | |
|  | **ASD by 4 Years** | | | | | **ASD by 8 Years** | | | | |
|  | **Cumulative Incidence** | | | **Average Age at Diagnosis** | | **Cumulative Incidence** | | | **Average Age at Diagnosis** | |
| **Birth Year** | **ASD Cases** | **Total Births** | **Percent** | **Mean (Yrs)** | **SD (Yrs)** | **ASD Cases** | **Total Births** | **Percent** | **Mean (Yrs)** | **SD (Yrs)** |
| 2018 | 1,806 | 126,764 | 1.42% | 3.33 | 0.49 | --- | --- | --- | --- | --- |
| 2017 | 1,763 | 131,850 | 1.34% | 3.32 | 0.48 | --- | --- | --- | --- | --- |
| 2016 | 1,708 | 136,873 | 1.25% | 3.23 | 0.44 | --- | --- | --- | --- | --- |
| 2015 | 1,499 | 140,658 | 1.07% | 3.29 | 0.46 | --- | --- | --- | --- | --- |
| 2014 | 1,416 | 143,221 | 0.99% | 3.26 | 0.47 | 2,017 | 143,221 | 1.41% | 4.11 | 1.48 |
| 2013 | 1,288 | 142,474 | 0.90% | 3.27 | 0.46 | 1,851 | 142,474 | 1.30% | 4.14 | 1.50 |
| 2012 | 1,248 | 141,605 | 0.88% | 3.27 | 0.48 | 1,880 | 141,605 | 1.33% | 4.24 | 1.54 |
| 2011 | 1,157 | 143,743 | 0.80% | 3.30 | 0.47 | 1,754 | 143,743 | 1.22% | 4.30 | 1.59 |
| 2010 | 1,120 | 145,213 | 0.77% | 3.29 | 0.50 | 1,690 | 145,213 | 1.16% | 4.27 | 1.56 |
| 2009 | 965 | 144,735 | 0.67% | 3.27 | 0.46 | 1,480 | 144,735 | 1.02% | 4.29 | 1.59 |
| 2008 | 969 | 149,646 | 0.65% | 3.35 | 0.48 | 1,454 | 149,646 | 0.97% | 4.28 | 1.50 |
| 2007 | 933 | 153,886 | 0.61% | 3.35 | 0.48 | 1,402 | 153,886 | 0.91% | 4.27 | 1.49 |
| 2006 | 925 | 157,223 | 0.59% | 3.41 | 0.49 | 1,364 | 157,223 | 0.87% | 4.31 | 1.51 |
| 2005 | 934 | 158,368 | 0.59% | 3.31 | 0.50 | 1,280 | 158,368 | 0.81% | 4.08 | 1.46 |
| 2004 | 884 | 161,574 | 0.55% | 3.33 | 0.50 | 1,234 | 161,574 | 0.76% | 4.09 | 1.40 |
| 2003 | 869 | 165,455 | 0.53% | 3.32 | 0.50 | 1,274 | 165,455 | 0.77% | 4.16 | 1.44 |
| 2002 | 757 | 163,637 | 0.46% | 3.34 | 0.50 | 1,203 | 163,637 | 0.74% | 4.36 | 1.51 |
| 2001 | 745 | 166,219 | 0.45% | 3.31 | 0.51 | 1,103 | 166,219 | 0.66% | 4.24 | 1.52 |
| 2000 | 698 | 170,850 | 0.41% | 3.37 | 0.51 | 1,051 | 170,850 | 0.62% | 4.29 | 1.51 |
| 1999 | 612 | 171,229 | 0.36% | 3.38 | 0.50 | 943 | 171,229 | 0.55% | 4.35 | 1.53 |
| 1998 | 570 | 176,307 | 0.32% | 3.42 | 0.51 | 847 | 176,307 | 0.48% | 4.27 | 1.43 |
| 1997 | 497 | 174,456 | 0.28% | 3.50 | 0.51 | 792 | 174,456 | 0.45% | 4.42 | 1.42 |
| 1996 | 421 | 178,836 | 0.24% | 3.44 | 0.52 | 726 | 178,836 | 0.41% | 4.55 | 1.52 |
| 1995 | 380 | 187,195 | 0.20% | 3.44 | 0.52 | 663 | 187,195 | 0.35% | 4.60 | 1.55 |
| 1994 | 344 | 196,194 | 0.18% | 3.44 | 0.53 | 620 | 196,194 | 0.32% | 4.72 | 1.67 |
| 1993 | 298 | 204,061 | 0.15% | 3.52 | 0.57 | 541 | 204,061 | 0.27% | 4.69 | 1.55 |
| 1992 | 274 | 218,136 | 0.13% | 3.39 | 0.61 | 512 | 218,136 | 0.23% | 4.65 | 1.61 |
| 1991 | 245 | 229,042 | 0.11% | 3.53 | 0.50 | 417 | 229,042 | 0.18% | 4.58 | 1.47 |
| 1990 | 160 | 237,086 | 0.07% | 4.00 | 0.00 | 305 | 237,086 | 0.13% | 5.04 | 1.35 |

| (Table S2. continued) | | | | | | | | | | |
| --- | --- | --- | --- | --- | --- | --- | --- | --- | --- | --- |
| **Hispanic** | | | | | | | | | | |
|  | **ASD by 4 Years** | | | | | **ASD by 8 Years** | | | | |
|  | **Cumulative Incidence** | | | **Average Age at Diagnosis** | | **Cumulative Incidence** | | | **Average Age at Diagnosis** | |
| **Birth Year** | **ASD Cases** | **Total Births** | **Percent** | **Mean (Yrs)** | **SD (Yrs)** | **ASD Cases** | **Total Births** | **Percent** | **Mean (Yrs)** | **SD (Yrs)** |
| 2018 | 5,112 | 210,687 | 2.43% | 3.37 | 0.49 | --- | --- | --- | --- | --- |
| 2017 | 4,424 | 218,812 | 2.02% | 3.35 | 0.48 | --- | --- | --- | --- | --- |
| 2016 | 4,207 | 227,596 | 1.85% | 3.28 | 0.46 | --- | --- | --- | --- | --- |
| 2015 | 3,602 | 232,976 | 1.55% | 3.31 | 0.47 | --- | --- | --- | --- | --- |
| 2014 | 3,058 | 236,193 | 1.29% | 3.29 | 0.47 | 4,486 | 236,193 | 1.90% | 4.18 | 1.51 |
| 2013 | 2,609 | 237,203 | 1.10% | 3.28 | 0.46 | 3,936 | 237,203 | 1.66% | 4.22 | 1.49 |
| 2012 | 2,351 | 243,431 | 0.97% | 3.30 | 0.47 | 3,576 | 243,431 | 1.47% | 4.24 | 1.49 |
| 2011 | 2,073 | 248,986 | 0.83% | 3.31 | 0.47 | 3,345 | 248,986 | 1.34% | 4.38 | 1.56 |
| 2010 | 2,008 | 257,263 | 0.78% | 3.34 | 0.50 | 3,041 | 257,263 | 1.18% | 4.28 | 1.51 |
| 2009 | 1,666 | 269,343 | 0.62% | 3.33 | 0.49 | 2,680 | 269,343 | 1.00% | 4.40 | 1.56 |
| 2008 | 1,576 | 287,158 | 0.55% | 3.38 | 0.49 | 2,563 | 287,158 | 0.89% | 4.44 | 1.55 |
| 2007 | 1,331 | 296,610 | 0.45% | 3.41 | 0.49 | 2,211 | 296,610 | 0.75% | 4.52 | 1.57 |
| 2006 | 1,238 | 292,556 | 0.42% | 3.40 | 0.49 | 1,935 | 292,556 | 0.66% | 4.38 | 1.50 |
| 2005 | 1,160 | 281,973 | 0.41% | 3.41 | 0.52 | 1,790 | 281,973 | 0.63% | 4.37 | 1.49 |
| 2004 | 1,112 | 274,944 | 0.40% | 3.35 | 0.49 | 1,710 | 274,944 | 0.62% | 4.29 | 1.48 |
| 2003 | 1,023 | 269,284 | 0.38% | 3.41 | 0.51 | 1,650 | 269,284 | 0.61% | 4.36 | 1.42 |
| 2002 | 795 | 262,298 | 0.30% | 3.44 | 0.51 | 1,361 | 262,298 | 0.52% | 4.48 | 1.44 |
| 2001 | 692 | 260,173 | 0.27% | 3.46 | 0.51 | 1,208 | 260,173 | 0.46% | 4.63 | 1.58 |
| 2000 | 666 | 257,615 | 0.26% | 3.45 | 0.50 | 1,105 | 257,615 | 0.43% | 4.51 | 1.54 |
| 1999 | 536 | 248,597 | 0.22% | 3.48 | 0.51 | 891 | 248,597 | 0.36% | 4.49 | 1.48 |
| 1998 | 494 | 247,786 | 0.20% | 3.50 | 0.51 | 860 | 247,786 | 0.35% | 4.53 | 1.43 |
| 1997 | 423 | 246,197 | 0.17% | 3.53 | 0.50 | 757 | 246,197 | 0.31% | 4.59 | 1.43 |
| 1996 | 307 | 249,010 | 0.12% | 3.49 | 0.53 | 661 | 249,010 | 0.27% | 4.84 | 1.50 |
| 1995 | 262 | 246,348 | 0.11% | 3.50 | 0.52 | 529 | 246,348 | 0.21% | 4.80 | 1.53 |
| 1994 | 229 | 250,965 | 0.09% | 3.59 | 0.49 | 454 | 250,965 | 0.18% | 4.89 | 1.59 |
| 1993 | 190 | 255,610 | 0.07% | 3.56 | 0.52 | 396 | 255,610 | 0.15% | 4.85 | 1.49 |
| 1992 | 134 | 256,874 | 0.05% | 3.50 | 0.54 | 289 | 256,874 | 0.11% | 4.89 | 1.55 |
| 1991 | 121 | 249,796 | 0.05% | 3.44 | 0.50 | 250 | 249,796 | 0.10% | 4.84 | 1.61 |
| 1990 | 79 | 233,752 | 0.03% | 4.00 | 0.00 | 180 | 233,752 | 0.08% | 5.06 | 1.22 |

| (Table S2. continued) | | | | | | | | | | |
| --- | --- | --- | --- | --- | --- | --- | --- | --- | --- | --- |
| **Black** | | | | | | | | | | |
|  | **ASD by 4 Years** | | | | | **ASD by 8 Years** | | | | |
|  | **Cumulative Incidence** | | | **Average Age at Diagnosis** | | **Cumulative Incidence** | | | **Average Age at Diagnosis** | |
| **Birth**  **Year** | **ASD Cases** | **Total Births** | **Percent** | **Mean (Yrs)** | **SD (Yrs)** | **ASD Cases** | **Total Births** | **Percent** | **Mean (Yrs)** | **SD (Yrs)** |
| 2018 | 680 | 24,758 | 2.75% | 3.40 | 0.50 | --- | --- | --- | --- | --- |
| 2017 | 609 | 25,869 | 2.35% | 3.34 | 0.48 | --- | --- | --- | --- | --- |
| 2016 | 541 | 26,461 | 2.04% | 3.30 | 0.47 | --- | --- | --- | --- | --- |
| 2015 | 487 | 26,357 | 1.85% | 3.34 | 0.48 | --- | --- | --- | --- | --- |
| 2014 | 399 | 27,653 | 1.44% | 3.27 | 0.45 | 610 | 27,653 | 2.21% | 4.30 | 1.62 |
| 2013 | 357 | 28,273 | 1.26% | 3.32 | 0.48 | 558 | 28,273 | 1.97% | 4.33 | 1.55 |
| 2012 | 321 | 28,816 | 1.11% | 3.25 | 0.45 | 485 | 28,816 | 1.68% | 4.27 | 1.63 |
| 2011 | 317 | 29,383 | 1.08% | 3.28 | 0.49 | 462 | 29,383 | 1.57% | 4.23 | 1.58 |
| 2010 | 318 | 30,052 | 1.06% | 3.33 | 0.51 | 464 | 30,052 | 1.54% | 4.18 | 1.45 |
| 2009 | 299 | 30,681 | 0.97% | 3.30 | 0.49 | 435 | 30,681 | 1.42% | 4.12 | 1.41 |
| 2008 | 276 | 31,602 | 0.87% | 3.37 | 0.48 | 408 | 31,602 | 1.29% | 4.20 | 1.39 |
| 2007 | 215 | 31,440 | 0.68% | 3.35 | 0.48 | 341 | 31,440 | 1.08% | 4.40 | 1.57 |
| 2006 | 179 | 29,827 | 0.60% | 3.44 | 0.51 | 279 | 29,827 | 0.94% | 4.32 | 1.40 |
| 2005 | 196 | 30,405 | 0.64% | 3.36 | 0.50 | 311 | 30,405 | 1.02% | 4.36 | 1.51 |
| 2004 | 161 | 30,417 | 0.53% | 3.36 | 0.52 | 252 | 30,417 | 0.83% | 4.23 | 1.36 |
| 2003 | 163 | 30,820 | 0.53% | 3.36 | 0.49 | 258 | 30,820 | 0.84% | 4.33 | 1.49 |
| 2002 | 150 | 31,253 | 0.48% | 3.37 | 0.54 | 239 | 31,253 | 0.76% | 4.39 | 1.55 |
| 2001 | 138 | 32,354 | 0.43% | 3.40 | 0.52 | 218 | 32,354 | 0.67% | 4.34 | 1.44 |
| 2000 | 125 | 33,705 | 0.37% | 3.47 | 0.50 | 191 | 33,705 | 0.57% | 4.34 | 1.41 |
| 1999 | 110 | 34,023 | 0.32% | 3.44 | 0.50 | 178 | 34,023 | 0.52% | 4.46 | 1.52 |
| 1998 | 102 | 35,179 | 0.29% | 3.45 | 0.52 | 168 | 35,179 | 0.48% | 4.36 | 1.36 |
| 1997 | 95 | 35,637 | 0.27% | 3.60 | 0.53 | 159 | 35,637 | 0.45% | 4.53 | 1.36 |
| 1996 | 57 | 36,481 | 0.16% | 3.51 | 0.50 | 118 | 36,481 | 0.32% | 4.90 | 1.60 |
| 1995 | 63 | 38,414 | 0.16% | 3.51 | 0.50 | 126 | 38,414 | 0.33% | 4.85 | 1.60 |
| 1994 | 63 | 40,954 | 0.15% | 3.62 | 0.49 | 106 | 40,954 | 0.26% | 4.59 | 1.41 |
| 1993 | 63 | 42,724 | 0.15% | 3.48 | 0.56 | 111 | 42,724 | 0.26% | 4.64 | 1.59 |
| 1992 | 68 | 44,641 | 0.15% | 3.59 | 0.55 | 129 | 44,641 | 0.29% | 4.71 | 1.44 |
| 1991 | 56 | 45,652 | 0.12% | 3.55 | 0.50 | 99 | 45,652 | 0.22% | 4.60 | 1.38 |
| 1990 | 52 | 46,214 | 0.11% | 4.00 | 0.00 | 93 | 46,214 | 0.20% | 4.88 | 1.16 |

| (Table S2. continued) | | | | | | | | | | |  |
| --- | --- | --- | --- | --- | --- | --- | --- | --- | --- | --- | --- |
| **Asian and Pacific Islander** | | | | | | | | | | |  |
|  | **ASD by 4 Years** | | | | | **ASD by 8 Years** | | | | |  |
|  | **Cumulative Incidence** | | | **Average Age at Diagnosis** | | **Cumulative Incidence** | | | **Average Age at Diagnosis** | |  |
| **Birth Year** | **ASD Cases** | **Total Births** | **Percent** | **Mean (Yrs)** | **SD (Yrs)** | **ASD Cases** | **Total Births** | **Percent** | **Mean (Yrs)** | **SD (Yrs)** |  |
| 2018 | 1,174 | 72,259 | 1.62% | 3.37 | 0.50 | --- | --- | --- | --- | --- |  |
| 2017 | 1,028 | 75,997 | 1.35% | 3.31 | 0.47 | --- | --- | --- | --- | --- |  |
| 2016 | 1,021 | 77,882 | 1.31% | 3.27 | 0.45 | --- | --- | --- | --- | --- |  |
| 2015 | 945 | 75,135 | 1.26% | 3.27 | 0.46 | --- | --- | --- | --- | --- |  |
| 2014 | 908 | 79,252 | 1.15% | 3.21 | 0.44 | 1,254 | 79,252 | 1.58% | 3.96 | 1.40 |  |
| 2013 | 837 | 72,283 | 1.16% | 3.27 | 0.46 | 1,152 | 72,283 | 1.59% | 3.98 | 1.31 |  |
| 2012 | 761 | 72,811 | 1.05% | 3.28 | 0.47 | 1,096 | 72,811 | 1.51% | 4.10 | 1.41 |  |
| 2011 | 627 | 66,367 | 0.94% | 3.31 | 0.48 | 946 | 66,367 | 1.43% | 4.24 | 1.49 |  |
| 2010 | 615 | 65,276 | 0.94% | 3.34 | 0.50 | 910 | 65,276 | 1.39% | 4.25 | 1.50 |  |
| 2009 | 523 | 67,551 | 0.77% | 3.35 | 0.48 | 810 | 67,551 | 1.20% | 4.23 | 1.39 |  |
| 2008 | 516 | 69,560 | 0.74% | 3.34 | 0.47 | 745 | 69,560 | 1.07% | 4.21 | 1.49 |  |
| 2007 | 473 | 70,997 | 0.67% | 3.38 | 0.48 | 702 | 70,997 | 0.99% | 4.24 | 1.45 |  |
| 2006 | 361 | 55,844 | 0.65% | 3.40 | 0.50 | 534 | 55,844 | 0.96% | 4.27 | 1.48 |  |
| 2005 | 428 | 66,071 | 0.65% | 3.32 | 0.49 | 591 | 66,071 | 0.89% | 4.03 | 1.32 |  |
| 2004 | 408 | 66,458 | 0.61% | 3.33 | 0.49 | 593 | 66,458 | 0.89% | 4.13 | 1.39 |  |
| 2003 | 418 | 65,627 | 0.64% | 3.33 | 0.50 | 589 | 65,627 | 0.90% | 4.07 | 1.35 |  |
| 2002 | 377 | 63,340 | 0.60% | 3.33 | 0.50 | 561 | 63,340 | 0.89% | 4.17 | 1.39 |  |
| 2001 | 306 | 61,177 | 0.50% | 3.35 | 0.50 | 487 | 61,177 | 0.80% | 4.31 | 1.45 |  |
| 2000 | 270 | 62,649 | 0.43% | 3.42 | 0.51 | 445 | 62,649 | 0.71% | 4.49 | 1.57 |  |
| 1999 | 226 | 56,927 | 0.40% | 3.42 | 0.51 | 362 | 56,927 | 0.64% | 4.30 | 1.35 |  |
| 1998 | 213 | 55,528 | 0.38% | 3.52 | 0.51 | 361 | 55,528 | 0.65% | 4.50 | 1.41 |  |
| 1997 | 153 | 56,153 | 0.27% | 3.54 | 0.50 | 281 | 56,153 | 0.50% | 4.56 | 1.36 |  |
| 1996 | 132 | 55,244 | 0.24% | 3.41 | 0.49 | 261 | 55,244 | 0.47% | 4.63 | 1.47 |  |
| 1995 | 114 | 54,747 | 0.21% | 3.54 | 0.52 | 216 | 54,747 | 0.39% | 4.71 | 1.50 |  |
| 1994 | 101 | 55,374 | 0.18% | 3.55 | 0.52 | 191 | 55,374 | 0.34% | 4.64 | 1.40 |  |
| 1993 | 65 | 54,079 | 0.12% | 3.63 | 0.49 | 125 | 54,079 | 0.23% | 4.75 | 1.43 |  |
| 1992 | 59 | 55,592 | 0.11% | 3.58 | 0.53 | 132 | 55,592 | 0.24% | 4.84 | 1.41 |  |
| 1991 | 45 | 54,373 | 0.08% | 3.67 | 0.48 | 104 | 54,373 | 0.19% | 5.09 | 1.48 |  |
| 1990 | 31 | 52,915 | 0.06% | 4.00 | 0.00 | 71 | 52,915 | 0.13% | 5.08 | 1.24 |  |
| Notes: SD = Standard Deviation; ASD = Autism Spectrum Disorder; Yrs = Years | | | | | | | | | | |  |
|  |  |  |  |  |  |  |  |  |  |  |  |

| **Table S3.** ASD Cumulative Incidence and Average Age at Diagnosis by Birth Year Cohort – Stratified by Sex | | | | | | | | | | |
| --- | --- | --- | --- | --- | --- | --- | --- | --- | --- | --- |
| **Male** | | | | | | | | | | |
|  | **ASD by 4 Years** | | | | | **ASD by 8 Years** | | | | |
|  | **Cumulative Incidence** | | | **Average Age at Diagnosis** | | **Cumulative Incidence** | | | **Average Age at Diagnosis** | |
| **Birth Year** | **ASD Cases** | **Total Births** | **Percent** | **Mean (Yrs)** | **SD (Yrs)** | **ASD Cases** | **Total Births** | **Percent** | **Mean (Yrs)** | **SD (Yrs)** |
| 2018 | 6,823 | 231,965 | 2.94% | 3.37 | 0.49 | --- | --- | --- | --- | --- |
| 2017 | 6,179 | 240,894 | 2.57% | 3.33 | 0.48 | --- | --- | --- | --- | --- |
| 2016 | 6,057 | 249,285 | 2.43% | 3.27 | 0.45 | --- | --- | --- | --- | --- |
| 2015 | 5,268 | 251,064 | 2.10% | 3.30 | 0.47 | --- | --- | --- | --- | --- |
| 2014 | 4,627 | 256,481 | 1.80% | 3.26 | 0.46 | 6,754 | 256,481 | 2.63% | 4.15 | 1.50 |
| 2013 | 4,158 | 252,216 | 1.65% | 3.28 | 0.46 | 6,130 | 252,216 | 2.43% | 4.18 | 1.49 |
| 2012 | 3,852 | 256,189 | 1.50% | 3.28 | 0.47 | 5,850 | 256,189 | 2.28% | 4.24 | 1.51 |
| 2011 | 3,436 | 256,473 | 1.34% | 3.31 | 0.48 | 5,362 | 256,473 | 2.09% | 4.33 | 1.56 |
| 2010 | 3,357 | 261,047 | 1.29% | 3.33 | 0.50 | 5,053 | 261,047 | 1.94% | 4.27 | 1.52 |
| 2009 | 2,945 | 268,964 | 1.09% | 3.31 | 0.48 | 4,613 | 268,964 | 1.72% | 4.32 | 1.53 |
| 2008 | 2,772 | 281,855 | 0.98% | 3.37 | 0.48 | 4,376 | 281,855 | 1.55% | 4.38 | 1.53 |
| 2007 | 2,495 | 288,935 | 0.86% | 3.38 | 0.49 | 3,959 | 288,935 | 1.37% | 4.41 | 1.55 |
| 2006 | 2,351 | 287,368 | 0.82% | 3.40 | 0.50 | 3,585 | 287,368 | 1.25% | 4.34 | 1.50 |
| 2005 | 2,270 | 279,712 | 0.81% | 3.36 | 0.51 | 3,344 | 279,712 | 1.20% | 4.23 | 1.46 |
| 2004 | 2,183 | 277,953 | 0.79% | 3.34 | 0.50 | 3,233 | 277,953 | 1.16% | 4.20 | 1.43 |
| 2003 | 2,099 | 276,382 | 0.76% | 3.36 | 0.50 | 3,206 | 276,382 | 1.16% | 4.25 | 1.43 |
| 2002 | 1,705 | 269,563 | 0.63% | 3.39 | 0.51 | 2,806 | 269,563 | 1.04% | 4.41 | 1.46 |
| 2001 | 1,605 | 268,472 | 0.60% | 3.38 | 0.51 | 2,583 | 268,472 | 0.96% | 4.42 | 1.53 |
| 2000 | 1,459 | 271,729 | 0.54% | 3.43 | 0.51 | 2,328 | 271,729 | 0.86% | 4.43 | 1.53 |
| 1999 | 1,215 | 264,160 | 0.46% | 3.42 | 0.51 | 1,974 | 264,160 | 0.75% | 4.43 | 1.50 |
| 1998 | 1,147 | 266,468 | 0.43% | 3.47 | 0.51 | 1,880 | 266,468 | 0.71% | 4.44 | 1.43 |
| 1997 | 979 | 264,288 | 0.37% | 3.53 | 0.51 | 1,669 | 264,288 | 0.63% | 4.53 | 1.42 |
| 1996 | 769 | 268,095 | 0.29% | 3.46 | 0.51 | 1,498 | 268,095 | 0.56% | 4.71 | 1.52 |
| 1995 | 671 | 272,345 | 0.25% | 3.48 | 0.52 | 1,265 | 272,345 | 0.46% | 4.72 | 1.54 |
| 1994 | 625 | 281,214 | 0.22% | 3.53 | 0.52 | 1,162 | 281,214 | 0.41% | 4.77 | 1.58 |
| 1993 | 507 | 290,158 | 0.17% | 3.53 | 0.55 | 984 | 290,158 | 0.34% | 4.79 | 1.56 |
| 1992 | 447 | 298,472 | 0.15% | 3.47 | 0.58 | 881 | 298,472 | 0.30% | 4.74 | 1.56 |
| 1991 | 371 | 299,153 | 0.12% | 3.55 | 0.50 | 703 | 299,153 | 0.23% | 4.76 | 1.50 |
| 1990 | 273 | 295,899 | 0.09% | 4.00 | 0.00 | 539 | 295,899 | 0.18% | 5.03 | 1.30 |

| (Table S3. continued) | | | | | | | | | | |  |
| --- | --- | --- | --- | --- | --- | --- | --- | --- | --- | --- | --- |
| **Female** | | | | | | | | | | |  |
|  | **ASD by 4 Years** | | | | | **ASD by 8 Years** | | | | |  |
|  | **Cumulative Incidence** | | | **Average Age at Diagnosis** | | **Cumulative Incidence** | | | **Average Age at Diagnosis** | |  |
| **Birth Year** | **ASD Cases** | **Total Births** | **Percent** | **Mean (Yrs)** | **SD (Yrs)** | **ASD Cases** | **Total Births** | **Percent** | **Mean (Yrs)** | **SD (Yrs)** |  |
| 2018 | 2,228 | 222,172 | 1.00% | 3.37 | 0.49 | --- | --- | --- | --- | --- |  |
| 2017 | 1,873 | 228,772 | 0.82% | 3.34 | 0.48 | --- | --- | --- | --- | --- |  |
| 2016 | 1,697 | 237,374 | 0.71% | 3.27 | 0.45 | --- | --- | --- | --- | --- |  |
| 2015 | 1,460 | 238,462 | 0.61% | 3.30 | 0.47 | --- | --- | --- | --- | --- |  |
| 2014 | 1,302 | 243,980 | 0.53% | 3.28 | 0.47 | 1,818 | 243,980 | 0.75% | 4.07 | 1.45 |  |
| 2013 | 1,057 | 240,010 | 0.44% | 3.28 | 0.47 | 1,554 | 240,010 | 0.65% | 4.15 | 1.45 |  |
| 2012 | 968 | 245,054 | 0.40% | 3.29 | 0.48 | 1,388 | 245,054 | 0.57% | 4.14 | 1.47 |  |
| 2011 | 819 | 244,174 | 0.34% | 3.29 | 0.46 | 1,279 | 244,174 | 0.52% | 4.31 | 1.56 |  |
| 2010 | 795 | 248,845 | 0.32% | 3.33 | 0.49 | 1,187 | 248,845 | 0.48% | 4.26 | 1.52 |  |
| 2009 | 618 | 256,092 | 0.24% | 3.33 | 0.49 | 946 | 256,092 | 0.37% | 4.30 | 1.53 |  |
| 2008 | 640 | 268,235 | 0.24% | 3.34 | 0.48 | 911 | 268,235 | 0.34% | 4.17 | 1.44 |  |
| 2007 | 506 | 275,411 | 0.18% | 3.39 | 0.49 | 784 | 275,411 | 0.28% | 4.33 | 1.46 |  |
| 2006 | 480 | 273,412 | 0.18% | 3.42 | 0.50 | 724 | 273,412 | 0.26% | 4.32 | 1.47 |  |
| 2005 | 517 | 267,280 | 0.19% | 3.33 | 0.53 | 726 | 267,280 | 0.27% | 4.16 | 1.49 |  |
| 2004 | 434 | 266,024 | 0.16% | 3.34 | 0.50 | 635 | 266,024 | 0.24% | 4.17 | 1.43 |  |
| 2003 | 408 | 263,388 | 0.15% | 3.36 | 0.50 | 616 | 263,388 | 0.23% | 4.20 | 1.39 |  |
| 2002 | 397 | 258,052 | 0.15% | 3.33 | 0.50 | 598 | 258,052 | 0.23% | 4.26 | 1.50 |  |
| 2001 | 299 | 257,435 | 0.12% | 3.34 | 0.53 | 476 | 257,435 | 0.18% | 4.39 | 1.57 |  |
| 2000 | 317 | 258,919 | 0.12% | 3.36 | 0.49 | 490 | 258,919 | 0.19% | 4.32 | 1.50 |  |
| 1999 | 279 | 252,535 | 0.11% | 3.46 | 0.51 | 421 | 252,535 | 0.17% | 4.31 | 1.41 |  |
| 1998 | 249 | 254,746 | 0.10% | 3.43 | 0.51 | 388 | 254,746 | 0.15% | 4.33 | 1.42 |  |
| 1997 | 198 | 253,756 | 0.08% | 3.51 | 0.52 | 341 | 253,756 | 0.13% | 4.48 | 1.37 |  |
| 1996 | 155 | 256,399 | 0.06% | 3.46 | 0.54 | 279 | 256,399 | 0.11% | 4.59 | 1.51 |  |
| 1995 | 152 | 259,232 | 0.06% | 3.44 | 0.51 | 278 | 259,232 | 0.11% | 4.65 | 1.57 |  |
| 1994 | 121 | 269,168 | 0.04% | 3.45 | 0.53 | 222 | 269,168 | 0.08% | 4.70 | 1.66 |  |
| 1993 | 120 | 276,805 | 0.04% | 3.58 | 0.54 | 216 | 276,805 | 0.08% | 4.63 | 1.41 |  |
| 1992 | 95 | 283,877 | 0.03% | 3.46 | 0.58 | 196 | 283,877 | 0.07% | 4.77 | 1.52 |  |
| 1991 | 98 | 285,878 | 0.03% | 3.41 | 0.49 | 170 | 285,878 | 0.06% | 4.53 | 1.53 |  |
| 1990 | 53 | 281,036 | 0.02% | 4.00 | 0.00 | 118 | 281,036 | 0.04% | 5.04 | 1.17 |  |
| Notes: SD = Standard Deviation; ASD = Autism Spectrum Disorder; Yrs = Years | | | | | | | | | | |  |
|  |  |  |  |  |  |  |  |  |  |  |  |

| **Table S4.** ASD Cumulative Incidence and Average Age at Diagnosis by Birth Year Cohort – Stratified by Nativity | | | | | | | | | | |
| --- | --- | --- | --- | --- | --- | --- | --- | --- | --- | --- |
| **US Born** | | | | | | | | | | |
|  | **ASD by 4 Years** | | | | | **ASD by 8 Years** | | | | |
|  | **Cumulative Incidence** | | | **Average Age at Diagnosis** | | **Cumulative Incidence** | | | **Average Age at Diagnosis** | |
| **Birth Year** | **ASD Cases** | **Total Births** | **Percent** | **Mean (Yrs)** | **SD (Yrs)** | **ASD Cases** | **Total Births** | **Percent** | **Mean (Yrs)** | **SD (Yrs)** |
| 2018 | 5,733 | 290,746 | 1.97% | 3.37 | 0.49 | --- | --- | --- | --- | --- |
| 2017 | 5,146 | 296,206 | 1.74% | 3.34 | 0.48 | --- | --- | --- | --- | --- |
| 2016 | 4,863 | 304,264 | 1.60% | 3.27 | 0.45 | --- | --- | --- | --- | --- |
| 2015 | 4,050 | 306,605 | 1.32% | 3.31 | 0.47 | --- | --- | --- | --- | --- |
| 2014 | 3,492 | 309,825 | 1.13% | 3.27 | 0.47 | 5,128 | 309,825 | 1.66% | 4.17 | 1.52 |
| 2013 | 3,088 | 305,259 | 1.01% | 3.29 | 0.47 | 4,626 | 305,259 | 1.52% | 4.22 | 1.52 |
| 2012 | 2,845 | 303,907 | 0.94% | 3.28 | 0.47 | 4,311 | 303,907 | 1.42% | 4.24 | 1.53 |
| 2011 | 2,500 | 301,477 | 0.83% | 3.30 | 0.47 | 3,896 | 301,477 | 1.29% | 4.33 | 1.58 |
| 2010 | 2,414 | 299,994 | 0.80% | 3.31 | 0.50 | 3,648 | 299,994 | 1.22% | 4.26 | 1.53 |
| 2009 | 2,074 | 299,831 | 0.69% | 3.30 | 0.48 | 3,179 | 299,831 | 1.06% | 4.30 | 1.56 |
| 2008 | 1,982 | 304,182 | 0.65% | 3.37 | 0.48 | 3,047 | 304,182 | 1.00% | 4.32 | 1.50 |
| 2007 | 1,679 | 304,366 | 0.55% | 3.38 | 0.49 | 2,639 | 304,366 | 0.87% | 4.40 | 1.55 |
| 2006 | 1,614 | 300,588 | 0.54% | 3.40 | 0.50 | 2,424 | 300,588 | 0.81% | 4.32 | 1.50 |
| 2005 | 1,603 | 291,744 | 0.55% | 3.34 | 0.51 | 2,318 | 291,744 | 0.79% | 4.20 | 1.49 |
| 2004 | 1,427 | 289,023 | 0.49% | 3.34 | 0.50 | 2,088 | 289,023 | 0.72% | 4.18 | 1.43 |
| 2003 | 1,393 | 288,865 | 0.48% | 3.34 | 0.50 | 2,117 | 288,865 | 0.73% | 4.24 | 1.44 |
| 2002 | 1,175 | 281,775 | 0.42% | 3.36 | 0.51 | 1,908 | 281,775 | 0.68% | 4.39 | 1.50 |
| 2001 | 1,132 | 282,172 | 0.40% | 3.36 | 0.51 | 1,741 | 282,172 | 0.62% | 4.34 | 1.53 |
| 2000 | 1,047 | 287,835 | 0.36% | 3.39 | 0.51 | 1,614 | 287,835 | 0.56% | 4.35 | 1.52 |
| 1999 | 863 | 284,849 | 0.30% | 3.41 | 0.51 | 1,372 | 284,849 | 0.48% | 4.41 | 1.52 |
| 1998 | 830 | 290,360 | 0.29% | 3.44 | 0.51 | 1,282 | 290,360 | 0.44% | 4.33 | 1.43 |
| 1997 | 701 | 284,200 | 0.25% | 3.53 | 0.51 | 1,157 | 284,200 | 0.41% | 4.49 | 1.42 |
| 1996 | 568 | 285,652 | 0.20% | 3.46 | 0.51 | 1,013 | 285,652 | 0.35% | 4.61 | 1.52 |
| 1995 | 509 | 293,548 | 0.17% | 3.44 | 0.51 | 937 | 293,548 | 0.32% | 4.66 | 1.56 |
| 1994 | 491 | 304,711 | 0.16% | 3.51 | 0.52 | 873 | 304,711 | 0.29% | 4.73 | 1.63 |
| 1993 | 421 | 311,485 | 0.14% | 3.52 | 0.56 | 757 | 311,485 | 0.24% | 4.69 | 1.57 |
| 1992 | 391 | 321,870 | 0.12% | 3.43 | 0.59 | 717 | 321,870 | 0.22% | 4.61 | 1.55 |
| 1991 | 330 | 331,401 | 0.10% | 3.53 | 0.50 | 574 | 331,401 | 0.17% | 4.58 | 1.44 |
| 1990 | 231 | 337,032 | 0.07% | 4.00 | 0.00 | 441 | 337,032 | 0.13% | 5.01 | 1.30 |

| (Table S4. continued) | | | | | | | | | | |  |
| --- | --- | --- | --- | --- | --- | --- | --- | --- | --- | --- | --- |
| **Foreign Born** | | | | | | | | | | |  |
|  | **ASD by 4 Years** | | | | | **ASD by 8 Years** | | | | |  |
|  | **Cumulative Incidence** | | | **Average Age at Diagnosis** | | **Cumulative Incidence** | | | **Average Age at Diagnosis** | |  |
| **Birth Year** | **ASD Cases** | **Total Births** | **Percent** | **Mean (Yrs)** | **SD (Yrs)** | **ASD Cases** | **Total Births** | **Percent** | **Mean (Yrs)** | **SD (Yrs)** |  |
| 2018 | 3,318 | 163,398 | 2.03% | 3.36 | 0.49 | --- | --- | --- | --- | --- |  |
| 2017 | 2,906 | 173,468 | 1.68% | 3.31 | 0.47 | --- | --- | --- | --- | --- |  |
| 2016 | 2,891 | 182,397 | 1.59% | 3.26 | 0.45 | --- | --- | --- | --- | --- |  |
| 2015 | 2,678 | 182,928 | 1.46% | 3.29 | 0.47 | --- | --- | --- | --- | --- |  |
| 2014 | 2,437 | 190,640 | 1.28% | 3.26 | 0.46 | 3,444 | 190,640 | 1.81% | 4.08 | 1.45 |  |
| 2013 | 2,127 | 186,976 | 1.14% | 3.27 | 0.46 | 3,058 | 186,976 | 1.64% | 4.10 | 1.42 |  |
| 2012 | 1,975 | 197,342 | 1.00% | 3.30 | 0.47 | 2,927 | 197,342 | 1.48% | 4.19 | 1.46 |  |
| 2011 | 1,755 | 199,173 | 0.88% | 3.32 | 0.48 | 2,745 | 199,173 | 1.38% | 4.33 | 1.53 |  |
| 2010 | 1,738 | 209,904 | 0.83% | 3.35 | 0.50 | 2,592 | 209,904 | 1.23% | 4.27 | 1.51 |  |
| 2009 | 1,489 | 225,230 | 0.66% | 3.33 | 0.49 | 2,380 | 225,230 | 1.06% | 4.34 | 1.49 |  |
| 2008 | 1,430 | 245,918 | 0.58% | 3.36 | 0.48 | 2,240 | 245,918 | 0.91% | 4.37 | 1.54 |  |
| 2007 | 1,322 | 259,988 | 0.51% | 3.39 | 0.49 | 2,104 | 259,988 | 0.81% | 4.40 | 1.51 |  |
| 2006 | 1,217 | 260,200 | 0.47% | 3.41 | 0.49 | 1,885 | 260,200 | 0.72% | 4.35 | 1.49 |  |
| 2005 | 1,184 | 255,258 | 0.46% | 3.39 | 0.51 | 1,752 | 255,258 | 0.69% | 4.25 | 1.43 |  |
| 2004 | 1,190 | 254,963 | 0.47% | 3.34 | 0.49 | 1,780 | 254,963 | 0.70% | 4.22 | 1.44 |  |
| 2003 | 1,114 | 250,917 | 0.44% | 3.39 | 0.51 | 1,705 | 250,917 | 0.68% | 4.25 | 1.39 |  |
| 2002 | 927 | 245,846 | 0.38% | 3.41 | 0.51 | 1,496 | 245,846 | 0.61% | 4.37 | 1.42 |  |
| 2001 | 772 | 243,748 | 0.32% | 3.40 | 0.51 | 1,318 | 243,748 | 0.54% | 4.52 | 1.54 |  |
| 2000 | 729 | 242,820 | 0.30% | 3.45 | 0.50 | 1,204 | 242,820 | 0.50% | 4.49 | 1.52 |  |
| 1999 | 626 | 230,522 | 0.27% | 3.45 | 0.51 | 1,016 | 230,522 | 0.44% | 4.40 | 1.44 |  |
| 1998 | 558 | 229,457 | 0.24% | 3.51 | 0.51 | 974 | 229,457 | 0.42% | 4.53 | 1.42 |  |
| 1997 | 476 | 233,620 | 0.20% | 3.52 | 0.50 | 853 | 233,620 | 0.37% | 4.56 | 1.40 |  |
| 1996 | 356 | 238,653 | 0.15% | 3.46 | 0.52 | 764 | 238,653 | 0.32% | 4.80 | 1.50 |  |
| 1995 | 314 | 237,863 | 0.13% | 3.53 | 0.52 | 606 | 237,863 | 0.25% | 4.76 | 1.52 |  |
| 1994 | 254 | 245,418 | 0.10% | 3.54 | 0.52 | 509 | 245,418 | 0.21% | 4.81 | 1.53 |  |
| 1993 | 205 | 255,227 | 0.08% | 3.58 | 0.50 | 442 | 255,227 | 0.17% | 4.89 | 1.47 |  |
| 1992 | 151 | 260,266 | 0.06% | 3.57 | 0.56 | 360 | 260,266 | 0.14% | 5.02 | 1.51 |  |
| 1991 | 139 | 253,396 | 0.05% | 3.50 | 0.50 | 299 | 253,396 | 0.12% | 4.96 | 1.60 |  |
| 1990 | 95 | 239,580 | 0.04% | 4.00 | 0.00 | 216 | 239,580 | 0.09% | 5.07 | 1.24 |  |
| Notes: SD = Standard Deviation; ASD = Autism Spectrum Disorder; Yrs = Years | | | | | | | | | | |  |
|  |  |  |  |  |  |  |  |  |  |  |  |

| **Table S5.** ASD Cumulative Incidence and Average Age at Diagnosis by Birth Year Cohort – Stratified by Maternal Education | | | | | | | | | | |
| --- | --- | --- | --- | --- | --- | --- | --- | --- | --- | --- |
| **≤ 8th Grade** | | | | | | | | | | |
|  | **ASD by 4 Years** | | | | | **ASD by 8 Years** | | | | |
|  | **Cumulative Incidence** | | | **Average Age at Diagnosis** | | **Cumulative Incidence** | | | **Average Age at Diagnosis** | |
| **Birth Year** | **ASD Cases** | **Total Births** | **Percent** | **Mean (Yrs)** | **SD (Yrs)** | **ASD Cases** | **Total Births** | **Percent** | **Mean (Yrs)** | **SD (Yrs)** |
| 2018 | 302 | 16,685 | 1.81% | 3.35 | 0.48 | --- | --- | --- | --- | --- |
| 2017 | 247 | 18,161 | 1.36% | 3.36 | 0.49 | --- | --- | --- | --- | --- |
| 2016 | 271 | 20,105 | 1.35% | 3.27 | 0.45 | --- | --- | --- | --- | --- |
| 2015 | 271 | 22,267 | 1.22% | 3.31 | 0.49 | --- | --- | --- | --- | --- |
| 2014 | 229 | 24,289 | 0.94% | 3.24 | 0.45 | 369 | 24,289 | 1.52% | 4.28 | 1.54 |
| 2013 | 194 | 26,003 | 0.75% | 3.37 | 0.48 | 321 | 26,003 | 1.23% | 4.41 | 1.48 |
| 2012 | 184 | 29,434 | 0.63% | 3.38 | 0.50 | 313 | 29,434 | 1.06% | 4.49 | 1.52 |
| 2011 | 217 | 32,927 | 0.66% | 3.35 | 0.49 | 356 | 32,927 | 1.08% | 4.43 | 1.54 |
| 2010 | 183 | 36,442 | 0.50% | 3.37 | 0.52 | 309 | 36,442 | 0.85% | 4.41 | 1.47 |
| 2009 | 170 | 42,009 | 0.40% | 3.31 | 0.49 | 277 | 42,009 | 0.66% | 4.46 | 1.67 |
| 2008 | 156 | 48,144 | 0.32% | 3.38 | 0.49 | 300 | 48,144 | 0.62% | 4.72 | 1.63 |
| 2007 | 158 | 52,943 | 0.30% | 3.44 | 0.50 | 274 | 52,943 | 0.52% | 4.59 | 1.57 |
| 2006 | 150 | 56,621 | 0.26% | 3.39 | 0.49 | 259 | 56,621 | 0.46% | 4.53 | 1.54 |
| 2005 | 154 | 57,739 | 0.27% | 3.42 | 0.53 | 241 | 57,739 | 0.42% | 4.43 | 1.53 |
| 2004 | 152 | 57,847 | 0.26% | 3.30 | 0.50 | 255 | 57,847 | 0.44% | 4.38 | 1.51 |
| 2003 | 153 | 58,624 | 0.26% | 3.47 | 0.50 | 253 | 58,624 | 0.43% | 4.46 | 1.43 |
| 2002 | 115 | 59,358 | 0.19% | 3.56 | 0.50 | 219 | 59,358 | 0.37% | 4.69 | 1.42 |
| 2001 | 70 | 61,764 | 0.11% | 3.46 | 0.50 | 155 | 61,764 | 0.25% | 4.95 | 1.61 |
| 2000 | 88 | 62,781 | 0.14% | 3.49 | 0.50 | 161 | 62,781 | 0.26% | 4.59 | 1.47 |
| 1999 | 83 | 62,147 | 0.13% | 3.54 | 0.50 | 158 | 62,147 | 0.25% | 4.66 | 1.45 |
| 1998 | 73 | 64,253 | 0.11% | 3.56 | 0.53 | 134 | 64,253 | 0.21% | 4.73 | 1.52 |
| 1997 | 60 | 68,932 | 0.09% | 3.57 | 0.50 | 132 | 68,932 | 0.19% | 4.93 | 1.52 |
| 1996 | 44 | 74,683 | 0.06% | 3.45 | 0.59 | 124 | 74,683 | 0.17% | 5.20 | 1.55 |
| 1995 | 58 | 78,877 | 0.07% | 3.59 | 0.50 | 127 | 78,877 | 0.16% | 4.81 | 1.40 |
| 1994 | 40 | 88,357 | 0.05% | 3.55 | 0.50 | 87 | 88,357 | 0.10% | 4.93 | 1.60 |
| 1993 | 33 | 91,105 | 0.04% | 3.58 | 0.50 | 78 | 91,105 | 0.09% | 4.96 | 1.46 |
| 1992 | 23 | 97,517 | 0.02% | 3.65 | 0.57 | 66 | 97,517 | 0.07% | 5.41 | 1.60 |
| 1991 | 34 | 97,962 | 0.03% | 3.24 | 0.43 | 72 | 97,962 | 0.07% | 4.68 | 1.60 |
| 1990 | 18 | 95,615 | 0.02% | 4.00 | 0.00 | 41 | 95,615 | 0.04% | 4.93 | 1.06 |

| (Table S5. continued) | | | | | | | | | | |
| --- | --- | --- | --- | --- | --- | --- | --- | --- | --- | --- |
| **9th - 12th Grade** | | | | | | | | | | |
|  | **ASD by 4 Years** | | | | | **ASD by 8 Years** | | | | |
|  | **Cumulative Incidence** | | | **Average Age at Diagnosis** | | **Cumulative Incidence** | | | **Average Age at Diagnosis** | |
| **Birth Year** | **ASD Cases** | **Total Births** | **Percent** | **Mean (Yrs)** | **SD (Yrs)** | **ASD Cases** | **Total Births** | **Percent** | **Mean (Yrs)** | **SD (Yrs)** |
| 2018 | 947 | 38,713 | 2.45% | 3.39 | 0.49 | --- | --- | --- | --- | --- |
| 2017 | 810 | 42,850 | 1.89% | 3.36 | 0.49 | --- | --- | --- | --- | --- |
| 2016 | 843 | 47,690 | 1.77% | 3.29 | 0.47 | --- | --- | --- | --- | --- |
| 2015 | 722 | 51,892 | 1.39% | 3.33 | 0.49 | --- | --- | --- | --- | --- |
| 2014 | 689 | 57,280 | 1.20% | 3.32 | 0.48 | 1,007 | 57,280 | 1.76% | 4.21 | 1.51 |
| 2013 | 573 | 61,427 | 0.93% | 3.31 | 0.49 | 904 | 61,427 | 1.47% | 4.36 | 1.58 |
| 2012 | 545 | 68,004 | 0.80% | 3.32 | 0.48 | 872 | 68,004 | 1.28% | 4.38 | 1.56 |
| 2011 | 553 | 73,686 | 0.75% | 3.32 | 0.49 | 916 | 73,686 | 1.24% | 4.45 | 1.59 |
| 2010 | 520 | 80,093 | 0.65% | 3.34 | 0.50 | 835 | 80,093 | 1.04% | 4.38 | 1.52 |
| 2009 | 488 | 85,532 | 0.57% | 3.37 | 0.49 | 805 | 85,532 | 0.94% | 4.48 | 1.58 |
| 2008 | 435 | 94,127 | 0.46% | 3.42 | 0.49 | 693 | 94,127 | 0.74% | 4.46 | 1.56 |
| 2007 | 409 | 98,648 | 0.41% | 3.43 | 0.50 | 670 | 98,648 | 0.68% | 4.50 | 1.56 |
| 2006 | 598 | 147,773 | 0.40% | 3.41 | 0.49 | 971 | 147,773 | 0.66% | 4.45 | 1.52 |
| 2005 | 313 | 91,253 | 0.34% | 3.42 | 0.53 | 513 | 91,253 | 0.56% | 4.48 | 1.53 |
| 2004 | 287 | 90,492 | 0.32% | 3.37 | 0.49 | 463 | 90,492 | 0.51% | 4.37 | 1.49 |
| 2003 | 258 | 89,490 | 0.29% | 3.44 | 0.51 | 429 | 89,490 | 0.48% | 4.39 | 1.38 |
| 2002 | 224 | 89,385 | 0.25% | 3.50 | 0.51 | 392 | 89,385 | 0.44% | 4.57 | 1.46 |
| 2001 | 208 | 91,794 | 0.23% | 3.43 | 0.52 | 369 | 91,794 | 0.40% | 4.60 | 1.55 |
| 2000 | 189 | 92,833 | 0.20% | 3.43 | 0.50 | 324 | 92,833 | 0.35% | 4.60 | 1.62 |
| 1999 | 123 | 92,401 | 0.13% | 3.49 | 0.52 | 228 | 92,401 | 0.25% | 4.75 | 1.64 |
| 1998 | 148 | 94,577 | 0.16% | 3.47 | 0.51 | 269 | 94,577 | 0.28% | 4.52 | 1.39 |
| 1997 | 111 | 95,761 | 0.12% | 3.57 | 0.53 | 217 | 95,761 | 0.23% | 4.64 | 1.34 |
| 1996 | 97 | 99,486 | 0.10% | 3.47 | 0.50 | 202 | 99,486 | 0.20% | 4.77 | 1.51 |
| 1995 | 82 | 100,779 | 0.08% | 3.51 | 0.50 | 177 | 100,779 | 0.18% | 4.92 | 1.56 |
| 1994 | 82 | 103,539 | 0.08% | 3.60 | 0.49 | 161 | 103,539 | 0.16% | 5.01 | 1.68 |
| 1993 | 52 | 106,988 | 0.05% | 3.58 | 0.54 | 133 | 106,988 | 0.12% | 5.04 | 1.42 |
| 1992 | 42 | 108,777 | 0.04% | 3.50 | 0.55 | 101 | 108,777 | 0.09% | 4.96 | 1.52 |
| 1991 | 40 | 107,931 | 0.04% | 3.65 | 0.48 | 75 | 107,931 | 0.07% | 4.83 | 1.52 |
| 1990 | 40 | 101,653 | 0.04% | 4.00 | 0.00 | 74 | 101,653 | 0.07% | 4.92 | 1.27 |

| (Table S5. continued) | | | | | | | | | | |
| --- | --- | --- | --- | --- | --- | --- | --- | --- | --- | --- |
| **High School Graduate** | | | | | | | | | | |
|  | **ASD by 4 Years** | | | | | **ASD by 8 Years** | | | | |
|  | **Cumulative Incidence** | | | **Average Age at Diagnosis** | | **Cumulative Incidence** | | | **Average Age at Diagnosis** | |
| **Birth Year** | **ASD Cases** | **Total Births** | **Percent** | **Mean (Yrs)** | **SD (Yrs)** | **ASD Cases** | **Total Births** | **Percent** | **Mean (Yrs)** | **SD (Yrs)** |
| 2018 | 2,467 | 108,318 | 2.28% | 3.37 | 0.49 | --- | --- | --- | --- | --- |
| 2017 | 2,239 | 112,418 | 1.99% | 3.35 | 0.49 | --- | --- | --- | --- | --- |
| 2016 | 2,129 | 116,739 | 1.82% | 3.28 | 0.46 | --- | --- | --- | --- | --- |
| 2015 | 1,874 | 119,336 | 1.57% | 3.30 | 0.47 | --- | --- | --- | --- | --- |
| 2014 | 1,558 | 121,013 | 1.29% | 3.29 | 0.46 | 2,316 | 121,013 | 1.91% | 4.19 | 1.48 |
| 2013 | 1,432 | 121,167 | 1.18% | 3.29 | 0.46 | 2,105 | 121,167 | 1.74% | 4.17 | 1.45 |
| 2012 | 1,203 | 122,929 | 0.98% | 3.30 | 0.48 | 1,849 | 122,929 | 1.50% | 4.25 | 1.49 |
| 2011 | 1,038 | 123,905 | 0.84% | 3.31 | 0.47 | 1,689 | 123,905 | 1.36% | 4.40 | 1.58 |
| 2010 | 1,124 | 128,422 | 0.88% | 3.33 | 0.50 | 1,661 | 128,422 | 1.29% | 4.26 | 1.54 |
| 2009 | 920 | 134,825 | 0.68% | 3.33 | 0.48 | 1,472 | 134,825 | 1.09% | 4.36 | 1.53 |
| 2008 | 907 | 143,895 | 0.63% | 3.37 | 0.48 | 1,421 | 143,895 | 0.99% | 4.33 | 1.48 |
| 2007 | 756 | 148,694 | 0.51% | 3.38 | 0.49 | 1,259 | 148,694 | 0.85% | 4.50 | 1.57 |
| 2006 | 486 | 99,389 | 0.49% | 3.44 | 0.51 | 755 | 99,389 | 0.76% | 4.42 | 1.54 |
| 2005 | 731 | 150,736 | 0.48% | 3.37 | 0.51 | 1,117 | 150,736 | 0.74% | 4.30 | 1.49 |
| 2004 | 695 | 149,399 | 0.47% | 3.35 | 0.49 | 1,058 | 149,399 | 0.71% | 4.26 | 1.46 |
| 2003 | 626 | 149,557 | 0.42% | 3.36 | 0.51 | 1,028 | 149,557 | 0.69% | 4.37 | 1.48 |
| 2002 | 508 | 147,605 | 0.34% | 3.36 | 0.51 | 861 | 147,605 | 0.58% | 4.47 | 1.52 |
| 2001 | 472 | 147,915 | 0.32% | 3.41 | 0.51 | 797 | 147,915 | 0.54% | 4.56 | 1.58 |
| 2000 | 447 | 150,485 | 0.30% | 3.45 | 0.51 | 767 | 150,485 | 0.51% | 4.54 | 1.51 |
| 1999 | 387 | 148,812 | 0.26% | 3.49 | 0.52 | 650 | 148,812 | 0.44% | 4.52 | 1.47 |
| 1998 | 361 | 150,672 | 0.24% | 3.48 | 0.52 | 605 | 150,672 | 0.40% | 4.51 | 1.48 |
| 1997 | 310 | 149,050 | 0.21% | 3.55 | 0.51 | 564 | 149,050 | 0.38% | 4.63 | 1.41 |
| 1996 | 236 | 151,242 | 0.16% | 3.45 | 0.50 | 485 | 151,242 | 0.32% | 4.68 | 1.41 |
| 1995 | 213 | 155,113 | 0.14% | 3.46 | 0.51 | 415 | 155,113 | 0.27% | 4.74 | 1.56 |
| 1994 | 199 | 161,462 | 0.12% | 3.53 | 0.51 | 369 | 161,462 | 0.23% | 4.73 | 1.55 |
| 1993 | 183 | 167,019 | 0.11% | 3.51 | 0.58 | 359 | 167,019 | 0.21% | 4.81 | 1.59 |
| 1992 | 136 | 171,912 | 0.08% | 3.45 | 0.59 | 286 | 171,912 | 0.17% | 4.78 | 1.54 |
| 1991 | 135 | 175,596 | 0.08% | 3.56 | 0.50 | 243 | 175,596 | 0.14% | 4.65 | 1.45 |
| 1990 | 94 | 178,351 | 0.05% | 4.00 | 0.00 | 191 | 178,351 | 0.11% | 4.99 | 1.23 |

| (Table S5. continued) | | | | | | | | | | |
| --- | --- | --- | --- | --- | --- | --- | --- | --- | --- | --- |
| **Some College +** | | | | | | | | | | |
|  | **ASD by 4 Years** | | | | | **ASD by 8 Years** | | | | |
|  | **Cumulative Incidence** | | | **Average Age at Diagnosis** | | **Cumulative Incidence** | | | **Average Age at Diagnosis** | |
| **Birth Year** | **ASD Cases** | **Total Births** | **Percent** | **Mean (Yrs)** | **SD (Yrs)** | **ASD Cases** | **Total Births** | **Percent** | **Mean (Yrs)** | **SD (Yrs)** |
| 2018 | 4,236 | 210,431 | 2.01% | 3.36 | 0.49 | --- | --- | --- | --- | --- |
| 2017 | 3,858 | 217,608 | 1.77% | 3.32 | 0.47 | --- | --- | --- | --- | --- |
| 2016 | 3,561 | 221,673 | 1.61% | 3.26 | 0.45 | --- | --- | --- | --- | --- |
| 2015 | 3,127 | 220,554 | 1.42% | 3.29 | 0.46 | --- | --- | --- | --- | --- |
| 2014 | 2,785 | 223,922 | 1.24% | 3.26 | 0.47 | 3,945 | 223,922 | 1.76% | 4.09 | 1.49 |
| 2013 | 2,449 | 214,933 | 1.14% | 3.27 | 0.46 | 3,521 | 214,933 | 1.64% | 4.11 | 1.45 |
| 2012 | 2,324 | 211,337 | 1.10% | 3.27 | 0.46 | 3,371 | 211,337 | 1.60% | 4.14 | 1.49 |
| 2011 | 1,971 | 207,325 | 0.95% | 3.29 | 0.47 | 2,975 | 207,325 | 1.43% | 4.27 | 1.56 |
| 2010 | 1,858 | 202,499 | 0.92% | 3.32 | 0.51 | 2,745 | 202,499 | 1.36% | 4.21 | 1.49 |
| 2009 | 1,566 | 200,201 | 0.78% | 3.29 | 0.48 | 2,392 | 200,201 | 1.19% | 4.24 | 1.50 |
| 2008 | 1,573 | 203,969 | 0.77% | 3.35 | 0.48 | 2,351 | 203,969 | 1.15% | 4.28 | 1.50 |
| 2007 | 1,323 | 202,988 | 0.65% | 3.36 | 0.48 | 2,013 | 202,988 | 0.99% | 4.32 | 1.52 |
| 2006 | 1,269 | 199,325 | 0.64% | 3.40 | 0.49 | 1,848 | 199,325 | 0.93% | 4.24 | 1.46 |
| 2005 | 1,102 | 170,770 | 0.65% | 3.33 | 0.50 | 1,550 | 170,770 | 0.91% | 4.13 | 1.44 |
| 2004 | 1,003 | 170,053 | 0.59% | 3.33 | 0.50 | 1,441 | 170,053 | 0.85% | 4.13 | 1.41 |
| 2003 | 1,047 | 170,900 | 0.61% | 3.34 | 0.50 | 1,525 | 170,900 | 0.89% | 4.16 | 1.40 |
| 2002 | 864 | 165,822 | 0.52% | 3.36 | 0.50 | 1,361 | 165,822 | 0.82% | 4.33 | 1.47 |
| 2001 | 825 | 164,646 | 0.50% | 3.35 | 0.52 | 1,259 | 164,646 | 0.76% | 4.31 | 1.52 |
| 2000 | 766 | 167,557 | 0.46% | 3.40 | 0.51 | 1,149 | 167,557 | 0.69% | 4.32 | 1.52 |
| 1999 | 643 | 160,464 | 0.40% | 3.36 | 0.50 | 991 | 160,464 | 0.62% | 4.28 | 1.46 |
| 1998 | 584 | 159,253 | 0.37% | 3.46 | 0.51 | 912 | 159,253 | 0.57% | 4.35 | 1.41 |
| 1997 | 523 | 156,412 | 0.33% | 3.49 | 0.51 | 828 | 156,412 | 0.53% | 4.40 | 1.42 |
| 1996 | 408 | 153,966 | 0.26% | 3.46 | 0.52 | 738 | 153,966 | 0.48% | 4.65 | 1.56 |
| 1995 | 348 | 153,744 | 0.23% | 3.48 | 0.53 | 627 | 153,744 | 0.41% | 4.66 | 1.55 |
| 1994 | 317 | 156,206 | 0.20% | 3.50 | 0.53 | 572 | 156,206 | 0.37% | 4.72 | 1.62 |
| 1993 | 277 | 158,157 | 0.18% | 3.59 | 0.52 | 493 | 158,157 | 0.31% | 4.69 | 1.50 |
| 1992 | 260 | 161,430 | 0.16% | 3.47 | 0.58 | 491 | 161,430 | 0.30% | 4.66 | 1.51 |
| 1991 | 199 | 160,327 | 0.12% | 3.50 | 0.50 | 368 | 160,327 | 0.23% | 4.75 | 1.57 |
| 1990 | 123 | 157,345 | 0.08% | 4.00 | 0.00 | 253 | 157,345 | 0.16% | 5.13 | 1.36 |

| (Table S5. continued) | | | | | | | | | | |
| --- | --- | --- | --- | --- | --- | --- | --- | --- | --- | --- |
| **Graduate Degree** | | | | | | | | | | |
|  | **ASD by 4 Years** | | | | | **ASD by 8 Years** | | | | |
|  | **Cumulative Incidence** | | | **Average Age at Diagnosis** | | **Cumulative Incidence** | | | **Average Age at Diagnosis** | |
| **Birth Year** | **ASD Cases** | **Total Births** | **Percent** | **Mean (Yrs)** | **SD (Yrs)** | **ASD Cases** | **Total Births** | **Percent** | **Mean (Yrs)** | **SD (Yrs)** |
| 2018 | 695 | 55,740 | 1.25% | 3.36 | 0.50 | --- | --- | --- | --- | --- |
| 2017 | 549 | 55,109 | 1.00% | 3.29 | 0.47 | --- | --- | --- | --- | --- |
| 2016 | 539 | 55,395 | 0.97% | 3.22 | 0.43 | --- | --- | --- | --- | --- |
| 2015 | 430 | 53,111 | 0.81% | 3.31 | 0.49 | --- | --- | --- | --- | --- |
| 2014 | 435 | 52,930 | 0.82% | 3.20 | 0.42 | 617 | 52,930 | 1.17% | 4.06 | 1.51 |
| 2013 | 382 | 49,346 | 0.77% | 3.22 | 0.43 | 568 | 49,346 | 1.15% | 4.19 | 1.57 |
| 2012 | 396 | 48,141 | 0.82% | 3.26 | 0.47 | 579 | 48,141 | 1.20% | 4.13 | 1.45 |
| 2011 | 354 | 45,189 | 0.78% | 3.31 | 0.49 | 510 | 45,189 | 1.13% | 4.16 | 1.46 |
| 2010 | 335 | 44,553 | 0.75% | 3.30 | 0.48 | 493 | 44,553 | 1.11% | 4.28 | 1.61 |
| 2009 | 276 | 43,456 | 0.64% | 3.32 | 0.47 | 399 | 43,456 | 0.92% | 4.21 | 1.54 |
| 2008 | 245 | 42,517 | 0.58% | 3.35 | 0.48 | 360 | 42,517 | 0.85% | 4.24 | 1.50 |
| 2007 | 271 | 42,029 | 0.64% | 3.39 | 0.49 | 390 | 42,029 | 0.93% | 4.14 | 1.34 |
| 2006 | 247 | 41,274 | 0.60% | 3.36 | 0.48 | 353 | 41,274 | 0.86% | 4.19 | 1.46 |
| 2005 | 395 | 61,462 | 0.64% | 3.33 | 0.52 | 526 | 61,462 | 0.86% | 4.00 | 1.36 |
| 2004 | 409 | 60,764 | 0.67% | 3.34 | 0.49 | 544 | 60,764 | 0.90% | 3.98 | 1.28 |
| 2003 | 376 | 58,622 | 0.64% | 3.32 | 0.48 | 507 | 58,622 | 0.86% | 3.98 | 1.30 |
| 2002 | 345 | 54,206 | 0.64% | 3.31 | 0.49 | 500 | 54,206 | 0.92% | 4.09 | 1.34 |
| 2001 | 302 | 51,263 | 0.59% | 3.32 | 0.48 | 435 | 51,263 | 0.85% | 4.10 | 1.36 |
| 2000 | 266 | 49,479 | 0.54% | 3.37 | 0.51 | 383 | 49,479 | 0.77% | 4.19 | 1.45 |
| 1999 | 241 | 45,202 | 0.53% | 3.44 | 0.51 | 340 | 45,202 | 0.75% | 4.23 | 1.43 |
| 1998 | 202 | 43,419 | 0.47% | 3.42 | 0.50 | 302 | 43,419 | 0.70% | 4.21 | 1.34 |
| 1997 | 164 | 40,907 | 0.40% | 3.51 | 0.50 | 250 | 40,907 | 0.61% | 4.30 | 1.32 |
| 1996 | 128 | 38,730 | 0.33% | 3.46 | 0.52 | 207 | 38,730 | 0.53% | 4.48 | 1.51 |
| 1995 | 115 | 38,002 | 0.30% | 3.41 | 0.51 | 189 | 38,002 | 0.50% | 4.51 | 1.57 |
| 1994 | 107 | 37,760 | 0.28% | 3.48 | 0.52 | 191 | 37,760 | 0.51% | 4.63 | 1.53 |
| 1993 | 79 | 38,245 | 0.21% | 3.39 | 0.54 | 134 | 38,245 | 0.35% | 4.54 | 1.61 |
| 1992 | 80 | 38,984 | 0.21% | 3.41 | 0.59 | 132 | 38,984 | 0.34% | 4.51 | 1.64 |
| 1991 | 60 | 39,493 | 0.15% | 3.58 | 0.50 | 113 | 39,493 | 0.29% | 4.68 | 1.40 |
| 1990 | 50 | 39,562 | 0.13% | 4.00 | 0.00 | 95 | 39,562 | 0.24% | 4.96 | 1.26 |
| Notes: SD = Standard Deviation; ASD = Autism Spectrum Disorder; Yrs = Years | | | | | | | | | | |

| **Table S6.** ASD Cumulative Incidence and Average Age at Diagnosis by Birth Year Cohort – Stratified by Neighborhood Socioeconomic Status (nSES) | | | | | | | | | | |
| --- | --- | --- | --- | --- | --- | --- | --- | --- | --- | --- |
| **1 (Low SES)** | | | | | | | | | | |
|  | **ASD by 4 Years** | | | | | **ASD by 8 Years** | | | | |
|  | **Cumulative Incidence** | | | **Average Age at Diagnosis** | | **Cumulative Incidence** | | | **Average Age at Diagnosis** | |
| **Birth Year** | **ASD Cases** | **Total Births** | **Percent** | **Mean (Yrs)** | **SD (Yrs)** | **ASD Cases** | **Total Births** | **Percent** | **Mean (Yrs)** | **SD (Yrs)** |
| 2018 | 2,877 | 115,965 | 2.48% | 3.40 | 0.49 | --- | --- | --- | --- | --- |
| 2017 | 2,492 | 121,742 | 2.05% | 3.39 | 0.49 | --- | --- | --- | --- | --- |
| 2016 | 2,308 | 127,370 | 1.81% | 3.30 | 0.47 | --- | --- | --- | --- | --- |
| 2015 | 1,959 | 129,687 | 1.51% | 3.33 | 0.48 | --- | --- | --- | --- | --- |
| 2014 | 1,705 | 132,705 | 1.28% | 3.30 | 0.47 | 2,573 | 132,705 | 1.94% | 4.22 | 1.49 |
| 2013 | 1,534 | 133,978 | 1.14% | 3.33 | 0.48 | 2,347 | 133,978 | 1.75% | 4.26 | 1.47 |
| 2012 | 1,294 | 138,786 | 0.93% | 3.31 | 0.48 | 1,997 | 138,786 | 1.44% | 4.30 | 1.53 |
| 2011 | 1,166 | 141,110 | 0.83% | 3.31 | 0.49 | 1,944 | 141,110 | 1.38% | 4.43 | 1.58 |
| 2010 | 1,137 | 145,760 | 0.78% | 3.34 | 0.50 | 1,718 | 145,760 | 1.18% | 4.27 | 1.49 |
| 2009 | 995 | 152,085 | 0.65% | 3.33 | 0.49 | 1,597 | 152,085 | 1.05% | 4.38 | 1.56 |
| 2008 | 883 | 160,614 | 0.55% | 3.39 | 0.49 | 1,452 | 160,614 | 0.90% | 4.45 | 1.53 |
| 2007 | 685 | 150,863 | 0.45% | 3.42 | 0.50 | 1,175 | 150,863 | 0.78% | 4.58 | 1.59 |
| 2006 | 673 | 166,591 | 0.40% | 3.44 | 0.50 | 1,110 | 166,591 | 0.67% | 4.48 | 1.51 |
| 2005 | 656 | 160,531 | 0.41% | 3.42 | 0.52 | 1,069 | 160,531 | 0.67% | 4.45 | 1.50 |
| 2004 | 627 | 160,678 | 0.39% | 3.36 | 0.50 | 1,036 | 160,678 | 0.64% | 4.40 | 1.51 |
| 2003 | 595 | 159,924 | 0.37% | 3.43 | 0.51 | 987 | 159,924 | 0.62% | 4.44 | 1.46 |
| 2002 | 488 | 158,260 | 0.31% | 3.47 | 0.52 | 865 | 158,260 | 0.55% | 4.57 | 1.47 |
| 2001 | 417 | 158,562 | 0.26% | 3.48 | 0.52 | 763 | 158,562 | 0.48% | 4.71 | 1.58 |
| 2000 | 378 | 158,760 | 0.24% | 3.47 | 0.51 | 655 | 158,760 | 0.41% | 4.58 | 1.54 |
| 1999 | 294 | 154,716 | 0.19% | 3.54 | 0.53 | 515 | 154,716 | 0.33% | 4.64 | 1.52 |
| 1998 | 297 | 155,891 | 0.19% | 3.55 | 0.51 | 518 | 155,891 | 0.33% | 4.57 | 1.42 |
| 1997 | 213 | 126,132 | 0.17% | 3.52 | 0.52 | 386 | 126,132 | 0.31% | 4.62 | 1.46 |
| 1996 | 166 | 131,498 | 0.13% | 3.44 | 0.52 | 351 | 131,498 | 0.27% | 4.85 | 1.57 |
| 1995 | 158 | 136,166 | 0.12% | 3.44 | 0.50 | 323 | 136,166 | 0.24% | 4.84 | 1.61 |
| 1994 | 135 | 143,389 | 0.09% | 3.56 | 0.51 | 256 | 143,389 | 0.18% | 4.80 | 1.59 |
| 1993 | 110 | 150,981 | 0.07% | 3.51 | 0.54 | 217 | 150,981 | 0.14% | 4.83 | 1.59 |
| 1992 | 104 | 157,086 | 0.07% | 3.51 | 0.57 | 228 | 157,086 | 0.15% | 4.81 | 1.47 |
| 1991 | 96 | 159,903 | 0.06% | 3.48 | 0.50 | 180 | 159,903 | 0.11% | 4.68 | 1.52 |
| 1990 | 75 | 156,272 | 0.05% | 4.00 | 0.00 | 140 | 156,272 | 0.09% | 4.93 | 1.25 |

| (Table S6. continued) | | | | | | | | | | |
| --- | --- | --- | --- | --- | --- | --- | --- | --- | --- | --- |
| **2 (Low-Medium)** | | | | | | | | | | |
|  | **ASD by 4 Years** | | | | | **ASD by 8 Years** | | | | |
|  | **Cumulative Incidence** | | | **Average Age at Diagnosis** | | **Cumulative Incidence** | | | **Average Age at Diagnosis** | |
| **Birth Year** | **ASD Cases** | **Total Births** | **Percent** | **Mean (Yrs)** | **SD (Yrs)** | **ASD Cases** | **Total Births** | **Percent** | **Mean (Yrs)** | **SD (Yrs)** |
| 2018 | 2,263 | 96,890 | 2.34% | 3.37 | 0.49 | --- | --- | --- | --- | --- |
| 2017 | 1,984 | 99,899 | 1.99% | 3.31 | 0.47 | --- | --- | --- | --- | --- |
| 2016 | 1,938 | 104,039 | 1.86% | 3.28 | 0.46 | --- | --- | --- | --- | --- |
| 2015 | 1,722 | 105,063 | 1.64% | 3.30 | 0.47 | --- | --- | --- | --- | --- |
| 2014 | 1,473 | 107,824 | 1.37% | 3.27 | 0.47 | 2,124 | 107,824 | 1.97% | 4.15 | 1.51 |
| 2013 | 1,265 | 106,531 | 1.19% | 3.26 | 0.45 | 1,823 | 106,531 | 1.71% | 4.12 | 1.46 |
| 2012 | 1,191 | 112,730 | 1.06% | 3.29 | 0.48 | 1,806 | 112,730 | 1.60% | 4.22 | 1.48 |
| 2011 | 1,014 | 112,864 | 0.90% | 3.29 | 0.47 | 1,575 | 112,864 | 1.40% | 4.31 | 1.57 |
| 2010 | 980 | 115,306 | 0.85% | 3.32 | 0.50 | 1,487 | 115,306 | 1.29% | 4.25 | 1.49 |
| 2009 | 857 | 119,541 | 0.72% | 3.33 | 0.48 | 1,350 | 119,541 | 1.13% | 4.35 | 1.53 |
| 2008 | 829 | 125,918 | 0.66% | 3.39 | 0.49 | 1,252 | 125,918 | 0.99% | 4.32 | 1.50 |
| 2007 | 684 | 125,021 | 0.55% | 3.41 | 0.49 | 1,100 | 125,021 | 0.88% | 4.43 | 1.50 |
| 2006 | 670 | 128,513 | 0.52% | 3.41 | 0.50 | 1,036 | 128,513 | 0.81% | 4.37 | 1.50 |
| 2005 | 602 | 121,592 | 0.50% | 3.36 | 0.52 | 887 | 121,592 | 0.73% | 4.25 | 1.49 |
| 2004 | 542 | 119,516 | 0.45% | 3.35 | 0.50 | 824 | 119,516 | 0.69% | 4.23 | 1.42 |
| 2003 | 520 | 118,678 | 0.44% | 3.38 | 0.51 | 772 | 118,678 | 0.65% | 4.17 | 1.33 |
| 2002 | 392 | 116,139 | 0.34% | 3.41 | 0.52 | 686 | 116,139 | 0.59% | 4.53 | 1.51 |
| 2001 | 375 | 117,030 | 0.32% | 3.41 | 0.51 | 630 | 117,030 | 0.54% | 4.47 | 1.51 |
| 2000 | 358 | 117,929 | 0.30% | 3.43 | 0.51 | 613 | 117,929 | 0.52% | 4.54 | 1.55 |
| 1999 | 317 | 115,249 | 0.28% | 3.44 | 0.51 | 538 | 115,249 | 0.47% | 4.49 | 1.49 |
| 1998 | 254 | 116,840 | 0.22% | 3.50 | 0.51 | 443 | 116,840 | 0.38% | 4.53 | 1.43 |
| 1997 | 253 | 120,015 | 0.21% | 3.55 | 0.51 | 437 | 120,015 | 0.36% | 4.55 | 1.41 |
| 1996 | 202 | 122,795 | 0.16% | 3.44 | 0.52 | 399 | 122,795 | 0.32% | 4.68 | 1.49 |
| 1995 | 165 | 125,168 | 0.13% | 3.52 | 0.51 | 309 | 125,168 | 0.25% | 4.69 | 1.48 |
| 1994 | 156 | 130,226 | 0.12% | 3.52 | 0.53 | 303 | 130,226 | 0.23% | 4.82 | 1.61 |
| 1993 | 115 | 134,167 | 0.09% | 3.51 | 0.54 | 257 | 134,167 | 0.19% | 4.98 | 1.59 |
| 1992 | 116 | 137,896 | 0.08% | 3.47 | 0.58 | 211 | 137,896 | 0.15% | 4.64 | 1.53 |
| 1991 | 105 | 137,084 | 0.08% | 3.50 | 0.50 | 204 | 137,084 | 0.15% | 4.74 | 1.52 |
| 1990 | 66 | 135,414 | 0.05% | 4.00 | 0.00 | 137 | 135,414 | 0.10% | 5.12 | 1.35 |

| (Table S6. continued) | | | | | | | | | | |
| --- | --- | --- | --- | --- | --- | --- | --- | --- | --- | --- |
| **3 (Medium)** | | | | | | | | | | |
|  | **ASD by 4 Years** | | | | | **ASD by 8 Years** | | | | |
|  | **Cumulative Incidence** | | | **Average Age at Diagnosis** | | **Cumulative Incidence** | | | **Average Age at Diagnosis** | |
| **Birth Year** | **ASD Cases** | **Total Births** | **Percent** | **Mean (Yrs)** | **SD (Yrs)** | **ASD Cases** | **Total Births** | **Percent** | **Mean (Yrs)** | **SD (Yrs)** |
| 2018 | 1,683 | 86,726 | 1.94% | 3.34 | 0.48 | --- | --- | --- | --- | --- |
| 2017 | 1,609 | 90,132 | 1.79% | 3.33 | 0.48 | --- | --- | --- | --- | --- |
| 2016 | 1,569 | 93,656 | 1.68% | 3.25 | 0.44 | --- | --- | --- | --- | --- |
| 2015 | 1,306 | 93,552 | 1.40% | 3.29 | 0.48 | --- | --- | --- | --- | --- |
| 2014 | 1,209 | 95,492 | 1.27% | 3.26 | 0.47 | 1,691 | 95,492 | 1.77% | 4.08 | 1.49 |
| 2013 | 984 | 93,131 | 1.06% | 3.29 | 0.47 | 1,493 | 93,131 | 1.60% | 4.26 | 1.54 |
| 2012 | 953 | 94,744 | 1.01% | 3.27 | 0.46 | 1,429 | 94,744 | 1.51% | 4.21 | 1.50 |
| 2011 | 846 | 94,122 | 0.90% | 3.32 | 0.49 | 1,307 | 94,122 | 1.39% | 4.33 | 1.57 |
| 2010 | 846 | 95,511 | 0.89% | 3.34 | 0.51 | 1,242 | 95,511 | 1.30% | 4.25 | 1.54 |
| 2009 | 695 | 97,434 | 0.71% | 3.30 | 0.48 | 1,074 | 97,434 | 1.10% | 4.28 | 1.51 |
| 2008 | 638 | 102,528 | 0.62% | 3.36 | 0.48 | 1,038 | 102,528 | 1.01% | 4.42 | 1.55 |
| 2007 | 587 | 108,354 | 0.54% | 3.37 | 0.48 | 911 | 108,354 | 0.84% | 4.33 | 1.50 |
| 2006 | 544 | 102,803 | 0.53% | 3.38 | 0.49 | 803 | 102,803 | 0.78% | 4.29 | 1.51 |
| 2005 | 570 | 100,985 | 0.56% | 3.38 | 0.51 | 816 | 100,985 | 0.81% | 4.21 | 1.48 |
| 2004 | 520 | 99,442 | 0.52% | 3.33 | 0.49 | 740 | 99,442 | 0.74% | 4.15 | 1.45 |
| 2003 | 471 | 97,032 | 0.49% | 3.35 | 0.51 | 744 | 97,032 | 0.77% | 4.31 | 1.47 |
| 2002 | 425 | 94,439 | 0.45% | 3.36 | 0.50 | 660 | 94,439 | 0.70% | 4.30 | 1.46 |
| 2001 | 388 | 93,873 | 0.41% | 3.35 | 0.51 | 611 | 93,873 | 0.65% | 4.35 | 1.51 |
| 2000 | 351 | 95,222 | 0.37% | 3.42 | 0.51 | 533 | 95,222 | 0.56% | 4.31 | 1.46 |
| 1999 | 266 | 93,771 | 0.28% | 3.39 | 0.51 | 441 | 93,771 | 0.47% | 4.44 | 1.53 |
| 1998 | 299 | 94,427 | 0.32% | 3.43 | 0.50 | 474 | 94,427 | 0.50% | 4.37 | 1.44 |
| 1997 | 198 | 93,213 | 0.21% | 3.53 | 0.51 | 358 | 93,213 | 0.38% | 4.61 | 1.45 |
| 1996 | 171 | 93,237 | 0.18% | 3.46 | 0.53 | 330 | 93,237 | 0.35% | 4.64 | 1.45 |
| 1995 | 154 | 94,325 | 0.16% | 3.47 | 0.54 | 292 | 94,325 | 0.31% | 4.67 | 1.53 |
| 1994 | 154 | 97,114 | 0.16% | 3.49 | 0.50 | 269 | 97,114 | 0.28% | 4.61 | 1.55 |
| 1993 | 132 | 99,666 | 0.13% | 3.62 | 0.49 | 242 | 99,666 | 0.24% | 4.71 | 1.43 |
| 1992 | 104 | 101,137 | 0.10% | 3.42 | 0.60 | 224 | 101,137 | 0.22% | 4.87 | 1.62 |
| 1991 | 88 | 101,606 | 0.09% | 3.49 | 0.50 | 153 | 101,606 | 0.15% | 4.60 | 1.49 |
| 1990 | 63 | 98,680 | 0.06% | 4.00 | 0.00 | 123 | 98,680 | 0.12% | 5.00 | 1.24 |

| (Table S6. continued) | | | | | | | | | | |
| --- | --- | --- | --- | --- | --- | --- | --- | --- | --- | --- |
| **4 (High-Medium)** | | | | | | | | | | |
|  | **ASD by 4 Years** | | | | | **ASD by 8 Years** | | | | |
|  | **Cumulative Incidence** | | | **Average Age at Diagnosis** | | **Cumulative Incidence** | | | **Average Age at Diagnosis** | |
| **Birth Year** | **ASD Cases** | **Total Births** | **Percent** | **Mean (Yrs)** | **SD (Yrs)** | **ASD Cases** | **Total Births** | **Percent** | **Mean (Yrs)** | **SD (Yrs)** |
| 2018 | 1,370 | 80,268 | 1.71% | 3.32 | 0.48 | --- | --- | --- | --- | --- |
| 2017 | 1,232 | 81,835 | 1.51% | 3.30 | 0.47 | --- | --- | --- | --- | --- |
| 2016 | 1,193 | 84,392 | 1.41% | 3.23 | 0.43 | --- | --- | --- | --- | --- |
| 2015 | 1,062 | 83,992 | 1.26% | 3.27 | 0.46 | --- | --- | --- | --- | --- |
| 2014 | 904 | 85,890 | 1.05% | 3.23 | 0.45 | 1,280 | 85,890 | 1.49% | 4.06 | 1.46 |
| 2013 | 851 | 82,715 | 1.03% | 3.24 | 0.44 | 1,193 | 82,715 | 1.44% | 4.04 | 1.43 |
| 2012 | 858 | 86,004 | 1.00% | 3.27 | 0.47 | 1,251 | 86,004 | 1.45% | 4.16 | 1.48 |
| 2011 | 754 | 85,073 | 0.89% | 3.30 | 0.46 | 1,117 | 85,073 | 1.31% | 4.22 | 1.52 |
| 2010 | 710 | 84,719 | 0.84% | 3.29 | 0.49 | 1,071 | 84,719 | 1.26% | 4.28 | 1.59 |
| 2009 | 616 | 86,140 | 0.72% | 3.30 | 0.48 | 933 | 86,140 | 1.08% | 4.25 | 1.51 |
| 2008 | 661 | 88,595 | 0.75% | 3.33 | 0.47 | 961 | 88,595 | 1.08% | 4.20 | 1.47 |
| 2007 | 589 | 100,189 | 0.59% | 3.34 | 0.47 | 887 | 100,189 | 0.89% | 4.30 | 1.54 |
| 2006 | 507 | 88,193 | 0.57% | 3.40 | 0.50 | 764 | 88,193 | 0.87% | 4.31 | 1.50 |
| 2005 | 496 | 87,311 | 0.57% | 3.32 | 0.48 | 678 | 87,311 | 0.78% | 4.06 | 1.42 |
| 2004 | 469 | 86,775 | 0.54% | 3.32 | 0.49 | 653 | 86,775 | 0.75% | 4.06 | 1.36 |
| 2003 | 474 | 86,016 | 0.55% | 3.29 | 0.50 | 699 | 86,016 | 0.81% | 4.17 | 1.48 |
| 2002 | 393 | 82,601 | 0.48% | 3.34 | 0.49 | 622 | 82,601 | 0.75% | 4.32 | 1.47 |
| 2001 | 338 | 81,336 | 0.42% | 3.33 | 0.49 | 524 | 81,336 | 0.64% | 4.38 | 1.60 |
| 2000 | 365 | 81,892 | 0.45% | 3.39 | 0.51 | 548 | 81,892 | 0.67% | 4.27 | 1.47 |
| 1999 | 280 | 79,740 | 0.35% | 3.40 | 0.50 | 417 | 79,740 | 0.52% | 4.20 | 1.35 |
| 1998 | 262 | 80,561 | 0.33% | 3.46 | 0.50 | 417 | 80,561 | 0.52% | 4.36 | 1.41 |
| 1997 | 260 | 99,333 | 0.26% | 3.53 | 0.51 | 426 | 99,333 | 0.43% | 4.41 | 1.32 |
| 1996 | 197 | 99,704 | 0.20% | 3.48 | 0.50 | 382 | 99,704 | 0.38% | 4.74 | 1.54 |
| 1995 | 191 | 100,534 | 0.19% | 3.42 | 0.52 | 348 | 100,534 | 0.35% | 4.69 | 1.61 |
| 1994 | 170 | 103,443 | 0.16% | 3.50 | 0.52 | 309 | 103,443 | 0.30% | 4.73 | 1.60 |
| 1993 | 143 | 104,759 | 0.14% | 3.59 | 0.55 | 263 | 104,759 | 0.25% | 4.76 | 1.53 |
| 1992 | 116 | 107,917 | 0.11% | 3.47 | 0.57 | 220 | 107,917 | 0.20% | 4.70 | 1.57 |
| 1991 | 95 | 107,601 | 0.09% | 3.60 | 0.49 | 186 | 107,601 | 0.17% | 4.84 | 1.51 |
| 1990 | 62 | 105,892 | 0.06% | 4.00 | 0.00 | 129 | 105,892 | 0.12% | 5.04 | 1.27 |

| (Table S6. continued) | | | | | | | | | | |
| --- | --- | --- | --- | --- | --- | --- | --- | --- | --- | --- |
| **5 (High SES)** | | | | | | | | | | |
|  | **ASD by 4 Years** | | | | | **ASD by 8 Years** | | | | |
|  | **Cumulative Incidence** | | | **Average Age at Diagnosis** | | **Cumulative Incidence** | | | **Average Age at Diagnosis** | |
| **Birth Year** | **ASD Cases** | **Total Births** | **Percent** | **Mean (Yrs)** | **SD (Yrs)** | **ASD Cases** | **Total Births** | **Percent** | **Mean (Yrs)** | **SD (Yrs)** |
| 2018 | 723 | 66,963 | 1.08% | 3.36 | 0.50 | --- | --- | --- | --- | --- |
| 2017 | 605 | 68,504 | 0.88% | 3.26 | 0.45 | --- | --- | --- | --- | --- |
| 2016 | 625 | 69,452 | 0.90% | 3.22 | 0.42 | --- | --- | --- | --- | --- |
| 2015 | 570 | 69,436 | 0.82% | 3.26 | 0.46 | --- | --- | --- | --- | --- |
| 2014 | 540 | 70,533 | 0.77% | 3.2 | 0.42 | 757 | 70,533 | 1.07% | 4.01 | 1.45 |
| 2013 | 488 | 67,830 | 0.72% | 3.21 | 0.43 | 694 | 67,830 | 1.02% | 4.07 | 1.48 |
| 2012 | 522 | 68,764 | 0.76% | 3.25 | 0.46 | 753 | 68,764 | 1.10% | 4.12 | 1.48 |
| 2011 | 475 | 67,314 | 0.71% | 3.31 | 0.46 | 698 | 67,314 | 1.04% | 4.25 | 1.54 |
| 2010 | 477 | 68,406 | 0.70% | 3.35 | 0.50 | 717 | 68,406 | 1.05% | 4.29 | 1.53 |
| 2009 | 399 | 69,645 | 0.57% | 3.29 | 0.48 | 602 | 69,645 | 0.86% | 4.23 | 1.53 |
| 2008 | 400 | 72,218 | 0.55% | 3.34 | 0.48 | 583 | 72,218 | 0.81% | 4.25 | 1.52 |
| 2007 | 449 | 78,485 | 0.57% | 3.34 | 0.48 | 652 | 78,485 | 0.83% | 4.22 | 1.50 |
| 2006 | 437 | 74,420 | 0.59% | 3.37 | 0.48 | 595 | 74,420 | 0.80% | 4.11 | 1.42 |
| 2005 | 454 | 75,921 | 0.60% | 3.27 | 0.49 | 610 | 75,921 | 0.80% | 3.96 | 1.35 |
| 2004 | 456 | 77,003 | 0.59% | 3.34 | 0.50 | 610 | 77,003 | 0.79% | 4.00 | 1.32 |
| 2003 | 444 | 77,630 | 0.57% | 3.34 | 0.48 | 616 | 77,630 | 0.79% | 4.04 | 1.31 |
| 2002 | 403 | 75,655 | 0.53% | 3.32 | 0.49 | 569 | 75,655 | 0.75% | 4.08 | 1.34 |
| 2001 | 384 | 74,699 | 0.51% | 3.3 | 0.50 | 529 | 74,699 | 0.71% | 4.05 | 1.40 |
| 2000 | 324 | 76,519 | 0.42% | 3.38 | 0.50 | 467 | 76,519 | 0.61% | 4.27 | 1.55 |
| 1999 | 337 | 72,950 | 0.46% | 3.37 | 0.49 | 484 | 72,950 | 0.66% | 4.21 | 1.48 |
| 1998 | 282 | 73,156 | 0.39% | 3.39 | 0.52 | 414 | 73,156 | 0.57% | 4.21 | 1.42 |
| 1997 | 246 | 75,569 | 0.33% | 3.52 | 0.50 | 394 | 75,569 | 0.52% | 4.44 | 1.41 |
| 1996 | 181 | 73,206 | 0.25% | 3.48 | 0.51 | 302 | 73,206 | 0.41% | 4.52 | 1.50 |
| 1995 | 147 | 71,326 | 0.21% | 3.51 | 0.52 | 253 | 71,326 | 0.35% | 4.57 | 1.47 |
| 1994 | 127 | 71,942 | 0.18% | 3.54 | 0.53 | 236 | 71,942 | 0.33% | 4.82 | 1.63 |
| 1993 | 119 | 72,826 | 0.16% | 3.45 | 0.59 | 209 | 72,826 | 0.29% | 4.54 | 1.50 |
| 1992 | 100 | 73,444 | 0.14% | 3.45 | 0.59 | 190 | 73,444 | 0.26% | 4.71 | 1.57 |
| 1991 | 83 | 73,776 | 0.11% | 3.54 | 0.50 | 146 | 73,776 | 0.20% | 4.66 | 1.50 |
| 1990 | 60 | 75,608 | 0.08% | 4.00 | 0.00 | 126 | 75,608 | 0.17% | 5.06 | 1.30 |
| Notes: SD = Standard Deviation; ASD = Autism Spectrum Disorder; Yrs = Years; nSES = 1-5 from lowest quintile to highest quintile | | | | | | | | | | |

| **Table S7.** ASD Cumulative Incidence and Average Age at Diagnosis by Birth Year Cohort – Stratified by Region | | | | | | | | | | |
| --- | --- | --- | --- | --- | --- | --- | --- | --- | --- | --- |
| **Superior California** | | | | | | | | | | |
|  | **ASD by 4 Years** | | | | | **ASD by 8 Years** | | | | |
|  | **Cumulative Incidence** | | | **Average Age at Diagnosis** | | **Cumulative Incidence** | | | **Average Age at Diagnosis** | |
| **Birth Year** | **ASD Cases** | **Total Births** | **Percent** | **Mean (Yrs)** | **SD (Yrs)** | **ASD Cases** | **Total Births** | **Percent** | **Mean (Yrs)** | **SD (Yrs)** |
| 2018 | 716 | 36,404 | 1.97% | 3.29 | 0.49 | --- | --- | --- | --- | --- |
| 2017 | 710 | 36,671 | 1.94% | 3.31 | 0.49 | --- | --- | --- | --- | --- |
| 2016 | 738 | 37,639 | 1.96% | 3.19 | 0.43 | --- | --- | --- | --- | --- |
| 2015 | 647 | 37,401 | 1.73% | 3.28 | 0.48 | --- | --- | --- | --- | --- |
| 2014 | 556 | 37,737 | 1.47% | 3.24 | 0.47 | 781 | 37,737 | 2.07% | 4.08 | 1.50 |
| 2013 | 460 | 37,161 | 1.24% | 3.22 | 0.45 | 683 | 37,161 | 1.84% | 4.14 | 1.50 |
| 2012 | 439 | 37,290 | 1.18% | 3.24 | 0.52 | 661 | 37,290 | 1.77% | 4.22 | 1.56 |
| 2011 | 380 | 37,945 | 1.00% | 3.23 | 0.47 | 576 | 37,945 | 1.52% | 4.24 | 1.59 |
| 2010 | 399 | 38,490 | 1.04% | 3.22 | 0.53 | 555 | 38,490 | 1.44% | 4.05 | 1.52 |
| 2009 | 335 | 38,764 | 0.86% | 3.23 | 0.45 | 508 | 38,764 | 1.31% | 4.24 | 1.61 |
| 2008 | 303 | 40,547 | 0.75% | 3.38 | 0.49 | 453 | 40,547 | 1.12% | 4.29 | 1.49 |
| 2007 | 236 | 41,303 | 0.57% | 3.47 | 0.50 | 391 | 41,303 | 0.95% | 4.50 | 1.48 |
| 2006 | 227 | 41,678 | 0.54% | 3.48 | 0.52 | 361 | 41,678 | 0.87% | 4.50 | 1.57 |
| 2005 | 231 | 40,136 | 0.58% | 3.36 | 0.54 | 329 | 40,136 | 0.82% | 4.22 | 1.54 |
| 2004 | 196 | 39,078 | 0.50% | 3.34 | 0.52 | 283 | 39,078 | 0.72% | 4.17 | 1.44 |
| 2003 | 160 | 38,585 | 0.41% | 3.30 | 0.47 | 285 | 38,585 | 0.74% | 4.46 | 1.54 |
| 2002 | 121 | 36,582 | 0.33% | 3.37 | 0.49 | 239 | 36,582 | 0.65% | 4.74 | 1.61 |
| 2001 | 124 | 35,550 | 0.35% | 3.38 | 0.49 | 209 | 35,550 | 0.59% | 4.58 | 1.67 |
| 2000 | 84 | 34,371 | 0.24% | 3.43 | 0.50 | 140 | 34,371 | 0.41% | 4.64 | 1.71 |
| 1999 | 59 | 33,670 | 0.18% | 3.54 | 0.50 | 104 | 33,670 | 0.31% | 4.76 | 1.64 |
| 1998 | 58 | 33,686 | 0.17% | 3.43 | 0.50 | 100 | 33,686 | 0.30% | 4.55 | 1.57 |
| 1997 | 57 | 33,213 | 0.17% | 3.54 | 0.50 | 89 | 33,213 | 0.27% | 4.36 | 1.32 |
| 1996 | 38 | 34,217 | 0.11% | 3.58 | 0.50 | 76 | 34,217 | 0.22% | 4.97 | 1.68 |
| 1995 | 27 | 35,398 | 0.08% | 3.59 | 0.50 | 50 | 35,398 | 0.14% | 4.84 | 1.57 |
| 1994 | 36 | 35,739 | 0.10% | 3.36 | 0.49 | 54 | 35,739 | 0.15% | 4.28 | 1.48 |
| 1993 | 29 | 36,711 | 0.08% | 3.48 | 0.51 | 50 | 36,711 | 0.14% | 4.50 | 1.47 |
| 1992 | 20 | 37,189 | 0.05% | 3.55 | 0.51 | 30 | 37,189 | 0.08% | 4.23 | 1.17 |
| 1991 | 23 | 37,348 | 0.06% | 3.39 | 0.50 | 40 | 37,348 | 0.11% | 4.35 | 1.33 |
| 1990 | 12 | 36,881 | 0.03% | 4.00 | 0.00 | 26 | 36,881 | 0.07% | 5.19 | 1.47 |

| (Table S7. continued) | | | | | | | | | | |
| --- | --- | --- | --- | --- | --- | --- | --- | --- | --- | --- |
| **North Coast** | | | | | | | | | | |
|  | **ASD by 4 Years** | | | | | **ASD by 8 Years** | | | | |
|  | **Cumulative Incidence** | | | **Average Age at Diagnosis** | | **Cumulative Incidence** | | | **Average Age at Diagnosis** | |
| **Birth Year** | **ASD Cases** | **Total Births** | **Percent** | **Mean (Yrs)** | **SD (Yrs)** | **ASD Cases** | **Total Births** | **Percent** | **Mean (Yrs)** | **SD (Yrs)** |
| 2018 | 190 | 9,092 | 2.09% | 3.31 | 0.47 | --- | --- | --- | --- | --- |
| 2017 | 153 | 9,399 | 1.63% | 3.31 | 0.46 | --- | --- | --- | --- | --- |
| 2016 | 131 | 9,999 | 1.31% | 3.27 | 0.48 | --- | --- | --- | --- | --- |
| 2015 | 149 | 10,033 | 1.49% | 3.34 | 0.48 | --- | --- | --- | --- | --- |
| 2014 | 129 | 10,111 | 1.28% | 3.35 | 0.49 | 188 | 10,111 | 1.86% | 4.21 | 1.49 |
| 2013 | 116 | 10,170 | 1.14% | 3.34 | 0.47 | 168 | 10,170 | 1.65% | 4.23 | 1.52 |
| 2012 | 102 | 10,340 | 0.99% | 3.44 | 0.50 | 145 | 10,340 | 1.40% | 4.12 | 1.22 |
| 2011 | 74 | 10,381 | 0.71% | 3.55 | 0.50 | 141 | 10,381 | 1.36% | 4.80 | 1.56 |
| 2010 | 66 | 10,727 | 0.62% | 3.45 | 0.53 | 122 | 10,727 | 1.14% | 4.74 | 1.63 |
| 2009 | 43 | 11,095 | 0.39% | 3.42 | 0.50 | 73 | 11,095 | 0.66% | 4.55 | 1.61 |
| 2008 | 56 | 11,288 | 0.50% | 3.48 | 0.50 | 92 | 11,288 | 0.82% | 4.48 | 1.46 |
| 2007 | 64 | 11,436 | 0.56% | 3.39 | 0.49 | 95 | 11,436 | 0.83% | 4.25 | 1.42 |
| 2006 | 60 | 11,544 | 0.52% | 3.65 | 0.48 | 83 | 11,544 | 0.72% | 4.31 | 1.30 |
| 2005 | 51 | 11,121 | 0.46% | 3.29 | 0.50 | 85 | 11,121 | 0.76% | 4.34 | 1.52 |
| 2004 | 49 | 11,259 | 0.44% | 3.47 | 0.50 | 75 | 11,259 | 0.67% | 4.36 | 1.45 |
| 2003 | 56 | 11,131 | 0.50% | 3.29 | 0.53 | 98 | 11,131 | 0.88% | 4.50 | 1.63 |
| 2002 | 40 | 10,756 | 0.37% | 3.25 | 0.49 | 77 | 10,756 | 0.72% | 4.49 | 1.52 |
| 2001 | 35 | 10,824 | 0.32% | 3.31 | 0.53 | 58 | 10,824 | 0.54% | 4.38 | 1.53 |
| 2000 | 21 | 10,585 | 0.20% | 3.29 | 0.46 | 46 | 10,585 | 0.43% | 4.76 | 1.64 |
| 1999 | 27 | 10,340 | 0.26% | 3.48 | 0.51 | 44 | 10,340 | 0.43% | 4.25 | 1.12 |
| 1998 | 17 | 10,504 | 0.16% | 3.41 | 0.62 | 34 | 10,504 | 0.32% | 4.53 | 1.42 |
| 1997 | 15 | 10,747 | 0.14% | 3.53 | 0.52 | 25 | 10,747 | 0.23% | 4.64 | 1.63 |
| 1996 | 14 | 10,988 | 0.13% | 3.57 | 0.51 | 31 | 10,988 | 0.28% | 4.84 | 1.44 |
| 1995 | 8 | 11,069 | 0.07% | 3.38 | 0.52 | 23 | 11,069 | 0.21% | 5.09 | 1.59 |
| 1994 | 11 | 11,363 | 0.10% | 3.27 | 0.47 | 21 | 11,363 | 0.18% | 4.81 | 1.89 |
| 1993 | 10 | 11,548 | 0.09% | 3.60 | 0.52 | 21 | 11,548 | 0.18% | 4.90 | 1.48 |
| 1992 | 16 | 11,837 | 0.14% | 3.31 | 0.60 | 24 | 11,837 | 0.20% | 4.00 | 1.18 |
| 1991 | 4 | 12,304 | 0.03% | 3.50 | 0.58 | 7 | 12,304 | 0.06% | 4.43 | 1.40 |
| 1990 | 3 | 12,430 | 0.02% | 4.00 | 0.00 | 9 | 12,430 | 0.07% | 5.44 | 1.33 |

| (Table S7. continued) | | | | | | | | | | |
| --- | --- | --- | --- | --- | --- | --- | --- | --- | --- | --- |
| **San Francisco Bay Area** | | | | | | | | | | |
|  | **ASD by 4 Years** | | | | | **ASD by 8 Years** | | | | |
|  | **Cumulative Incidence** | | | **Average Age at Diagnosis** | | **Cumulative Incidence** | | | **Average Age at Diagnosis** | |
| **Birth Year** | **ASD Cases** | **Total Births** | **Percent** | **Mean (Yrs)** | **SD (Yrs)** | **ASD Cases** | **Total Births** | **Percent** | **Mean (Yrs)** | **SD (Yrs)** |
| 2018 | 948 | 75,635 | 1.25% | 3.39 | 0.49 | --- | --- | --- | --- | --- |
| 2017 | 853 | 77,912 | 1.09% | 3.29 | 0.46 | --- | --- | --- | --- | --- |
| 2016 | 917 | 80,243 | 1.14% | 3.21 | 0.41 | --- | --- | --- | --- | --- |
| 2015 | 813 | 80,592 | 1.01% | 3.26 | 0.45 | --- | --- | --- | --- | --- |
| 2014 | 768 | 81,529 | 0.94% | 3.23 | 0.45 | 1,084 | 81,529 | 1.33% | 4.05 | 1.46 |
| 2013 | 652 | 79,615 | 0.82% | 3.25 | 0.47 | 955 | 79,615 | 1.20% | 4.12 | 1.45 |
| 2012 | 580 | 81,235 | 0.71% | 3.26 | 0.46 | 869 | 81,235 | 1.07% | 4.18 | 1.50 |
| 2011 | 544 | 79,950 | 0.68% | 3.29 | 0.47 | 829 | 79,950 | 1.04% | 4.30 | 1.58 |
| 2010 | 508 | 80,989 | 0.63% | 3.35 | 0.52 | 773 | 80,989 | 0.95% | 4.38 | 1.64 |
| 2009 | 425 | 84,133 | 0.51% | 3.32 | 0.49 | 690 | 84,133 | 0.82% | 4.42 | 1.58 |
| 2008 | 413 | 87,845 | 0.47% | 3.29 | 0.45 | 657 | 87,845 | 0.75% | 4.36 | 1.59 |
| 2007 | 386 | 89,870 | 0.43% | 3.35 | 0.48 | 589 | 89,870 | 0.66% | 4.31 | 1.52 |
| 2006 | 380 | 88,338 | 0.43% | 3.41 | 0.50 | 546 | 88,338 | 0.62% | 4.24 | 1.47 |
| 2005 | 340 | 87,209 | 0.39% | 3.28 | 0.51 | 504 | 87,209 | 0.58% | 4.15 | 1.44 |
| 2004 | 325 | 87,761 | 0.37% | 3.37 | 0.50 | 478 | 87,761 | 0.54% | 4.25 | 1.47 |
| 2003 | 356 | 89,066 | 0.40% | 3.31 | 0.49 | 538 | 89,066 | 0.60% | 4.20 | 1.44 |
| 2002 | 315 | 88,943 | 0.35% | 3.38 | 0.54 | 501 | 88,943 | 0.56% | 4.37 | 1.48 |
| 2001 | 303 | 89,102 | 0.34% | 3.34 | 0.55 | 460 | 89,102 | 0.52% | 4.27 | 1.50 |
| 2000 | 271 | 90,652 | 0.30% | 3.42 | 0.51 | 430 | 90,652 | 0.47% | 4.43 | 1.54 |
| 1999 | 210 | 85,541 | 0.25% | 3.44 | 0.53 | 349 | 85,541 | 0.41% | 4.54 | 1.58 |
| 1998 | 200 | 86,428 | 0.23% | 3.45 | 0.50 | 361 | 86,428 | 0.42% | 4.53 | 1.43 |
| 1997 | 132 | 85,135 | 0.16% | 3.64 | 0.48 | 251 | 85,135 | 0.29% | 4.78 | 1.47 |
| 1996 | 122 | 85,533 | 0.14% | 3.43 | 0.55 | 240 | 85,533 | 0.28% | 4.71 | 1.54 |
| 1995 | 112 | 85,216 | 0.13% | 3.45 | 0.52 | 216 | 85,216 | 0.25% | 4.78 | 1.60 |
| 1994 | 114 | 88,041 | 0.13% | 3.53 | 0.54 | 199 | 88,041 | 0.23% | 4.68 | 1.61 |
| 1993 | 87 | 89,325 | 0.10% | 3.57 | 0.50 | 167 | 89,325 | 0.19% | 4.69 | 1.45 |
| 1992 | 64 | 91,868 | 0.07% | 3.48 | 0.56 | 155 | 91,868 | 0.17% | 5.01 | 1.55 |
| 1991 | 68 | 93,927 | 0.07% | 3.56 | 0.50 | 123 | 93,927 | 0.13% | 4.70 | 1.50 |
| 1990 | 43 | 94,953 | 0.05% | 4.00 | 0.00 | 97 | 94,953 | 0.10% | 5.16 | 1.31 |

| (Table S7. continued) | | | | | | | | | | |
| --- | --- | --- | --- | --- | --- | --- | --- | --- | --- | --- |
| **Northern San Joaquin Valley** | | | | | | | | | | |
|  | **ASD by 4 Years** | | | | | **ASD by 8 Years** | | | | |
|  | **Cumulative Incidence** | | | **Average Age at Diagnosis** | | **Cumulative Incidence** | | | **Average Age at Diagnosis** | |
| **Birth Year** | **ASD Cases** | **Total Births** | **Percent** | **Mean (Yrs)** | **SD (Yrs)** | **ASD Cases** | **Total Births** | **Percent** | **Mean (Yrs)** | **SD (Yrs)** |
| 2018 | 724 | 24,520 | 2.95% | 3.35 | 0.48 | --- | --- | --- | --- | --- |
| 2017 | 586 | 25,017 | 2.34% | 3.31 | 0.46 | --- | --- | --- | --- | --- |
| 2016 | 501 | 25,842 | 1.94% | 3.30 | 0.46 | --- | --- | --- | --- | --- |
| 2015 | 365 | 25,328 | 1.44% | 3.36 | 0.48 | --- | --- | --- | --- | --- |
| 2014 | 402 | 25,314 | 1.59% | 3.30 | 0.46 | 549 | 25,314 | 2.17% | 4.00 | 1.36 |
| 2013 | 317 | 25,079 | 1.26% | 3.26 | 0.44 | 428 | 25,079 | 1.71% | 3.98 | 1.39 |
| 2012 | 303 | 25,543 | 1.19% | 3.27 | 0.45 | 420 | 25,543 | 1.64% | 4.00 | 1.34 |
| 2011 | 277 | 26,003 | 1.07% | 3.32 | 0.47 | 404 | 26,003 | 1.55% | 4.18 | 1.47 |
| 2010 | 226 | 26,460 | 0.85% | 3.29 | 0.45 | 339 | 26,460 | 1.28% | 4.17 | 1.44 |
| 2009 | 223 | 26,887 | 0.83% | 3.29 | 0.45 | 312 | 26,887 | 1.16% | 4.07 | 1.42 |
| 2008 | 228 | 27,913 | 0.82% | 3.29 | 0.45 | 300 | 27,913 | 1.07% | 3.96 | 1.35 |
| 2007 | 190 | 29,035 | 0.65% | 3.27 | 0.45 | 253 | 29,035 | 0.87% | 4.00 | 1.44 |
| 2006 | 197 | 29,331 | 0.67% | 3.34 | 0.49 | 264 | 29,331 | 0.90% | 3.95 | 1.22 |
| 2005 | 172 | 28,062 | 0.61% | 3.30 | 0.55 | 237 | 28,062 | 0.84% | 4.02 | 1.36 |
| 2004 | 121 | 27,073 | 0.45% | 3.32 | 0.52 | 174 | 27,073 | 0.64% | 4.05 | 1.28 |
| 2003 | 108 | 26,350 | 0.41% | 3.25 | 0.60 | 162 | 26,350 | 0.61% | 4.16 | 1.51 |
| 2002 | 91 | 25,515 | 0.36% | 3.35 | 0.52 | 156 | 25,515 | 0.61% | 4.42 | 1.47 |
| 2001 | 99 | 24,815 | 0.40% | 3.32 | 0.57 | 159 | 24,815 | 0.64% | 4.34 | 1.53 |
| 2000 | 78 | 24,044 | 0.32% | 3.47 | 0.50 | 125 | 24,044 | 0.52% | 4.42 | 1.44 |
| 1999 | 57 | 22,798 | 0.25% | 3.33 | 0.61 | 97 | 22,798 | 0.43% | 4.48 | 1.61 |
| 1998 | 54 | 22,435 | 0.24% | 3.35 | 0.48 | 84 | 22,435 | 0.37% | 4.36 | 1.56 |
| 1997 | 40 | 22,346 | 0.18% | 3.43 | 0.59 | 70 | 22,346 | 0.31% | 4.50 | 1.43 |
| 1996 | 37 | 22,877 | 0.16% | 3.49 | 0.51 | 61 | 22,877 | 0.27% | 4.57 | 1.53 |
| 1995 | 34 | 23,541 | 0.14% | 3.32 | 0.47 | 60 | 23,541 | 0.25% | 4.63 | 1.76 |
| 1994 | 26 | 24,285 | 0.11% | 3.50 | 0.58 | 45 | 24,285 | 0.19% | 4.73 | 1.72 |
| 1993 | 21 | 24,732 | 0.08% | 3.76 | 0.44 | 35 | 24,732 | 0.14% | 4.86 | 1.57 |
| 1992 | 11 | 24,809 | 0.04% | 3.45 | 0.52 | 31 | 24,809 | 0.12% | 5.39 | 1.78 |
| 1991 | 8 | 25,302 | 0.03% | 3.63 | 0.52 | 18 | 25,302 | 0.07% | 5.06 | 1.47 |
| 1990 | 7 | 25,546 | 0.03% | 4.00 | 0.00 | 18 | 25,546 | 0.07% | 5.28 | 1.45 |

| (Table S7. continued) | | | | | | | | | | |
| --- | --- | --- | --- | --- | --- | --- | --- | --- | --- | --- |
| **Central Coast** | | | | | | | | | | |
|  | **ASD by 4 Years** | | | | | **ASD by 8 Years** | | | | |
|  | **Cumulative Incidence** | | | **Average Age at Diagnosis** | | **Cumulative Incidence** | | | **Average Age at Diagnosis** | |
| **Birth Year** | **ASD Cases** | **Total Births** | **Percent** | **Mean (Yrs)** | **SD (Yrs)** | **ASD Cases** | **Total Births** | **Percent** | **Mean (Yrs)** | **SD (Yrs)** |
| 2018 | 375 | 25,836 | 1.45% | 3.23 | 0.42 | --- | --- | --- | --- | --- |
| 2017 | 311 | 26,477 | 1.17% | 3.25 | 0.44 | --- | --- | --- | --- | --- |
| 2016 | 344 | 27,371 | 1.26% | 3.15 | 0.36 | --- | --- | --- | --- | --- |
| 2015 | 297 | 28,253 | 1.05% | 3.21 | 0.43 | --- | --- | --- | --- | --- |
| 2014 | 286 | 28,984 | 0.99% | 3.18 | 0.38 | 396 | 28,984 | 1.37% | 4.03 | 1.53 |
| 2013 | 286 | 28,883 | 0.99% | 3.25 | 0.44 | 376 | 28,883 | 1.30% | 3.93 | 1.39 |
| 2012 | 210 | 29,056 | 0.72% | 3.27 | 0.45 | 322 | 29,056 | 1.11% | 4.21 | 1.47 |
| 2011 | 193 | 29,811 | 0.65% | 3.29 | 0.46 | 286 | 29,811 | 0.96% | 4.19 | 1.50 |
| 2010 | 195 | 30,397 | 0.64% | 3.26 | 0.47 | 300 | 30,397 | 0.99% | 4.28 | 1.59 |
| 2009 | 167 | 31,052 | 0.54% | 3.26 | 0.44 | 261 | 31,052 | 0.84% | 4.33 | 1.62 |
| 2008 | 173 | 32,790 | 0.53% | 3.36 | 0.48 | 275 | 32,790 | 0.84% | 4.37 | 1.52 |
| 2007 | 152 | 33,128 | 0.46% | 3.36 | 0.48 | 217 | 33,128 | 0.66% | 4.18 | 1.43 |
| 2006 | 125 | 33,158 | 0.38% | 3.38 | 0.49 | 198 | 33,158 | 0.60% | 4.37 | 1.52 |
| 2005 | 106 | 32,675 | 0.32% | 3.42 | 0.51 | 179 | 32,675 | 0.55% | 4.61 | 1.67 |
| 2004 | 114 | 32,457 | 0.35% | 3.31 | 0.46 | 168 | 32,457 | 0.52% | 4.22 | 1.51 |
| 2003 | 117 | 32,088 | 0.36% | 3.41 | 0.49 | 182 | 32,088 | 0.57% | 4.35 | 1.47 |
| 2002 | 77 | 31,051 | 0.25% | 3.36 | 0.48 | 131 | 31,051 | 0.42% | 4.46 | 1.53 |
| 2001 | 85 | 31,054 | 0.27% | 3.34 | 0.48 | 140 | 31,054 | 0.45% | 4.49 | 1.63 |
| 2000 | 101 | 31,243 | 0.32% | 3.37 | 0.50 | 142 | 31,243 | 0.45% | 4.09 | 1.37 |
| 1999 | 79 | 30,395 | 0.26% | 3.48 | 0.50 | 116 | 30,395 | 0.38% | 4.28 | 1.43 |
| 1998 | 79 | 30,820 | 0.26% | 3.58 | 0.50 | 123 | 30,820 | 0.40% | 4.37 | 1.29 |
| 1997 | 64 | 30,573 | 0.21% | 3.53 | 0.50 | 104 | 30,573 | 0.34% | 4.47 | 1.43 |
| 1996 | 37 | 30,736 | 0.12% | 3.49 | 0.51 | 80 | 30,736 | 0.26% | 4.84 | 1.50 |
| 1995 | 47 | 31,277 | 0.15% | 3.40 | 0.54 | 85 | 31,277 | 0.27% | 4.66 | 1.64 |
| 1994 | 31 | 32,173 | 0.10% | 3.74 | 0.44 | 67 | 32,173 | 0.21% | 5.19 | 1.64 |
| 1993 | 27 | 32,953 | 0.08% | 3.63 | 0.74 | 58 | 32,953 | 0.18% | 4.93 | 1.52 |
| 1992 | 26 | 33,704 | 0.08% | 3.54 | 0.65 | 51 | 33,704 | 0.15% | 4.69 | 1.46 |
| 1991 | 24 | 34,352 | 0.07% | 3.50 | 0.51 | 40 | 34,352 | 0.12% | 4.55 | 1.48 |
| 1990 | 13 | 34,401 | 0.04% | 4.00 | 0.00 | 29 | 34,401 | 0.08% | 5.34 | 1.56 |

| (Table S7. continued) | | | | | | | | | | |
| --- | --- | --- | --- | --- | --- | --- | --- | --- | --- | --- |
| **Southern San Joaquin Valley** | | | | | | | | | | |
|  | **ASD by 4 Years** | | | | | **ASD by 8 Years** | | | | |
|  | **Cumulative Incidence** | | | **Average Age at Diagnosis** | | **Cumulative Incidence** | | | **Average Age at Diagnosis** | |
| **Birth Year** | **ASD Cases** | **Total Births** | **Percent** | **Mean (Yrs)** | **SD (Yrs)** | **ASD Cases** | **Total Births** | **Percent** | **Mean (Yrs)** | **SD (Yrs)** |
| 2018 | 835 | 36,628 | 2.28% | 3.59 | 0.49 | --- | --- | --- | --- | --- |
| 2017 | 625 | 37,349 | 1.67% | 3.66 | 0.48 | --- | --- | --- | --- | --- |
| 2016 | 592 | 38,257 | 1.55% | 3.47 | 0.50 | --- | --- | --- | --- | --- |
| 2015 | 495 | 38,812 | 1.28% | 3.49 | 0.50 | --- | --- | --- | --- | --- |
| 2014 | 406 | 39,925 | 1.02% | 3.46 | 0.50 | 636 | 39,925 | 1.59% | 4.40 | 1.48 |
| 2013 | 369 | 39,891 | 0.93% | 3.47 | 0.50 | 544 | 39,891 | 1.36% | 4.25 | 1.33 |
| 2012 | 353 | 40,784 | 0.87% | 3.38 | 0.49 | 518 | 40,784 | 1.27% | 4.21 | 1.39 |
| 2011 | 304 | 41,007 | 0.74% | 3.41 | 0.49 | 488 | 41,007 | 1.19% | 4.40 | 1.48 |
| 2010 | 271 | 41,538 | 0.65% | 3.45 | 0.51 | 417 | 41,538 | 1.00% | 4.37 | 1.47 |
| 2009 | 207 | 42,209 | 0.49% | 3.48 | 0.50 | 350 | 42,209 | 0.83% | 4.54 | 1.49 |
| 2008 | 208 | 43,376 | 0.48% | 3.48 | 0.50 | 353 | 43,376 | 0.81% | 4.57 | 1.54 |
| 2007 | 153 | 43,918 | 0.35% | 3.56 | 0.50 | 270 | 43,918 | 0.61% | 4.69 | 1.51 |
| 2006 | 133 | 43,061 | 0.31% | 3.53 | 0.52 | 251 | 43,061 | 0.58% | 4.80 | 1.61 |
| 2005 | 140 | 40,764 | 0.34% | 3.47 | 0.50 | 230 | 40,764 | 0.56% | 4.47 | 1.45 |
| 2004 | 123 | 40,047 | 0.31% | 3.49 | 0.50 | 212 | 40,047 | 0.53% | 4.54 | 1.48 |
| 2003 | 111 | 38,366 | 0.29% | 3.61 | 0.49 | 206 | 38,366 | 0.54% | 4.66 | 1.35 |
| 2002 | 76 | 36,734 | 0.21% | 3.45 | 0.50 | 149 | 36,734 | 0.41% | 4.75 | 1.53 |
| 2001 | 69 | 35,488 | 0.19% | 3.55 | 0.50 | 138 | 35,488 | 0.39% | 4.94 | 1.62 |
| 2000 | 69 | 35,465 | 0.19% | 3.67 | 0.47 | 136 | 35,465 | 0.38% | 4.90 | 1.55 |
| 1999 | 50 | 34,375 | 0.15% | 3.58 | 0.50 | 90 | 34,375 | 0.26% | 4.50 | 1.20 |
| 1998 | 36 | 35,135 | 0.10% | 3.47 | 0.51 | 75 | 35,135 | 0.21% | 4.91 | 1.60 |
| 1997 | 35 | 34,475 | 0.10% | 3.51 | 0.51 | 76 | 34,475 | 0.22% | 4.88 | 1.55 |
| 1996 | 26 | 35,680 | 0.07% | 3.69 | 0.47 | 64 | 35,680 | 0.18% | 5.28 | 1.60 |
| 1995 | 22 | 36,323 | 0.06% | 3.50 | 0.51 | 63 | 36,323 | 0.17% | 5.29 | 1.63 |
| 1994 | 18 | 37,932 | 0.05% | 3.78 | 0.43 | 40 | 37,932 | 0.11% | 5.40 | 1.72 |
| 1993 | 9 | 38,410 | 0.02% | 3.56 | 0.53 | 39 | 38,410 | 0.10% | 5.77 | 1.58 |
| 1992 | 16 | 39,062 | 0.04% | 3.38 | 0.50 | 35 | 39,062 | 0.09% | 4.63 | 1.33 |
| 1991 | 14 | 38,921 | 0.04% | 3.71 | 0.47 | 33 | 38,921 | 0.08% | 5.03 | 1.33 |
| 1990 | 6 | 37,758 | 0.02% | 4.00 | 0.00 | 12 | 37,758 | 0.03% | 5.17 | 1.40 |

| (Table S7. continued) | | | | | | | | | | |
| --- | --- | --- | --- | --- | --- | --- | --- | --- | --- | --- |
| **Inland Empire** | | | | | | | | | | |
|  | **ASD by 4 Years** | | | | | **ASD by 8 Years** | | | | |
|  | **Cumulative Incidence** | | | **Average Age at Diagnosis** | | **Cumulative Incidence** | | | **Average Age at Diagnosis** | |
| **Birth Year** | **ASD Cases** | **Total Births** | **Percent** | **Mean (Yrs)** | **SD (Yrs)** | **ASD Cases** | **Total Births** | **Percent** | **Mean (Yrs)** | **SD (Yrs)** |
| 2018 | 677 | 57,648 | 1.17% | 3.37 | 0.48 | --- | --- | --- | --- | --- |
| 2017 | 740 | 59,334 | 1.25% | 3.35 | 0.48 | --- | --- | --- | --- | --- |
| 2016 | 601 | 61,565 | 0.98% | 3.24 | 0.44 | --- | --- | --- | --- | --- |
| 2015 | 544 | 60,958 | 0.89% | 3.24 | 0.43 | --- | --- | --- | --- | --- |
| 2014 | 443 | 61,489 | 0.72% | 3.20 | 0.46 | 669 | 61,489 | 1.09% | 4.12 | 1.47 |
| 2013 | 425 | 59,953 | 0.71% | 3.25 | 0.45 | 654 | 59,953 | 1.09% | 4.26 | 1.57 |
| 2012 | 426 | 60,750 | 0.70% | 3.26 | 0.47 | 661 | 60,750 | 1.09% | 4.28 | 1.55 |
| 2011 | 311 | 61,052 | 0.51% | 3.30 | 0.48 | 541 | 61,052 | 0.89% | 4.49 | 1.58 |
| 2010 | 288 | 62,046 | 0.46% | 3.32 | 0.49 | 489 | 62,046 | 0.79% | 4.50 | 1.61 |
| 2009 | 255 | 63,369 | 0.40% | 3.35 | 0.49 | 422 | 63,369 | 0.67% | 4.45 | 1.58 |
| 2008 | 226 | 66,467 | 0.34% | 3.40 | 0.49 | 384 | 66,467 | 0.58% | 4.53 | 1.56 |
| 2007 | 223 | 69,544 | 0.32% | 3.38 | 0.50 | 372 | 69,544 | 0.53% | 4.53 | 1.63 |
| 2006 | 215 | 68,256 | 0.31% | 3.40 | 0.49 | 342 | 68,256 | 0.50% | 4.46 | 1.59 |
| 2005 | 196 | 64,484 | 0.30% | 3.40 | 0.52 | 292 | 64,484 | 0.45% | 4.34 | 1.54 |
| 2004 | 177 | 61,445 | 0.29% | 3.31 | 0.54 | 277 | 61,445 | 0.45% | 4.30 | 1.53 |
| 2003 | 154 | 58,802 | 0.26% | 3.32 | 0.52 | 238 | 58,802 | 0.40% | 4.32 | 1.55 |
| 2002 | 133 | 56,268 | 0.24% | 3.38 | 0.52 | 218 | 56,268 | 0.39% | 4.42 | 1.53 |
| 2001 | 113 | 54,557 | 0.21% | 3.44 | 0.50 | 177 | 54,557 | 0.32% | 4.49 | 1.61 |
| 2000 | 105 | 53,533 | 0.20% | 3.35 | 0.54 | 166 | 53,533 | 0.31% | 4.33 | 1.51 |
| 1999 | 60 | 51,907 | 0.12% | 3.42 | 0.50 | 139 | 51,907 | 0.27% | 4.91 | 1.61 |
| 1998 | 69 | 51,528 | 0.13% | 3.38 | 0.55 | 124 | 51,528 | 0.24% | 4.63 | 1.61 |
| 1997 | 52 | 48,504 | 0.11% | 3.31 | 0.54 | 99 | 48,504 | 0.20% | 4.63 | 1.64 |
| 1996 | 57 | 49,603 | 0.11% | 3.46 | 0.54 | 110 | 49,603 | 0.22% | 4.62 | 1.42 |
| 1995 | 38 | 50,858 | 0.07% | 3.53 | 0.51 | 95 | 50,858 | 0.19% | 5.11 | 1.57 |
| 1994 | 49 | 53,234 | 0.09% | 3.53 | 0.54 | 87 | 53,234 | 0.16% | 4.75 | 1.62 |
| 1993 | 29 | 54,197 | 0.05% | 3.38 | 0.49 | 66 | 54,197 | 0.12% | 4.95 | 1.65 |
| 1992 | 29 | 54,720 | 0.05% | 3.10 | 0.62 | 66 | 54,720 | 0.12% | 4.79 | 1.77 |
| 1991 | 24 | 50,406 | 0.05% | 3.54 | 0.51 | 41 | 50,406 | 0.08% | 4.90 | 1.79 |
| 1990 | 17 | 47,069 | 0.04% | 4.00 | 0.00 | 29 | 47,069 | 0.06% | 4.83 | 1.17 |

| (Table S7. continued) | | | | | | | | | | |
| --- | --- | --- | --- | --- | --- | --- | --- | --- | --- | --- |
| **Los Angeles County** | | | | | | | | | | |
|  | **ASD by 4 Years** | | | | | **ASD by 8 Years** | | | | |
|  | **Cumulative Incidence** | | | **Average Age at Diagnosis** | | **Cumulative Incidence** | | | **Average Age at Diagnosis** | |
| **Birth Year** | **ASD Cases** | **Total Births** | **Percent** | **Mean (Yrs)** | **SD (Yrs)** | **ASD Cases** | **Total Births** | **Percent** | **Mean (Yrs)** | **SD (Yrs)** |
| 2018 | 3,108 | 110,055 | 2.82% | 3.38 | 0.49 | --- | --- | --- | --- | --- |
| 2017 | 2,651 | 116,332 | 2.28% | 3.31 | 0.47 | --- | --- | --- | --- | --- |
| 2016 | 2,540 | 122,319 | 2.08% | 3.32 | 0.47 | --- | --- | --- | --- | --- |
| 2015 | 2,242 | 123,818 | 1.81% | 3.32 | 0.48 | --- | --- | --- | --- | --- |
| 2014 | 2,019 | 129,487 | 1.56% | 3.29 | 0.46 | 2,931 | 129,487 | 2.26% | 4.17 | 1.50 |
| 2013 | 1,767 | 127,963 | 1.38% | 3.32 | 0.47 | 2,610 | 127,963 | 2.04% | 4.19 | 1.45 |
| 2012 | 1,596 | 131,053 | 1.22% | 3.33 | 0.48 | 2,458 | 131,053 | 1.88% | 4.29 | 1.51 |
| 2011 | 1,482 | 129,946 | 1.14% | 3.30 | 0.48 | 2,318 | 129,946 | 1.78% | 4.32 | 1.55 |
| 2010 | 1,530 | 133,030 | 1.15% | 3.33 | 0.50 | 2,240 | 133,030 | 1.68% | 4.19 | 1.46 |
| 2009 | 1,335 | 139,264 | 0.96% | 3.31 | 0.49 | 2,073 | 139,264 | 1.49% | 4.27 | 1.49 |
| 2008 | 1,243 | 147,208 | 0.84% | 3.40 | 0.49 | 1,930 | 147,208 | 1.31% | 4.33 | 1.47 |
| 2007 | 1,138 | 151,282 | 0.75% | 3.40 | 0.49 | 1,861 | 151,282 | 1.23% | 4.45 | 1.53 |
| 2006 | 1,041 | 151,415 | 0.69% | 3.37 | 0.49 | 1,616 | 151,415 | 1.07% | 4.34 | 1.50 |
| 2005 | 1,105 | 149,803 | 0.74% | 3.35 | 0.49 | 1,590 | 149,803 | 1.06% | 4.19 | 1.47 |
| 2004 | 1,130 | 151,227 | 0.75% | 3.33 | 0.49 | 1,609 | 151,227 | 1.06% | 4.11 | 1.39 |
| 2003 | 1,116 | 151,881 | 0.73% | 3.36 | 0.49 | 1,587 | 151,881 | 1.04% | 4.09 | 1.31 |
| 2002 | 972 | 150,618 | 0.65% | 3.42 | 0.51 | 1,473 | 150,618 | 0.98% | 4.26 | 1.36 |
| 2001 | 828 | 152,944 | 0.54% | 3.41 | 0.50 | 1,312 | 152,944 | 0.86% | 4.38 | 1.47 |
| 2000 | 807 | 157,045 | 0.51% | 3.43 | 0.51 | 1,242 | 157,045 | 0.79% | 4.34 | 1.45 |
| 1999 | 690 | 155,665 | 0.44% | 3.46 | 0.51 | 1,076 | 155,665 | 0.69% | 4.38 | 1.47 |
| 1998 | 668 | 158,585 | 0.42% | 3.52 | 0.51 | 1,069 | 158,585 | 0.67% | 4.43 | 1.41 |
| 1997 | 573 | 161,599 | 0.35% | 3.54 | 0.51 | 984 | 161,599 | 0.61% | 4.49 | 1.35 |
| 1996 | 445 | 168,299 | 0.26% | 3.44 | 0.51 | 854 | 168,299 | 0.51% | 4.66 | 1.50 |
| 1995 | 399 | 173,938 | 0.23% | 3.49 | 0.53 | 730 | 173,938 | 0.42% | 4.66 | 1.52 |
| 1994 | 347 | 180,275 | 0.19% | 3.51 | 0.51 | 665 | 180,275 | 0.37% | 4.75 | 1.55 |
| 1993 | 337 | 189,535 | 0.18% | 3.52 | 0.56 | 606 | 189,535 | 0.32% | 4.65 | 1.50 |
| 1992 | 284 | 197,134 | 0.14% | 3.51 | 0.57 | 532 | 197,134 | 0.27% | 4.70 | 1.54 |
| 1991 | 254 | 202,378 | 0.13% | 3.52 | 0.50 | 441 | 202,378 | 0.22% | 4.58 | 1.46 |
| 1990 | 182 | 203,546 | 0.09% | 4.00 | 0.00 | 348 | 203,546 | 0.17% | 4.91 | 1.20 |

| (Table S7. continued) | | | | | | | | | | |
| --- | --- | --- | --- | --- | --- | --- | --- | --- | --- | --- |
| **Orange County** | | | | | | | | | | |
|  | **ASD by 4 Years** | | | | | **ASD by 8 Years** | | | | |
|  | **Cumulative Incidence** | | | **Average Age at Diagnosis** | | **Cumulative Incidence** | | | **Average Age at Diagnosis** | |
| **Birth Year** | **ASD Cases** | **Total Births** | **Percent** | **Mean (Yrs)** | **SD (Yrs)** | **ASD Cases** | **Total Births** | **Percent** | **Mean (Yrs)** | **SD (Yrs)** |
| 2018 | 316 | 35,493 | 0.89% | 3.21 | 0.41 | --- | --- | --- | --- | --- |
| 2017 | 297 | 37,093 | 0.80% | 3.18 | 0.39 | --- | --- | --- | --- | --- |
| 2016 | 293 | 37,847 | 0.77% | 3.19 | 0.39 | --- | --- | --- | --- | --- |
| 2015 | 268 | 37,328 | 0.72% | 3.21 | 0.40 | --- | --- | --- | --- | --- |
| 2014 | 224 | 38,315 | 0.58% | 3.20 | 0.40 | 353 | 38,315 | 0.92% | 4.25 | 1.56 |
| 2013 | 208 | 36,963 | 0.56% | 3.25 | 0.43 | 339 | 36,963 | 0.92% | 4.46 | 1.71 |
| 2012 | 234 | 37,859 | 0.62% | 3.24 | 0.42 | 372 | 37,859 | 0.98% | 4.30 | 1.57 |
| 2011 | 194 | 37,853 | 0.51% | 3.32 | 0.47 | 325 | 37,853 | 0.86% | 4.57 | 1.71 |
| 2010 | 188 | 38,039 | 0.49% | 3.26 | 0.44 | 322 | 38,039 | 0.85% | 4.45 | 1.60 |
| 2009 | 165 | 40,163 | 0.41% | 3.34 | 0.47 | 291 | 40,163 | 0.72% | 4.64 | 1.70 |
| 2008 | 178 | 42,286 | 0.42% | 3.26 | 0.44 | 301 | 42,286 | 0.71% | 4.49 | 1.67 |
| 2007 | 153 | 43,888 | 0.35% | 3.31 | 0.47 | 251 | 43,888 | 0.57% | 4.37 | 1.50 |
| 2006 | 159 | 44,021 | 0.36% | 3.28 | 0.45 | 250 | 44,021 | 0.57% | 4.38 | 1.65 |
| 2005 | 174 | 43,872 | 0.40% | 3.29 | 0.47 | 258 | 43,872 | 0.59% | 4.18 | 1.47 |
| 2004 | 173 | 44,927 | 0.39% | 3.30 | 0.47 | 255 | 44,927 | 0.57% | 4.23 | 1.54 |
| 2003 | 152 | 45,189 | 0.34% | 3.38 | 0.50 | 240 | 45,189 | 0.53% | 4.30 | 1.42 |
| 2002 | 140 | 44,579 | 0.31% | 3.24 | 0.43 | 217 | 44,579 | 0.49% | 4.17 | 1.41 |
| 2001 | 130 | 45,281 | 0.29% | 3.24 | 0.46 | 200 | 45,281 | 0.44% | 4.24 | 1.54 |
| 2000 | 118 | 46,833 | 0.25% | 3.26 | 0.46 | 190 | 46,833 | 0.41% | 4.48 | 1.75 |
| 1999 | 167 | 46,325 | 0.36% | 3.25 | 0.43 | 230 | 46,325 | 0.50% | 3.97 | 1.34 |
| 1998 | 127 | 46,048 | 0.28% | 3.27 | 0.44 | 177 | 46,048 | 0.38% | 4.00 | 1.31 |
| 1997 | 138 | 46,046 | 0.30% | 3.47 | 0.50 | 198 | 46,046 | 0.43% | 4.23 | 1.36 |
| 1996 | 85 | 39,501 | 0.22% | 3.44 | 0.50 | 153 | 39,501 | 0.39% | 4.64 | 1.60 |
| 1995 | 74 | 35,876 | 0.21% | 3.49 | 0.50 | 122 | 35,876 | 0.34% | 4.45 | 1.40 |
| 1994 | 51 | 37,104 | 0.14% | 3.51 | 0.50 | 102 | 37,104 | 0.27% | 4.96 | 1.72 |
| 1993 | 35 | 37,982 | 0.09% | 3.63 | 0.49 | 73 | 37,982 | 0.19% | 4.93 | 1.53 |
| 1992 | 27 | 38,499 | 0.07% | 3.59 | 0.50 | 65 | 38,499 | 0.17% | 5.05 | 1.53 |
| 1991 | 24 | 37,975 | 0.06% | 3.42 | 0.50 | 68 | 37,975 | 0.18% | 5.28 | 1.66 |
| 1990 | 24 | 37,515 | 0.06% | 4.00 | 0.00 | 41 | 37,515 | 0.11% | 5.00 | 1.38 |

| (Table S7. continued) | | | | | | | | | | |
| --- | --- | --- | --- | --- | --- | --- | --- | --- | --- | --- |
| **San Diego-Imperial** | | | | | | | | | | |
|  | **ASD by 4 Years** | | | | | **ASD by 8 Years** | | | | |
|  | **Cumulative Incidence** | | | **Average Age at Diagnosis** | | **Cumulative Incidence** | | | **Average Age at Diagnosis** | |
| **Birth Year** | **ASD Cases** | **Total Births** | **Percent** | **Mean (Yrs)** | **SD (Yrs)** | **ASD Cases** | **Total Births** | **Percent** | **Mean (Yrs)** | **SD (Yrs)** |
| 2018 | 1,162 | 42,833 | 2.71% | 3.30 | 0.47 | **---** | **---** | **---** | **---** | **---** |
| 2017 | 1,126 | 44,090 | 2.55% | 3.33 | 0.48 | **---** | **---** | **---** | **---** | **---** |
| 2016 | 1,097 | 45,579 | 2.41% | 3.21 | 0.41 | **---** | **---** | **---** | **---** | **---** |
| 2015 | 908 | 47,010 | 1.93% | 3.27 | 0.45 | **---** | **---** | **---** | **---** | **---** |
| 2014 | 696 | 47,515 | 1.46% | 3.22 | 0.46 | 985 | 47,515 | 2.07% | 4.07 | 1.51 |
| 2013 | 615 | 46,616 | 1.32% | 3.18 | 0.40 | 927 | 46,616 | 1.99% | 4.16 | 1.54 |
| 2012 | 577 | 47,339 | 1.22% | 3.20 | 0.40 | 812 | 47,339 | 1.72% | 4.08 | 1.53 |
| 2011 | 496 | 46,702 | 1.06% | 3.30 | 0.46 | 733 | 46,702 | 1.57% | 4.25 | 1.55 |
| 2010 | 481 | 48,182 | 1.00% | 3.38 | 0.49 | 683 | 48,182 | 1.42% | 4.18 | 1.46 |
| 2009 | 408 | 48,125 | 0.85% | 3.30 | 0.48 | 579 | 48,125 | 1.20% | 4.10 | 1.41 |
| 2008 | 384 | 50,380 | 0.76% | 3.35 | 0.48 | 542 | 50,380 | 1.08% | 4.22 | 1.54 |
| 2007 | 306 | 50,950 | 0.60% | 3.31 | 0.46 | 444 | 50,950 | 0.87% | 4.30 | 1.64 |
| 2006 | 294 | 49,986 | 0.59% | 3.48 | 0.50 | 398 | 49,986 | 0.80% | 4.13 | 1.27 |
| 2005 | 272 | 48,876 | 0.56% | 3.49 | 0.51 | 366 | 48,876 | 0.75% | 4.11 | 1.26 |
| 2004 | 209 | 48,712 | 0.43% | 3.35 | 0.48 | 337 | 48,712 | 0.69% | 4.28 | 1.37 |
| 2003 | 177 | 48,324 | 0.37% | 3.44 | 0.51 | 286 | 48,324 | 0.59% | 4.45 | 1.50 |
| 2002 | 137 | 46,575 | 0.29% | 3.30 | 0.49 | 243 | 46,575 | 0.52% | 4.63 | 1.70 |
| 2001 | 118 | 46,305 | 0.25% | 3.31 | 0.48 | 206 | 46,305 | 0.44% | 4.55 | 1.65 |
| 2000 | 122 | 46,884 | 0.26% | 3.37 | 0.48 | 201 | 46,884 | 0.43% | 4.50 | 1.61 |
| 1999 | 95 | 45,682 | 0.21% | 3.34 | 0.50 | 150 | 45,682 | 0.33% | 4.25 | 1.40 |
| 1998 | 88 | 46,059 | 0.19% | 3.44 | 0.50 | 121 | 46,059 | 0.26% | 4.07 | 1.20 |
| 1997 | 71 | 45,413 | 0.16% | 3.54 | 0.50 | 114 | 45,413 | 0.25% | 4.46 | 1.43 |
| 1996 | 63 | 47,064 | 0.13% | 3.46 | 0.50 | 108 | 47,064 | 0.23% | 4.44 | 1.36 |
| 1995 | 62 | 48,083 | 0.13% | 3.47 | 0.50 | 99 | 48,083 | 0.21% | 4.36 | 1.34 |
| 1994 | 63 | 50,245 | 0.13% | 3.52 | 0.53 | 104 | 50,245 | 0.21% | 4.50 | 1.44 |
| 1993 | 43 | 51,581 | 0.08% | 3.51 | 0.55 | 85 | 51,581 | 0.16% | 4.92 | 1.71 |
| 1992 | 49 | 53,533 | 0.09% | 3.37 | 0.64 | 88 | 53,533 | 0.16% | 4.56 | 1.59 |
| 1991 | 26 | 52,118 | 0.05% | 3.54 | 0.51 | 62 | 52,118 | 0.12% | 5.05 | 1.55 |
| 1990 | 19 | 46,836 | 0.04% | 4.00 | 0.00 | 48 | 46,836 | 0.10% | 5.25 | 1.34 |
| Notes: SD = Standard Deviation; ASD = Autism Spectrum Disorder; Yrs = Years | | | | | | | | | | |
